# Mitogenome-wise codon usage pattern from comparative analysis of the first mitogenome of *Blepharipa* sp. (Muga uzifly) with other Oestroid flies

**DOI:** 10.1038/s41598-022-10547-8

**Published:** 2022-04-29

**Authors:** Debajyoti Kabiraj, Hasnahana Chetia, Adhiraj Nath, Pragya Sharma, Ponnala Vimal Mosahari, Deepika Singh, Palash Dutta, Kartik Neog, Utpal Bora

**Affiliations:** 1 Bioengineering Research Laboratory, Department of Biosciences and Bioengineering, Indian Institute of Technology Guwahati, Guwahati, Assam India; 2 Centre for the Environment, Indian Institute of Technology Guwahati, Guwahati, Assam India; 3 Department of Bioengineering and Technology, Gauhati University Institute of Science and Technology (GUIST), Gauhati University, Guwahati, Assam India; 4 Biotechnology Section, Central Muga Eri Research & Training Institute (CMER&TI), Lahdoigarh, Jorhat, Assam India

**Keywords:** Evolutionary genetics, Molecular evolution, Ecosystem ecology

## Abstract

Uziflies (Family: Tachinidae) are dipteran endoparasites of sericigenous insects which cause major economic loss in the silk industry globally. Here, we are presenting the first full mitogenome of *Blepharipa* sp. (Acc: KY644698, 15,080 bp, A + T = 78.41%), a dipteran parasitoid of Muga silkworm (*Antheraea assamensis*) found in the Indian states of Assam and Meghalaya. This study has confirmed that *Blepharipa* sp. mitogenome gene content and arrangement is similar to other Tachinidae and Sarcophagidae flies of Oestroidea superfamily, typical of ancestral Diptera. Although, Calliphoridae and Oestridae flies have undergone tRNA translocation and insertion, forming unique intergenic spacers (IGS) and overlapping regions (OL) and a few of them (IGS, OL) have been conserved across Oestroidea flies. The Tachinidae mitogenomes exhibit more AT content and AT biased codons in their protein-coding genes (PCGs) than the Oestroidea counterpart. About 92.07% of all (3722) codons in PCGs of this new species have A/T in their 3rd codon position. The high proportion of AT and repeats in the control region (CR) affects sequence coverage, resulting in a short CR (*Blepharipa* sp.: 168 bp) and a smaller tachinid mitogenome. Our research unveils those genes with a high AT content had a reduced effective number of codons, leading to high codon usage bias. The neutrality test shows that natural selection has a stronger influence on codon usage bias than directed mutational pressure. This study also reveals that longer PCGs (e.g., *nad5*, *cox1*) have a higher codon usage bias than shorter PCGs (e.g., *atp8*, *nad4l*). The divergence rates increase nonlinearly as AT content at the 3rd codon position increases and higher rate of synonymous divergence than nonsynonymous divergence causes strong purifying selection. The phylogenetic analysis explains that *Blepharipa* sp. is well suited in the family of insectivorous tachinid maggots. It's possible that biased codon usage in the Tachinidae family reduces the effective number of codons, and purifying selection retains the core functions in their mitogenome, which could help with efficient metabolism in their endo-parasitic life style and survival strategy.

## Introduction

Insect mitochondria which arose from alpha-proteobacteria have its own circular mitogenome of about 14–20 kb^1–3^. The inner membrane of this organelle harbors five distinct protein complexes for efficient production of energy via oxidative phosphorylation (OXPHOS) process^4,5^. In general, the insect mitogenome has 13 protein-coding genes (PCGs), 2 ribosomal RNAs (rRNAs), 21 to 23 transfer RNAs (tRNAs)^6^. It also contains several non-coding regions with the lengthiest being AT-rich control region (Table 1)^7^. A typical metazoan mitogenome is small in size, maternally inherited, mutation prone, has minimal or no homologous recombination, with conserved gene content, and high genetic polymorphism, making it a potential  sequence for barcoding, phylogeography, phylogenetic and molecular dating research^8–10^. However, little attention has been paid to the study of mitochondrial codon alteration and its role in environmental adaptation^10,11^. Differential mitochondrial codon usage has been  probed mainly on vertebrates, whereas among invertebrates only some parasitic Platyhelminthes, ribbon worms and moths had been surveyed till date^12–14^.

**Table 1:** List of Diptera (n = 42) and Out group Lepidoptera (n = 2) used in this study for comparative mitogenomics and phylogenetic analysis (A = Ancestral mitogenome arrangement).

Sl no.	Accession no	Family	Organisms	Mito genome (bp)/AT%	CR (bp)/AT%	rRNA (bp)/AT%	tRNA (bp)/AT%	PCG size (bp)/AT%	Mito genome pattern	Common name	Economic importance	Lifestyle/food habit	References
1	NC_019632	Calliphoridae	*Chrysomya bezziana*	15,236	392	2116	1469	11,151	A + *trnI* + duplication CR	Old World screwworm	Causes Myiasis in animal and human	Obligate ectoparasite/necrophagous	^4,6,59^
				75.89	87.75	79.63	76.31	74.53					
2	NC_026996	Calliphoridae	*Aldrichina grahami*	14,903	89	2107	1475	11,118	A	Blow fly	Forensic insect, transmit human and animal pathogens	Necrophagous	^60–62^
				76.75	92.13	80.06	76.47	75.91					
3	NC_025338	Calliphoridae	*Chrysomya pinguis*	15,838	988	2114	1478	11,151	A + *trnI*	Blowfly	Forensically Important	Ectoparasite/necrophagous	^10–12^
				76.06	88.25	79.75	75.98	74.11					
4	NC_019631	Calliphoridae	*Chrysomya albiceps*	15,491	657	2113	1472	11,151	A + *trnI* + duplication CR	Hairy maggot blowfly	Cause secondary myiases	Necrophagous	^13,44^
				77.26	85.69	80.40	76.22	76.16					
5	NC_019636	Calliphoridae	*Protophormia terraenovae*	15,170	356	2112	1472	11,151	A	Northern blowfly	Myiasis pest of livestock	Ectoparasite/necrophagous	^4,14^
				75.87	90.73	80.06	76.01	74.44					
6	NC_019635	Calliphoridae	*Chrysomya saffranea*	15,839	994	2114	1472	11,151	A + *trnI* + duplication CR	Steelblue blowfly	Forensic insect and causes myiasis in human beings and animals	Necrophagous	^4,15^
				76.45	88.12	79.84	76.08	74.63					
7	NC_019638	Calliphoridae	*Hemipyrellia ligurriens*	15,938	1119	2115	1473	11,157	A	Blowfly	Forensic insect, myiasis in goat, buffalo and bull, vector of pathogens	Parasite/necrophagous	^4,16,17^
				77.35	89.72	80.14	76.98	75.50					
8	NC_019637	Calliphoridae	*Lucilia porphyrina*	15,877	1047	2115	1470	11,157	A	Porphyrina blow fly/Oriental blow fly	Forensic insect, myiasis in livestock, human	Ectoparasite/human or animal corpses/necrophagous	^4,11,18,19^
				76.26	88.92	79.57	76.46	74.26					
9	NC_019634	Calliphoridae	*Chrysomya rufifacies*	15,412	574	2114	1473	11,151	A + *trnI* + duplication CR	Hairy maggot blowfly	Forensic insect, myiasis in livestock	Necrophagous	^4^
				77.20	84.84	80.22	76.57	76.18					
10	NC_019633	Calliphoridae	*Chrysomya megacephala*	15,273	428	2114	1472	11,151	A + *trnI* + duplication CR	Oriental latrine fly	Forensic insect, myiasis in livestock	Necrophagous	^20^
				75.98	87.14	79.70	75.88	74.66					
11	NC_002660	Calliphoridae	*Cochliomyia hominivorax*	16,022	1175	2110	1470	11,157	CR A	New World screw-worm fly	Forensic insect, myiasis in mammals	Necrophagous	^63^
				76.90	90.80	79.81	76.59	74.72					
12	NC_002697	Calliphoridae	*Chrysomya putoria*	15,837	1008	2114	1471	11,154	A + *trnI* + duplication CR	Tropical African latrine blowfly	Forensic insect, myiasis in mammals	Necrophagous	^21^
				76.70	88.59	79.99	76.13	74.91					
13	NC_031381	Calliphoridae	*Chrysomya phaonis*	15,831	992	2112	1472	11,151	A + *trnI*	Blow flies	Medical and forensic importance	Necrophagous	^22^
				76.09	88.10	79.59	75.81	74.23					
14	NC_029486	Calliphoridae	*Lucilia coeruleiviridis*	14,989	168	2110	1471	11,145	A + Partial CR	Green bottle fly	Forensic insect, myiasis in pig and other mammals	Ectoparasite/necrophagous	^19,23^
				76.02	87.5	80	76.88	74.87					
15	NC_029215	Calliphoridae	*Calliphora chinghaiensis*	15,269	441	2113	1463	11,190	Translocation of *trnS1*	Blue bottle flies	Forensic importance	Necrophagous	^24,25^
				76.75	84.35	80.59	76.82	75.55					
16	NC_028411	Calliphoridae	*Calliphora vomitoria*	16,134	1319	2110	1471	11,151	A	Blue bottle fly	Forensic importance and causes myiasis	Necrophagous	^26,27,64^
				77.55	90.29	80.33	76.41	75.54					
17	NC_028412	Calliphoridae	*Chrysomya nigripes*	15,832	966	2115	1476	11,154	A + *trnI* + duplication CR	Blowfly	Forensic importance	Necrophagous	^22,65^
				76.92	88.09	80.14	76.01	75.24					
18	NC_028056	Calliphoridae	*Lucilia illustris*	15,954	1094	2153	1469	11,100	CR A	Green bottle fly	Forensic importance and myiasis in pet Animals	Ectoparasite/necrophagous	^19,66^
				77.42	90.85	79.74	76.85	75.60					
19	NC_028057	Calliphoridae	*Lucilia Caesar*	15,954	1117	2152	1469	11,121	CR A	Common greenbottle	Forensic importance and facultative wound myiasis	Ectoparasite/necrophagous	^19,67,68^
				77.30	90.59	79.73	76.78	75.39					
20	NC_013932	Oestridae	*Hypoderma lineatum*	16,354	1493	2101	1453	11,136	A	Common cattle grub/warble fly	Causes Myiasis in ruminants	Ectoparasite/sarcophagous/carnivore	^69–72^
				77.85	87.54	80.48	77.70	75.85					
21	NC_006378	Oestridae	*Dermatobia hominis*	16,360	1545	2112	1458	11,157	Insertion of *trnV* between *trnK-trnD*	Human botfly/tropical warble fly	Causes Myiasis in human, cattle, dogs and forensically importance	Endoparasites of birds and mammals/carnivore	^70,73–75^
				77.81	91.39	81.43	77.09	75.23					
22	NC_029812	Oestridae	*Gasterophilus pecorum*	15,750	1001	2048	1461	11,103	A	Horse botfly	Gastrointestinal myiasis in equines and forensic science	Obligate intestinal parasites/carnivore	^69,76^
				70.73	80.81	74.31	75.56	68.46					
23	NC_029834	Oestridae	*Gasterophilus intestinalis*	15,660	875	2107	1470	11,103	CR A	Horse botfly	Gastric myiasis in horse, donkey	Obligate internal parasites /carnivore	^77–79^
				70.16	80.8	73.84	74.69	67.88					
24	NC_026196	Sarcophagidae	*Ravinia pernix*	15,778	1750	2114	1470	11,154	A		Forensic importance, potential for myiasis	Endo-parasitoid/saprophagous	^3,60^
				77.17	84.34	80.36	76.32	75.46					
25	NC_026112	Sarcophagidae	*Sarcophaga melanura*	15,190	360	2108	1475	11,154	A	Flesh fly	Forensic importance, causes myiasis	Ectoparasite/saprophagous	^61,62,80,81^
				75.64	90.27	80.07	76.61	74.04					
26	NC_025944	Sarcophagidae	*Sarcophaga Africa*	15,144	338	2111	1469	11,151	A	Flesh fly	Intestinal myiasis and forensic science	Ectoparasite/saprophagous	^81–83^
				75.74	89.34	79.91	76.31	74.32					
27	NC_025574	Sarcophagidae	*Sarcophaga portschinskyi*	14,929	118	2109	1468	11,139	A	Flesh fly	Forensic importance	Ectoparasite/saprophagous	^81,84^
				76.18	89.83	80.41	76.08	75.12					
28	NC_025573	Sarcophagidae	*Sarcophaga similis*	15,158	354	2107	1461	11,139	A	Flesh fly	Responsible for myiasis and forensic importance	Ectoparasite/saprophagous	^81,85–87^
				76.36	87.57	80.25	76.11	75.21					
29	NC_023532	Sarcophagidae	*Sarcophaga peregrine*	14,922	123	2108	1470	11,139	A	Flesh fly	Responsible for myiasis and forensic importance	Ectoparasite/saprophagous	^81,88,89^
				74.97	87.80	79.83	76.12	73.61					
30	NC_017605	Sarcophagidae	*Sarcophaga impatiens*	15,169	359	2113	1469	11,154	A	Flesh fly	Forensic importance Carrion breeding	Ectoparasite/saprophagous	^81,90^
				74.76	88.30	79.46	76.37	73.08					
31	NC_026667	Sarcophagidae	*Sarcophaga crassipalpis*	15,420	613	2109	1484	11,153	A	Flesh fly	Forensic importance responsible of myiasis	Ectoparasite/saprophagous	^81,91,92^
				76.22	89.39	80.03	76.21	74.65					
32	NC_028413	Sarcophagidae	*Sarcophaga albiceps*	14,935	125	2111	1470	11,139	A	Flesh fly	Forensically important	Ectoparasite/saprophagous	^81,93^
				75.86	90.4	79.77	75.78	74.84					
33	**Current Study**	Tachinidae	***Blepharipa* sp.**	**15,080**	168	2143	1466	**11,166**	**A**	Uzi Fly	Endoparasite of muga silkworm	Endoparasite/parasitoid	This Study
				**78.41**	92.60	82.54	78.24	**77.27**					
34	NC_019640	Tachinidae	*Rutilia goerlingiana*	15,331	568	2101	1451	11,131	A	Tachinid flies	Insect endoparasite	Endoparasite/parasitoid	^4^
				77.70	91.07	81.81	77.18	76.11					
35	NC_018118^a^	Tachinidae	*Elodia flavipalpis*	14,932	105	2120	1463	11,154	A	Tachinid flies	Natural enemies of the leaf-roller moths	Endoparasite/parasitoid	^32^
				79.96	92.38	83.49	79.76	79.09					
36	NC_014704^a^	Tachinidae	*Exorista sorbillans*	14,960	105	2117	1471	11,136	A	Uzi Fly	Endoparasite of mulberry silkworm	Endoparasite/parasitoid	^42^
				78.44	98.09	81.76	76.75	77.64					
37	NC_016713	Agromyzidae	*Liriomyza bryoniae*	16,183	1354	2111	1468	11,169	A	Tomato leaf miner	Pest species of Tomato and other vegetables (Cucurbitaceae and Solanaceae,)	Ectoparasite /polyphagous/herbivore	^94–96^
				79.26	95.49	82.42	78.54	76.66					
38	NC_015926	Agromyzidae	*Liriomyza sativae*	15,551	741	2111	1465	11,160	A	Vegetable leafminer	Pest species vegetables	Ectoparasite /polyphagous/herbivore	^95,97^
				77.53	92.98	82.18	76.99	75.59					
39	NC_014402	Tephritidae	*Bactrocera minax*	16,043	1140	2115	1466	11,151	A	Oriental citrus fly	Pest of citrus and related genera of Rutaceae	Phytophagous/herbivore	^98,99^
				67.28	77.63	73.71	72.30	64.21					
40	NC_029468	Tephritidae	*Bactrocera umbrosa*	15,898	944	2120	1465	11,157	A	Oriental fruit fly	Pest of Moraceae family	Phytophagous/herbivore	^99,100^
				70.48	86.22	77.02	74.12	67.19					
41	NC_015079	Culicidae	*Culex pipiens pipiens*	14,856	0	2118	1475	11,187	Inversion (*trnA-trnR* = > *trnR-trnA*)	Culex Mosquito	Vector of multiple diseases (West Nile virus)	Free living/multivoltine	^101^
				77.63		82.24	78.98	76.46					
42	NC_027502	Culicidae	*Anopheles culicifacies*	15,330	498	2113	1474	11,199	Inversion (*trnA-trnR* = > *trnR-trnA*)	Anopheles Mosquito	Vector of multiple diseases	Free living/multivoltine	^102^
				78.44	92.57	82.06	78.56	77.04					
43	NC 002355	Lepidoptera (Order)	*Bombyx mori*	15,643	499	2158	1468	11,142	*trnM-trnI-trnQ*	Mulberry Silkworm	Economically beneficial in silk and textile	Phytophagous/herbivore	^103^
				81.32	95.39	84.80	81.40	79.50					
44	KU379695	Lepidoptera (Order)	*Antheraea assamensis*	15,272	328	2123	1465	11,175	*trnM-trnI-trnQ*	Muga Silkworm	Economically beneficial in silk and textile	Phytophagous/herbivore	^104^
				80.18	91.15	84.26	80.75	78.75					

^a^Mitogenomes were used for manual curation of *Blepharipa* sp.

Tachinidae is the largest family of Oestroidea superfamily containing about 10,000 enormously diversified, koinobiont, internal parasitoid flies with similar kind of phenotype and morphology due to which its taxonomical classification has always remained a challenge^15–17^. The Tachinid larva hides, feeds and respires inside the host larva and then quickly eats the host in the late larval or pupal stage, eventually killing their host^1,2^. The host range of tachinid flies differs extremely, and includes caterpillars, bugs, adult and larval beetles as well as a variety of other arthropods and non-arthropods^16–18^. However, the amount of biological information like host range, necessary habitat, mating system is known for only less than half of the species from this family^19,20^. Other Oestroidea flies have often been rigorously studied in forensic science and as a myiasis-causing agent of human and various domestic animals (Table 1). The Oestroidea flies are dependent on dead or living animals (necrophagous, sarcophagus, saprophagous) for the fulfillment of earlier stages of metamorphosis^16^. Among Oestroidea, Tachinids adopt a different survival strategy in the larval phase in which they are surrounded by an oxygen-limited environment and are vulnerable to host immune systems^19,21,22^. Uzi flies are Tachinids, responsible for infestation and death of commercially important silkworms. Four species of uzi flies are identified till date viz., the Japanese uzi fly, *Crossocosmia sericaria* (Rodani); the Hime uzi fly, *Ctenophora pavida* (Meigen); the Tasar uzi fly, *Blepharipa zebina* (Walker) and the Indian uzi fly, *Exorista sorbillans* (Wiedemann)^23^. The Indian sericulture industry (mulberry, muga and tasar) is heavily affected by the last two dipteran endo-parasites, causing economic loss to the rural seri based farmers in India^18,23,24^. The currently studied uzifly species, *Blepharipa* sp., found in Assam and Meghalaya, causes the death of muga silkworm (*A. assamensis*) larva during winter and post-winter season and has been accounted for around 80–90% yield loss in muga seed cultivating areas^25–27^.

Despite having the scientific importance of mitogenome and economic significance of Tachinid flies, only 4 mitogenomes of this family is available in the public databases till date (3 listed in Table 1). In this study, for the first time we present the complete mitogenome (mtDNA) sequence from Blepharipa genus (*Blepharipa* sp.) using next-generation sequencing (GenBank Acc No. KY644698). An extensive comparative analysis with various Oestroidea mitogenomes (Table 1) available in NCBI is also presented. For this analysis we considered several mitogenome physiognomies such as size, nucleotide composition bias, and gene arrangement among the Oestroidea flies and other outgroups. Our study also emphasized on mitochondrial codon usage pattern since every organism possesses a unique codon choice which is related to gene expression, translational efficiency, and further protein structure and function^28–31^. We found that whole mitogenome (WMG) and protein-coding genes (PCGs) of Tachinid flies are highly AT biased in nature than other flies which is in agreement with the report of Zhao et al*.*^32^. In conjunction, the 3rd codon positions are AT-rich, resulting in the use of fewer effective number of codons and maximum biased codons in the PCGs of this family. The substitution rate analysis of PCGs indicates that rate of synonymous divergence is higher than nonsynonymous divergence due to prevalence of purifying selection (dN/dS < 1) in branch leading to *Blepharipa* sp. as well as in background branches. Our study also ascertains that longer genes in mitochondria, such as *nad5*, *nad4*, *nad1*, and *cox1*, employ more biased codons than shorter genes (*nad4l*, *atp8*), which is also seen in intron-less prokaryotic protein-coding genes^33,34^. Neutrality test supports the role of natural selection in shaping codon choice in protein-coding genes. The regression analysis between nucleotide substitution rates and various codon usage indices suggests that a nonlinear model is more effective than a typical linear model in delineating relationships. It asserts that the rate of divergence rises with increasing AT concentration at the 3rd codon position along a nonlinear S-shape curve, and that synonymous divergence is higher than nonsynonymous divergence. The use of strongly biased codons by Tachinids leads to a reduction in the effective number of codons which may contribute to the efficient metabolism of endo-parasitic life strategies. Further, phylogenies of Oestroidea exhibited well-supported monophyly of Sarcophagidae and Calliphoridae family.

## Materials and method

### Sample collection, processing, sequencing, and assembly

The fully grown *Blepharipa* sp. pupa were obtained from the Central Muga Eri Research and Training Institute (CMER&TI), Jorhat, Assam, India (Lat: 26° 47′49.1″N Lon: 94° 19′35.0″E) with the Sample ID-CMERI-Uzi-001. The pupa was dissected, chopped, and stored in 95% absolute ethanol at − 80 °C freezer. The steps involving mitochondrial DNA isolation, library preparation to sequencing, and assembly were carried out at the Genotypic Technology Pvt. Ltd. Bangalore, India (http://www.genotypic.co.in/) and are briefly discussed here. Total DNA was extracted from tissues using CTAB (Cetyl Trimethyl Ammonium Bromide) based method and filtered by silica column (Genotypic Technology Pvt. Ltd. Bengaluru, India). The quality, quantity, and purity of isolated purified, DNA was tested using agarose gel electrophoresis, light absorption, and fluorescence spectroscopy.

The library preparation was performed by using Illumina-compatible NEXTFlex DNA library protocol (Cat #5140-02). Mitochondrial DNA was preferentially enriched through NEBNext microbiome DNA enrichment kit (New England Biolabs, USA) which selectively removed CpG-methylated eukaryotic nuclear DNA. The enriched mitochondrial DNA obtained was sheared to produce fragments of about 200–400 bp in Covaris microTube with the S220 system (Covaris, Woburn, Massachusetts, USA) through focused ultra-sonication. The fragment size distribution was determined using Agilent Tape Station with D1000 DNA Kit (Agilent Technologies, Santa Clara, California, USA). The resulting fragmented DNA was cleaned up by HighPrep magnetic beads (MagBio Genomics, Inc, Gaithersburg, Maryland) to remove salts, primers, primer-dimers, dNTPs, etc. The fragments were subjected to end-repair, A-tailing, and ligation of the Illumina multiplexing adaptors using the NEXTFlex DNA Sequencing kit (Catalogue # 5140–02, BioScientific), followed by purification of adaptor-ligated DNA sequence through HighPrep beads and amplification through PCR. The PCR cycling conditions followed include, the initial denaturation at 98 °C for 2 min; 10 cycles of denaturation at 98 °C for 30 s; annealing at 65 °C for 30 s followed by extension at 72 °C for 60 s; and a final extension at 72 °C for 4 min employing the primers supplied by NEXTFlex DNA Sequencing kit. Further, the amplified PCR product was purified via HighPrep beads, quantified using Qubit fluorometer (Thermo Fisher Scientific, MA, USA), and the fragment range was assessed using Agilent D1000 Tape (Agilent Technologies). Finally, the sequencing was performed using Illumina NextSeq500 (Illumina Inc, Sandiego, USA) through 2 × 150 bp paired-end chemistry. The raw paired-end reads were de-multiplexed using Bcl2fastQ (V2), and the quality was assessed with FastQC v2.2 tool^35^. The Illumina raw reads were processed by in-house Perl script (ABLT-Scripts (no version available), Genotypic technology, Bangalore India) for the removal adapters and low-quality bases (Q < 30) towards 3′-end. The SPAdes-3.6.0 (St. Petersburg genome assembler) was used for de novo assembly of reads^36,37^, the scaffolding of assembled contigs and clustering were carried out with SSPACE (v 2.0) and CAP3 (Version Date: 10/15/07) programs^38,39^. The closest reference species was identified by BLAST (online blast was used) analysis of assembled scaffold against NCBI nr (non-redundant) database and the alignment of scaffold against reference sequence was done through Bowtie2 (v 2.2.7)^40^. The aligned data was processed using SAMtools (last used July, 2016) for generating reference assisted consensus sequences^41^. Final scaffolding was done in SSPACE using that reference assisted consensus sequence along with spades assembly-based scaffold to correct the regions having N's in the initial scaffold. All tools were run on default parameters. The assembly was then validated using a PCR-based technique on two regions: *nad6* (protein-coding gene) and the control region (AT rich region), followed by Sanger sequencing (see Method in Supplementary Note). According to previous reports on Tachinids, NGS sequencing had significantly lower coverage in the control region (CR) compared to other species groups, which was attributed to AT rich bases, lowering the correctness and completeness of Tachinid mitochondrial genome assemblies^32,42^. Hence, we designed primer sets as per Bronstein et al*.* targeting the CR of *Blepharipa* sp.^43^ (see Method in Supplementary Note). In addition, we mapped Illumina reads to the assembly to inspect the depth of coverage across the control region using Bowtie2 (v 2.4.4)^40^.

### Mitogenome annotation and documentation

**Figure 1:**
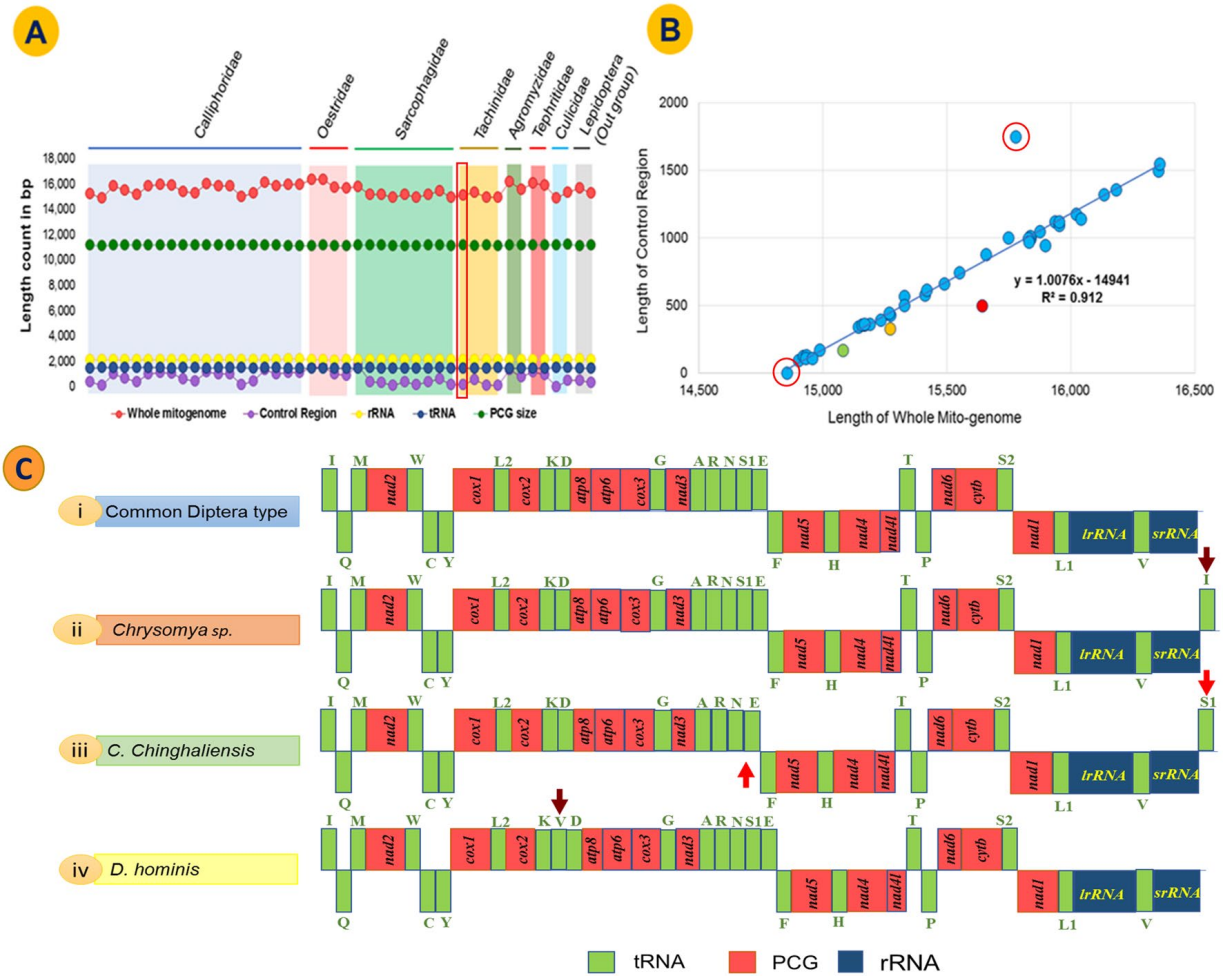
Size and arrangement of genes in the mitogenome; (**A**) Whole mitogenome (WMG), Protein-coding genes (PCG), tRNA, rRNA and Control region (CR) length variation among Oestroidea Superfamily, Red marked region *Blepharipa* sp. (**B**) Relation between WMG and CR length (R^2^ = 0.912 p < 0.001). Green bubble = *Blepharipa* sp., Yellow bubble = *Antheraea assamensis*, Red bubble = *Bombyx mori*. The isolated bubbles marked in red circle represents *Ravinia pernix* and the *Culex pipiens pipiens* (see “Mitogenome annotation and documentation” section). (**C**) Gene arrangement of *Blepharipa* sp. mitogenome (i), a common ancestral Diptera type with respect to other selected exceptional arrangement of Oestroidea superfamily (ii, iii, iv). Downward brown arrow = Insertion of tRNA; Upward-downward red arrow = translocation of tRNA. The J strand genes were shown in upward direction and the N strand genes were downward direction.

The assembled scaffolds were annotated using MITOS WebServer^44^ (last accessed April 2017). The PCG boundaries (start and stop codons) were determined through NCBI ORF Finder (last accessed April–May 2017) based on the invertebrate mitochondrial genetic code^45^. Additionally, gene boundaries, overlapping and intergenic spacer regions were estimated through NCBI BLAST (last accessed April–May 2017), BioEdit v. 7.2, and ClustalW program of Mega 7.0 software using reference sequences from other published Dipteran mitogenomes^46–48^. The control region (CR) was confirmed by comparing it with the available sequences in GenBank^49^. The secondary structures of tRNAs were predicted through MITOS Server and confirmed using tRNAscan-SE tool (see Fig. S7 in Supplementary Note)^50^. The secondary structures of mitochondrial rRNAs were examined by using Mfold Web Server^51^ (last accessed May 2017). Finally, the annotated file of *Blepharipa* sp. mitogenome was prepared through the NCBI Sequin tool, and SRA data along with the sequin file were submitted to NCBI GenBank (Acc No.: KY644698)^52^. Additionally, for comparative analysis, mitogenome sequences and annotations of other 43 species were downloaded from NCBI (Table 1). It is visible from Fig. 1B that *Culex pipiens pipiens* (0 bp) and *Ravinia pernix* (1750 bp) display anomalies in their CR size. However, it may be due to an error in NCBI annotation as the associated literature of *R. pernix* had documented the CR size as 965 bp^3^.

### Sequence alignment and phylogenetic inference

To obtain the molecular phylogeny of Oestroidea flies, especially among 4 four distinct families (Calliphoridae, Sarcophagidae, Oestridae, and Tachinidae) listed in (Table 1), were selected to use in phylogenetic analysis, including 2 species from each of the Tephritidae, Agromyzidae, Culicidae family, and 2 species from the order Lepidoptera (*B. mori* and *A. assamensis*) as an outgroup. The translated nucleotide sequences of each PCGs were aligned using MAFFT v. 5 algorithm in TranslatorX server (http://translatorx.co.uk/; last accessed July 2017), which were again back translated^53,54^. The rRNAs were aligned via Clustal Omega and tRNAs were aligned via Clustal W^55^. After that, individual aligned PCGs (rRNAs and tRNAs not included) were concatenated using the nexus module of the Bio-python programme^56^. Substitution model optimization for the dataset was performed in jModelTest 2.1.7^57^. The Bayesian analysis of the dataset was conducted with MrBayes v3.2.6 based on the Markov chain Monte Carlo (MCMC) method for 2,000,000 generations^58^. Two independent runs with four chains (one cold and three heated chains) were sampled every 1000 MCMC steps. A 50% majority-rule consensus tree was built after discarding the initial 10% as burn-in, and node supports were analyzed based on posterior probabilities (PP). Other parameters like effective sample size (ESS > 200) and potential scale reduction factor (PSRF) were evaluated for stationary using Tracer v1.6^105^. The Maximum Likelihood analysis was executed using RAxML 8.2.x with 5000 bootstrap replicates and the rapid bootstrap feature (random seed value 12345)^106^. The individual gene trees for 13 PCGs also estimated similarly through RaxML 8.2.x with 5000 bootstrap replicates. Finally, the consensus phylogenetic trees for the dataset were visualized and edited using iToL v3.6.1 tool^107^. To create a contour map, RaxML cladogram tree was generated using Figtree v1.4.4 (https://www.softpedia.com/get/Science-CAD/FigTree-AR.shtml/) and used as a reference tree for contMap function in the R v. 4.0.2 environment using package Phytools^108^.

### Nucleotide content, skew and substitution analysis

The nucleotide composition of the whole mitochondrial genome, concatenated and individual PCGs, tRNAs, rRNAs, intergenic spacers, and control region was calculated using MEGA 7.0 software^48^. The base composition skewness was also calculated for all the regions of mitogenome using the formula (Eqs. (1) and (2))^21^.

1 $${\text{AT skew }} = \, \left( {{\text{A}} - {\text{T}}} \right)/ \, \left( {{\text{A}} + {\text{T}}} \right)$$

2 $${\text{GC skew }} = \, \left( {{\text{G}} - {\text{C}}} \right)/ \, \left( {{\text{G}} + {\text{C}}} \right)$$

where A, T, G, and C denote the frequencies of respective bases.

Further gene alignments, consensus species tree, and individual gene trees were used for the investigation of molecular evolution. The analysis was constrained only to the branch of interest and we used a gene-level approach based on the ratio of nonsynonymous (dN) to synonymous (dS) substitutions rate (ω = dN/dS) to detect possible diversifying selection, via likelihood ratio tests through CODEML algorithm from the PAML package^109^. We tested branch-specific models M0, the simplest model, which has a single ω ratio for the entire tree. Further, we used two-ratio models that allow two different ω ratios for background and foreground lineage. In this study, we used lineage belonging to *Blepharipa* sp. as a foreground branch for both types of trees (gene tree and species tree). The significance level for these LRTs (likelihood ratio test) was measured using a χ^2^ approximation, where twice the difference of log-likelihood between the models (2ΔlnL) would be asymptotic to a χ^2^ distribution, with the number of degrees of freedom corresponding to the difference in the number of parameters between the models. Lineage-specific ω value was estimated for each branch through Model = 1. Synonymous and non-synonymous divergence rates (dS and dN) was calculated as pairwise manner implementing F3X4 codon frequencies.

The comparison of the control region (CR), overlapping region (OL), and Intergenic spacer (IGS) of *Blepharipa* sp. was carried out with the selected organisms based on the nucleotide identity, length, and location annotation from NCBI. The multiple sequence alignment was performed using Clustal Omega (the online version) and the conserved regions, repeats, and indels in these regions were visualized using BioEdit47^55^.

### Codon usage indices calculation and analysis

Initially, we calculated relative synonymous codon usage (RSCU) of amino acid using MEGA 7.0; which was further confirmed and batch calculation were carried out by DAMBE 6.4.67^48,110^. The cluster analysis of RSCU values was done using CIMminer web tool^111^ (last accessed August 2017). Principle component analysis of RSCU values was carried out in R v. 4.0.2 environment using ggfortify package (https://cran.r-project.org/web/packages/ggfortify/index.html/).

Different codon usage indices related to nucleotide composition namely, total of Guanine and Cytosine of any gene (GC), Average of GC at 1st and 2nd codon positions (GC12), GC at 3rd codon position (GC3), and GC content at 3rd codon position for the synonymous codons (GC3s) were calculated. The GC, GC12, GC3 were measured using MEGA 7.0^48^, and GC3s was estimated through CodonW (version 1.4.2, http://codonw.sourceforge.net/).

To measure the effective number of codons (ENc), we have followed the calculation of ENc from the study of Sun et al. in (2012) and estimated through DAMBE 6.4.67 software^110,112^. ENc designates the degree of codon bias for genes; where it computes deviation from uniform codon usage without any prior dependency over the sequence length or specific information of preferred codons^113^. The ENc values range between 20 to 61 and in general, values lesser than 35 signifies strong codon bias^114,115^. To detect different influencing factors of codon usage pattern among the genes in different organisms ENc vs GC3s (ENc-plot) graph was plotted using R v. 3.4.4^112,114^. The standard curve shows the functional relation between ENc and GC3s was under mutation pressure rather than selection^116^.

The neutrality test is a plot of GC12 against GC3 (GC12 vs GC3) for demonstrating the relationship between GC12 and GC3, and then investigating the mutation-selection equilibrium in forming the codon usage bias (CUB)^117,118^. The synonymous mutation frequently happens in the 3rd position of codons without changing the amino acid, whereas less frequent nonsynonymous mutations occur in 1st and 2nd positions^116^. Therefore, mutation in the 3rd position of codon is neutral and change in GC content at 1st or the 2nd positions would be correlated with the 3rd codon position if the mutation rate is similar in GC3 and GC12. This indicates that without any external pressure, the occurrence of mutations would be random rather than in a certain direction under the condition of pressure toward higher or lower GC content^117^. Thus, the base composition is similar and there is no variation across three codon positions; but, in the presence of external selection pressure, the base preferences would differ at individual codon positions^116,117^. In the neutrality plot, each gene is represented by discrete points, and when the points are placed along the diagonal line (slope of unity), GC12 is equally neutral to selection as GC3. It means that there will be no significant difference in the rate of mutation between three codon positions due to strong directional mutational pressure and lacks or only a weak external selection pressure^116,119^. Alternatively, as the regression slope of the plot approaches zero or parallel to the horizontal axis, the correlation between GC12 and GC3 declines due to the low mutation rate in GC12^116,120^. Therefore, the Neutrality plot would be crucial in determining the neutral degree while evaluating evolutionary factors.

### Regression modelling for determining the relationship between substitution rates and codon usage indices

To demonstrate the correlation between various substitution rates (dS, dN, and ω) and codon usage indices (GC3, GC3s, GC12, ENc) regression analysis namely linear model (LM), polynomial model (PM), and generalized additive model (GAM) were fitted on a univariate model. All statistical analysis was done using R v. 4.0.2.

Linear regression model forms a straight line between the dependent and independent variables^121^:

3 $$E\left( Y \right) = \, \beta_{0} + \, \beta_{1} X \, + \, \varepsilon$$

where Y is the dependent variable, E(Y) is the expected value of Y, β_0_ is the intercept, β_1_ is the coefficient of X (predictors) and ε is the residual.

Polynomial regression models use the approach of polynomial least squares to fit a non-linear relationship between the dependent and independent variables as an nth degree polynomial^122^:

4 $$E\left( Y \right) = \, \beta_{0} + \, \beta_{1} X \, + \, \beta_{2} X_{2} + \cdots + \, \beta_{n} X_{k} + \, \varepsilon$$

where Y is the dependent variable, E(Y) is the expected value of Y, β_0_ is the intercept, β_1_, β_2_, β_n_ is the coefficient of X (predictors), k is the degree of polynomial and ε is the residual. We used the poly_degree function from the npbr package in R v. 4.0.2 (https://cran.r-project.org/web/packages/npbr/index.html) for choosing optimal polynomial degrees via the BIC and AIC criterion.

GAM is an additive modelling technique that employs a sum of smoothing functions to represent the predictor variables, and it was fitted using the package mgcv (https://cran.r-project.org/web/packages/mgcv/index.html)^123,124^:

5 $$g\left( {E\left( Y \right)} \right) \, = a + f_{1} \left( {X_{1} } \right) \, + f_{2} \left( {X_{2} } \right) \, + \cdots + f_{n} \left( {X_{n} } \right) \, + \, \varepsilon$$

where Y is the dependent variable, E(Y) is the expected value of Y, g(Y) is a link function, a is the intercept, f_(1) (X_(1)) + f_(2) (X_(2)) + ⋯ + f_(n) (X_(n)) is the smooth function of predictors, and ε is the residual. Here, we utilized thin plate regression splines (default in mgcv) as a smoothing function and the default Gaussian family with the identity link function. All models were plotted using ggplot2 package (https://cran.r-project.org/web/packages/ggplot2/index.html) in R v. 4.0.2.

## Result and discussion

### Outcome of DNA sequencing, assembly, and validation

In this study, initially total DNA was isolated from the finely chopped, full-grown pupa of *Blepharipa* sp. The NanoDrop spectrophotometer (1294 ng/μl) and the Qubit fluorometer (732.8 ng/μl) both found that the concentration of total DNA in the sample at an optimum level for mitochondrial DNA enrichment. The Tape Station profile showed that the size of the fragments of the mitogenomic library were in the range of 250 to 550 bp. The complete insert size distribution ranged from 130 to 430 bp, with the combined adapter size being ~ 120 bp with mitogenome fragments. The appropriate distribution of fragments and their concentrations (~ 27.1 ng/μl) were also found to be suitable for sequencing. Sequencing through Illumina NextSeq500 yielded 4,402,752 raw reads, of which around 3,663,404 high-quality reads were retained after post-quality filtering. The final scaffolding and assembly of contigs generated a 15,080 bp single scaffold MtDNA in *Blepharipa sp.* (N50 = 15,080).

The sequencing outcome was validated by performing PCR amplification of one of the protein-coding genes, in this case, *nad6*. Where PCR amplification resulted in a single band of expected amplicon size (shown in Supplementary Method Online). Sanger sequencing and subsequent alignment of these amplicons showed almost 92% sequence similarity to our assembled *Blepharipa* sp. *nad6* gene (see Supplementary Method Online)*.* This provided strong evidence that our mitogenome assembly is reliable and can be used for general applications of mitochondrial genes, e.g., as a biomarker. The second mitogenomic region, the control region (CR) was suggested by the reviewer. We have discussed that CRs constitute repetitive A + T regions (“AT richness of Control Region and role of sequencing method” and “Impact of repeats on different sequencing technologies and assembly method” section). One or more repetitive regions within the CR identified in certain species (e.g. fish, human) have shown undesirable effects on PCR amplification and sequencing^125,126^. Many organisms have segmental duplications in CR induced by the appearance of pseudogenes that PCR can co-amplify^127–131^. Due to these associated problems, researchers generally rely on protein or ribosomal RNA genes for phylogenetics instead of CRs^132–134^. In this case, we also faced problems validating the CR. The PCR and gel electrophoresis using external PCR primers did not show a desirable single band as seen for *nad6*. As an alternative strategy, we used two pairs of primers, CR int_fwd and CR int_rev, internal primers, with CR15fwd and CR08rev primers, to perform a two-way sequencing of each amplicon, which generated multiple bands (see Supplementary Method Online, Figs. S1, S2). The most prominent bands were subjected to sequencing and yielded two mixed sequences, the best of which exhibited nearly 54% sequence resemblance with the *Blepharipa* sp. control region (see Method in Supplementary Note)*.* Further mapping of the Illumina reads with the assembly revealed that the depth of coverage across the CR was not as deep as that of protein-coding genes such as *cox2*, and it was also not inflated only over a repeated section of the CR. The depth over 1–112 varied from 5 to 20×, and that for the 15,025–15,080 bp was around 30×. We did observe that our reads didn't cover a 10 bp stretch of CR around 15,030–15,040 bp (see Method in Supplementary Note and Figs. S3–S6). We believe that our sequencing and assembly experiment was able to cover the majority of CR successfully with reasonable coverage barring that 10 bp stretch. Our results corroborate with the difficulties of CR sequencing seen with other species, and while this doesn’t reflect on the quality of our whole mitogenome assembly, researchers using mitogenomic CR regions for any kind of phylogenetic inference should proceed with caution.

### Size and organization of mitogenome

#### *Blepharipa* sp. mitogenome organization and structure

**Figure 2:**
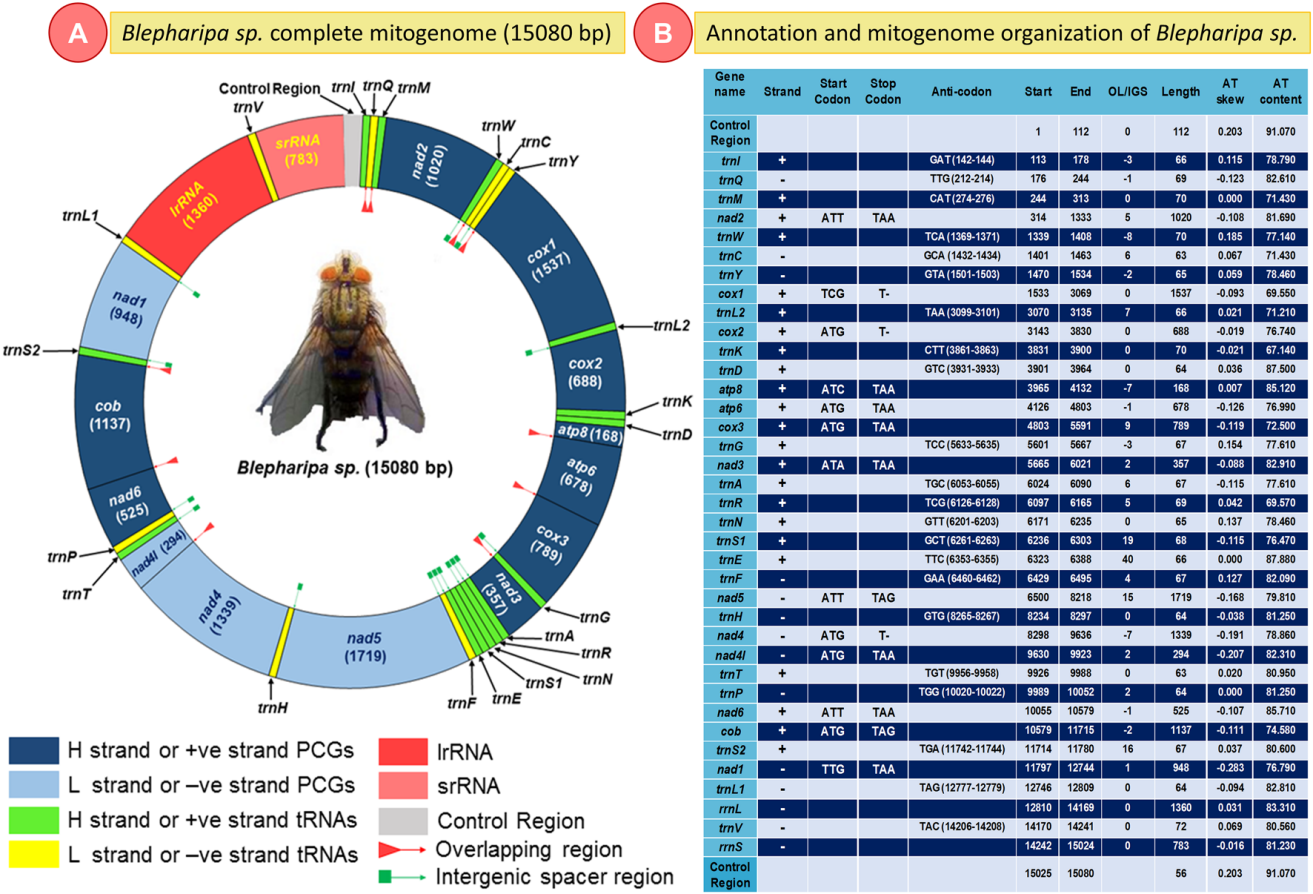
Complete mitochondrial genome structure of *Blepharipa* sp.; (**A**) Circular Map (**B**) Annotation and genome organization of mitogenome. tRNAs are represented as trn followed by the IUPAC-IUB single letter amino acid codes e.g., *trnI* denote *tRNA-Ile*.

The newly sequenced mitochondrial genome of *Blepharipa* sp. is closed circular and has a size of 15,080 bp, which falls within the typical insect mitogenome size (14 to 20 kb)^135–137^. Similar to other sequenced bilaterian mitogenomes, the *Blepharipa* sp. mitogenome has conventional gene content, a total of 37 genes (viz. 13 PCGs, 22 tRNAs, 2 rRNAs) and an AT-rich control region (CR) (Fig. 2A)^138–141^. Among these, 23 genes are present on the major strand (J strand or +ve strand), while the remaining 14 genes are present in the minor strand (N strand or –ve strand). The intron-less 13 PCGs are also separately encoded by these two strands, 9 PCGs (*nad2, cox1, cox2, atp8, atp6, cox3, nad3, nad6, cytb*) from the J strand and 4 PCGs (*nad5, nad4, nad4l, nad1*) from N strand covering 6899 bp and 4300 bp respectively constituting around 74.31% of the entire mitogenome (Fig. 2). The largest PCG present in this organism is *nad5* (1716 bp), and the smallest one is the *atp8* (165 bp)*.* Excluding stop codons, the J strand has 2237 codons, and the N strand has 1430 codons. Apart from *cox1* (TCG) and *nad1* (TTG), 11 PCGs follow the canonical “ATN” start codon. Ten PCGs of this mitogenome have “TAA or TAG” as their stop codon except for *cox1*, *cox2*, and *nad4*, where they end with an incomplete stop codon, a single T (Fig. 2**)**^142^. A total of 22 tRNAs are interspersed all over the entire mitogenome, ranging from 63 bp (*trnT*) to 72 bp (*trnV*) in size. The J and N strands have 14 tRNAs and 8 tRNAs, respectively, with 928 bp and 528 bp of nucleotides. Typical clover-leaf shaped secondary structures of tRNAs have been observed with a few exceptions where *trnC*, *trnF*, *trnP*, and *trnN* lack a stable TΨC loop see Supplementary Fig. S7 online). Two N-strand rRNAs with nucleotides of 1360 bp and 783 bp are transcribed individually for *rrnL* and *rrnS* (Fig. 2B).

This mitogenome has 10 gene boundaries where genes overlap with adjacent genes, varying from 1 to 8 bp in length, for a total of 35 bp. The longest overlapping sequence of 8 bp is present over the *trnW* and *trnC* genes. Likewise, the total length of all intergenic spacer sequences (excluding the control region) is 139 bp, present at 15 gene boundaries. The length of each intergenic spacer varies between 1 and 40 bp, and the longest one is located between the *trnE* and *trnF* genes. In this organism, eleven pairs of genes are located discreetly but adjacent to each other and any PCG adjacent to tRNA, ending with an incomplete stop codon (*cox1-trnL2*, *cox2-trnK*). The control region’s length of this dipteran fly is 168 bp, and the nature of this region is highly biased towards A + T content (Fig. 2).

#### Size comparison of Oestroidea mitogenome and their genes

To better understand the mitogenome of *Blepharipa* sp., it has been compared with the flies of the Oestroidea superfamily (blowflies, bot flies, flesh flies, uzi flies, and relatives). Various features have been taken into account for this comparison: mitogenome size, gene sizes, gene content, and how genes are placed in each mitogenome.

The mitogenome of eukaryotic organisms shows that there are significant size differences across mammals, fungi, and plants. The typical size of an animal mitogenome is near about 16 kb, a fungal mitogenome is 19–176 kb, and a plant mitogenome is far larger, with a size range of 200 to 2500 kb^143^. We have shown that the *Blepharipa* sp. whole mitogenome size (15,080 bp) is 416 bp smaller than the average Oestroidea flies mitogenome. As for the Oestroidea superfamily, *D. hominis* (human bot fly), an Oestridae fly has the longest mitogenome of all (16,360 bp), and *A. grahami*, a Calliphoridae fly, has the shortest mitogenome of all (14,903 bp). Tachinid flies have a smaller average mitogenome size (~ 15,076 bp) than the other flies in this superfamily, and the Oestridae flies have a relatively larger mitogenome (~ 16,031 bp). We observed that the size of the total PCGs, tRNAs, and rRNAs are well-maintained across this superfamily, with an average length of 11,145 bp, 1482 bp, and 2113 bp, respectively (Fig. 1A**,** green, yellow, and blue line, Table 1).

The difference in mitogenome size in insects can be attributed to variations in the length of non-coding regions, especially the control region that differs in length as well as the pattern of sequences (Fig. 1B)^104,144^. In addition, based on mtDNA sequence similarity among all the Oestroidea flies, *Blepharipa* sp. has high similitude with the Tachinid Fly *E. flavipalpis* (87.83%), followed by the two hairy maggot blowflies, *Chrysomya albiceps* (85.51%) and *C. rufifacies* (85.44%). Another well-studied uzi fly, *E. sorbilans* has an 84.82% sequence similarity with *Blepharipa* sp., while Gasterophilus horse botfly has the lowest sequence similarity (~ 77%) with *Blepharipa* sp. (Supplementary Data 3A).

#### Gene content and arrangement

We found that the Oestroidea mitogenome represents the reserved gene arrangement of Ecdysozoan, for which it can be easily distinguishable from other bilaterians (Lophotrochozoa and Deuterostomia)^140^. The mitogenome of *Blepharipa* sp. and other Oestroidea have three core tRNA clusters, including (1) *trnI-trnQ-trnM,* (2) *trnW-trnC-trnY* and (3) *trnA-trnR-trnN-trnS1-trnE-trnF,* as depicted in Figs. 1C and 2. A comparative study revealed that the Oestroidea superfamily has 4 different kinds of mitogenome arrangements (Fig. 1C). The majority of the Oestroidea flies (25 out of 36) in this study have ancestral (A) dipteran type mitogenome sequences (Table 1)^145^. However, there are some minor inconsistencies exist in the Calliphoridae family (blowflies), such as the insertion of extra tRNAs (*trnI* in the genus Chrysomya and *trnV* in *D. hominis*) or the translocation of tRNA (*trnS1* in *C. chinghaiensis*) (Fig. 1C)^21,24^. Barring this, all organisms, including *Blepharipa* sp., follow a standard dipteran gene arrangement and have 37 genes in their respective mitogenomes (insertion of tRNA into the genus Chrysomya and *D. hominis* raises gene count) (Fig. 1C (i)(ii), Table 1). In the case of dipterans other than the Oestroidea superfamily, species like gall midge (Cecidomyiidae), mosquitos (Culicidae), and crane flies (Tipulidae) exhibit various rearrangements in mitochondrial tRNAs, such as the absence, inversion, translocation, and extreme truncation of certain genes (Supplementary Data 1A)^146,147^.

### Non-coding regions

#### Control region (CR) of *Blepharipa* sp. and comparison with Oestroidea

**Figure 3:**
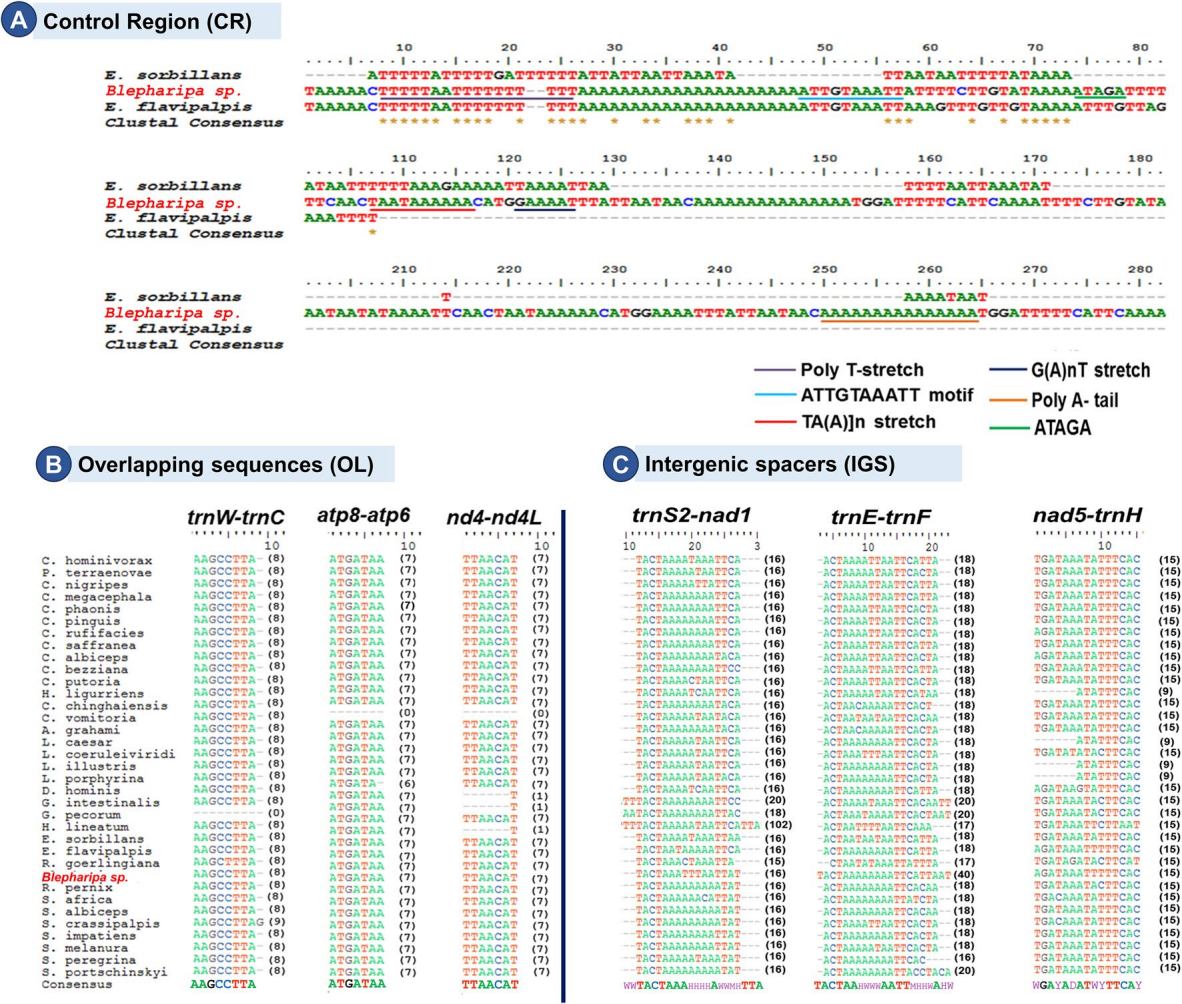
Conserved non-coding regions; (**A**) AT rich control region Alignment of *Blepharipa* sp. with other two Tachinidae species. (**B**) Three alignments of the common overlap region between *trnW-trnC*, *atp8-atp6* and *nad4-nad4l*. (**C**) Three alignment of the consensus gap region between *trnS2-nad1* (TACTAAAHHHHAWWMH), *trnE-trnF* (ACTAAHWWWAATTMHHWA), *nad5-trnH* (WGAYADATWYTTCAY) genes of all 36 Oestroidea mitogenome (where, W = A/T, H = A/T/C, Y = T/C, D = G/T/A, M = A/C).

This region in the metazoan mitogenome is a single sizeable non-coding sequence containing essential regulatory elements for transcription and replication initiation; it is therefore named the control region^148,149^. Similar to other Diptera, the CR of *Blepharipa* sp. is also flanked by *rrnS* and the *trnI-trnQ-trnM* gene cluster (Fig. 2). Sequence similarity with other Oestroidea superfamily species indicates that this segment is variable due to the lack of coding constraints^150^. The CR sequence of *Blepharipa* sp. 75.49% similar to another tachinid fly *Elodia flavipalpis,* followed by *Chrysomya bezziana* (71.15%) (Supplementary Data 3B). Despite its overall high variation in nucleotides, this region harbors multiple different types of repeats (e.g., tandem repeats, inverted repeats)^42,151^ and conserved structures namely Poly-T stretch (15 bp), [TA(A)]n-like, G(A)nT-like stretches, and poly A tail (15 bp)^152–154^(Fig. 3A). Another conserved motif, “ATTGTAAATT” we found in the CR of *Blepharipa* sp. and *E. flavipalpis* (Fig. 3A). Such conserved structures are thought to play role in the regulatory process of transcription or replication. After binding with RNA polymerase,  they keep the initiating mode of transcription or replication by preventing the transition to elongation mode without affecting its open-complex structure^155,156^.

The CR is also known as the AT-rich region for having the maximum proportion of A/T nucleotides (91.4% for *Blepharipa* sp.) than other regions of the entire mitogenome. We observed that the Tachinidae family has higher A + T content than other groups, with the highest levels in the Mulberry uzi fly, *E. sorbillans* (98.10%), and AT poor CR regions identified in *G. intestinalis* (80.80%) and *G. pecorum* (80.82%) (Oestridae)^42^ (Supplementary Data 2A). In this study, the CR of thirteen species have above 90% A + T content, and the top 3 are the tachinid flies, led by *A. grahami*, *D. hominis* and *Blepharipa* sp. consecutively. The CR is prone to high mutation, yet the substitution rate is low due to high A + T content and directional mutation pressure^144,154^. This part of the mitogenome differs significantly in length among insects, ranging from 70 bp to 13 kb, and it accounts for most of the variation in mitogenome size^153^. We noted that the CR size of 36 Oestroidea flies ranges from 89 to 1750 bp, of which 16, 12, and 8 species can be categorized as large (> 800 bp), medium (200–800 bp), and small (< 200 bp) CR respectively, and *Blepharipa sp* (168 bp) falls under the small category (see Fig. S8 in Supplementary Note). The longest non-coding control region of Oestroidea flies is found in *R. pernix* (as mentioned in "Mitogenome annotation and documentation") while the shortest CR is present at *A. grahami* that might explain its small mitogenome size which is the smallest in this superfamily (Fig. 1B). We observed that the mean GC content of < 200 bp CR is 8.84%, which is less than (medium-sized CR: 11.83% GC and large-sized CR: 12.04% GC) of the species with longer CR length (Table 1). The average GC content of Tachinid flies' CR is 6.46%, with a mean CR length, 234 bp, and two tachinids, *Exorista sorbilans*, and *Elodia flavipalpis* have 105 bp long CRs which is relatively smaller than other reported flies, and their GC contents are 1.9% and 7.9%, respectively^32^.

#### AT richness of control region and role of sequencing method

Multiple large-scale sequencing of mitogenomes from different lineages reported that D-loop or control region (CR) is extremely AT biased has a higher substitution rate (above 50%, an average of organelle genomes in 2012)^43,144,157^. Possible reasons for this would be the presence of the mitogenome in an incredibly mutagenic compartment that generates energy and has to contend with the abundance of ROS (Reactive Oxygen Species) that facilitates GC to AT mutations while providing a relatively poor DNA repair mechanism^158^. This type of locus with extreme base compositions is responsible for technical glitches in Illumina and other massively parallel sequencing systems that led to low quality and under-representation of these regions despite the generation of vast amounts of data^159,160^. Sequence coverage bias can be introduced at different stages from library preparation to sequencing and assembly (e.g., high cluster densities on the Illumina flow-cell suppress GC-poor reads; changes of sequencing kits, protocols, and instruments; bias can also differ between labs, runs, and also lane to lane within the same flow-cell)^161,162^. According to numerous sources, PCR amplification during library preparation is the primary cause of the under-coverage of GC-extreme regions in high-throughput sequencing (HTS) methods (e.g., Illumina) for sequencing mitogenomes^161,163^. Also, some hidden factors in the protocol, particularly the thermocycler and temperature ramp rate, can influence GC content dependent coverage bias^161^. A study even reported that local GC content could influence relative coverage by different HTS (e.g., Illumina, PacBio) among the various individual genomic windows^164^. This bias is suggested to be mainly introduced due to the formation of secondary structures in single-stranded DNA. This subsequently leads to the issue of low-coverage of AT-rich sequence regions, e.g., a study reported that genomic regions with 30% GC content had tenfold less coverage than sequences with 50% GC content^164^. In contrast, the PCR-free PacBio workflow provides more uniform coverage of the genome and doesn't rely on GC content^164,165^. To address these difficulties, strategies free of PCR amplification have been developed and shown to have exellent coverage of AT-rich genomes (*Plasmodium falciparum*) but are still not widely adopted commercially ^166^. Therefore, it is likely that the < 10% GC content at the CR of the newly sequenced mitogenome of *Blepharipa* sp. (GC: 7.3% at CR; CR length: 168 bp) obtained via the NextSeq Illumina Platform was inadequate to retrieve its full-length. In particular, the published mitogenomes of two other tachinid flies (*E. sorbilans* GC: 1.9% at CR, CR length: 105 bp and *E. flavipalpis* GC: 7.6% at CR, CR length: 105 bp) that have very short CR lengths and are extremely GC-poor in nature may be victims of the low coverage issue^32,42^.

#### Impact of repeats on different sequencing technologies and assembly method

There is a major difference in the natural abundance of repeats in different species, which complicates sequencing and assembly procedures and the implementation of adequate algorithms^167,168^. High-throughput sequencing (HTS) technologies are rapidly emerging, and many forms of technologies are currently in use, each with its distinct aspects that determine its ability to distinguish between different types of repeats. The most widely used technique is the Illumina Sequencing method, owing to its lower error rate (< 0.1%) in sequencing, except for substitution errors^159,169^.

Most second-generation sequencers provide short-read data; for example, Illumina's sequencing by synthesis routinely generates read lengths of 75–100 base pairs (bp) from libraries with insert sizes of 200–500 bp, hindering assembly of longer repeats and duplications^168,170^. The issues regarding short read length might be overcome by using PacBio or Nanopore but they have high single-pass error rates (11–15% for PacBio and similar for Nanopore)^171–175^. PacBio's improvisation for high-throughput HiFi reads can produce assemblies with considerably fewer errors at the level of single nucleotides and small insertions and deletions. In contrast, Nanopore-generated ultralong reads up to 2 Mb can improve contiguity and prevent assembly errors caused by long repeated regions^176^. Sequencing systems such as Roche/454 pyrosequencing technologies can deliver reads up to 1000 bp, but have difficulty with precisely sequencing homopolymers, leading to indel errors in these regions^177^. All the sequence data generated should be optimized for PacBio or Nanopore high-coverage, long-range sequencing, with some Illumina data for error correction. However, Illumina's short reads are affordable, reliable, and can solve most aspects of any genome, including some coding regions, damaged transposable elements, and tandem repeats, making Illumina robust for genome sequencing^167^.

The assembly techniques are sensitive to repetitive stretches, which can cause ‘breakage' of a continuous assembly and collapse, where the number of copies of repeats found in a genome assembly is less than the real number^167^. Typically, a genome is assembled using one of two methods. The first is the ‘*de Bruijn graph*', which is utilized by second-generation sequencing data (e.g., Illumina) to avoid the pairwise overlap step on a large number of short reads in input^178–181^. This technique employs subsequences (*k*-mers) that must be longer than the entire repeat region (which is usually between 21 and 96, with 31 being the default option), else all repeats would collapse (e.g., ALLPATHS-LG)^167,182^. In comparison to other sequence assemblers, SPAdes constructs contigs using many *de Bruijn graphs* to reduce assembly errors while making full use of a range of *k*-mers of varying lengths to produce more complete assemblies^181^. Nonetheless, a few other issues related to *de Bruijn graph* obstruct the genome assembly procedure. The splitting of reads into k-mers may destroy the structure of the repetitive regions, which is detrimental to the recovery of the repetitive segments^183^. The frequency of *k*-mers obtained from reads with many repeats are often much higher than the regular coverage of sequencing, but those with few repetitions may fail to meet the basic coverage criteria, making assembly tough to obtain^183^. The *de Bruijn*-based assemblers use cutoff criteria to prune out low coverage regions, which reduces the complexity and makes the algorithms viable, but it has an inevitable consequence on the final assembly's effective length and genome coverage ^184^. Thus, uneven sequencing depth impedes assembly as *de Bruijn graph* uses the read depth information for constructing contigs and scaffolds^185^. Second, ‘*overlap/layout/consensus (OLC) methods*’ for third-generation sequencing data are primarily utilized by overlap graphs to store prefix-suffix overlaps between the long (noisy) reads in input^186,187^. Because the overlap step compares each read to all other reads, there is a larger computing requirement than with the *de Bruijn* technique. Unlike the *de Bruijn* method, the OLC method is not restricted by any *k*-mer size and may resolve repeats that are shorter than the read length. Prior to the emergence of longer reads such as PacBio and Nanopore, shorter Illumina reads were regularly assembled using the *de Bruijn* method since OLC could be computationally intensive^167^.

In general, mitogenome's CR, including *Blepharipa* sp., contains a variety of tandem repeats, inverted repeats, and duplications^42,168^. Altogether, it remains possible that the short reads of the Illumina sequencer, along with the limitations of *de Bruijn graph*-based assemblers, might result in the control region collapsing and the sequence mis-assembling. Coverage is still a critical issue affecting the CR region since the length of the CR is longer than the read length and it is rich in tandem repeats, which is a common problem that current genome assemblers struggle to fully and reliably assemble.

#### Overlapping sequence (OL) and intergenic spacer (IGS) regions

The overlapping sequences (OL) and intergenic spacers (IGS) are widely reported in the mitogenome of Diptera, with a variety of sizes and spots occurring during evolution^104^. We found 10 overlapping sequences in the Muga uzi fly mitogenome, with the longest 8 bp OL spanning over *trnW* and *trnC* genes (Fig. 2). Two other major OLs are located over the juncture of *atp8-atp6* (ATGATAA), and *nad4-nad4l* (ATTATAA) found in *Blepharipa* sp., both are in same length (7 bp) and common in the insect phylum because of the direct adjacency of the genes^188,189^. Unlike other species *C. chinganensis, D. hominis, G. intestinalis H. lineatum* do not form OL over *nad4*-*nad4l* genes. Including that *C. vomitoria* have no OL region with the genes *atp8*-*atp6* and *nad4*-*nad4l* while *trnW* and *trnC* do not overlap in the *G. pecorum* mitogenome (Fig. 3B, Supplementary Data 4A,B). We noticed that thirty types of OLs are present over different gene boundaries in mitogenomes of 36 Oestroid flies, and the quantity of OLs ranges from 4 (*C. vomitoria*) to 21 (*S. crassipalpis*). The total size of OL varies from 16 bp in *C. vomitoria* to 102 bp in *D. hominis* (Supplementary Data 4A)*.* A close look at the mitogenome arrangement reveals that *D. hominis* encountered the insertion of *trnV,* which led to the formation of an overlapping region (64 bp) between the *trnK-trnD* cluster (Fig. 1C (iv)).

The mitogenome of *Blepharipa* sp. has 15 IGSs, which are spread across PCGs, tRNAs, and rRNAs. It has only one major IGS of over 20 bp, the 40 bp IGS1, located between *trnE* and *trnF*. In addition, 3 medium-sized IGSs (> 10 bp) are present in this mitogenome, namely, IGS2 (*trnS1*-*trnE,* 19 bp), IGS3 (*trnS2-nad1*, 16 bp), and IGS4 (*nad5-trnH,* 15 bp). The remaining 11 IGSs have a length of less than 10 bp. Several dipteran insects have the 5 bp conserved motif (ATCWW) at IGS1 and the 7 bp conserved motif (TWYTTMA) at IGS4 to a lesser extent. In addition, IGS3 has been reported to contain a 7 bp consensus motif (ATACTAA) across Lepidoptera and a 5 bp (TACTA) motif conserved across Coleoptera^190,191^. Our comparative study exhibits a variation of IGSs across the Oestroidea superfamily in terms of length, positions, and numbers of occurrences. Within 36 gene boundaries of 36 Oestroidea flies, 29 distinct IGSs have been found, with quantities ranging from 9 in *S. crassipalpis* to 18 in *L. coeruleiviridis* (Supplementary Data 4C). Only five IGSs (*trnL2-cox2*, *cox3-trnG*, *trnE-trnF*, *nad5-trnH*, *trnS2-nad1*) are found in all members of the Oestroidea. The average length of these 5 IGS regions is 5.30 bp, 6.22 bp, 18.63 bp, 14.33 bp, and 18.52 bp, respectively. Moreover, 4 of them form conserved motifs in this superfamily, namely *trnE-trnF* (ACTAAHWWWAATTMHHWA), *nad5-trnH* (WGAYADATWYTTCAY), *trnS2-nad1* (TACTAAAHHHHAWWMH), and *cox3-trnG* (HTAAYT). These motifs are also found in the similar location of other Diptera mitogenomes (Fig. 3C, Supplementary Data 4D)^98^. The *trnS2-nad1* spacer is a common feature of insect mtDNAs and is considered to comprise the binding site for DmTFF, the bidirectional transcription termination factor^192,193^. We found 7 such rare IGSs that occur only in any one Oestroidea fly; these are *nad2-trnW, trnK-trnD, trnD-atp8, trnN-trnE, trnH-nad4, trnT-trnP, and trnV-rrnS* (Supplementary Data 4C). *H. lineatum*'s IGS at *trnS2-nad1* is 102 bp long, making it the longest IGS in this superfamily’s mitogenome, and this species also has the largest proportion of nucleotides in its spacer region (174 bp). This study found many species with a total IGS length of over 100 bp, including 11 Calliphoridae flies (out of 19), 3 Oestridae flies (out of 4), 4 Sarcophagidae flies (out of 9), and 2 Tachinidae flies (out of 4) (Supplementary Data 4C). We also identified a unique 70 bp long spacer region located between *trnN* and *trnE* of *C. chinghaiensis* owing to translocation of the *trnS1* gene (Fig. 1C (iii)) (Supplementary Data 4C).

### Coding regions

#### Nucleotide composition and comparison

To quantify A + T content and AT/GC skewness, the nucleotide composition of various regions in the mitogenome of *Blepharipa* sp. has been determined. The *Blepharipa* sp. mtDNA has T = 38.8%, C = 12.9%, A = 40.0%, and G = 8.7%, with a total A + T content of 78.4%. These measures are close to another uzi fly species, *Exorista sorbillans* (T = 38.4%, C = 12.6%, A = 40.0%, G = 8.9%, A + T = 78.4%)^42^. This  species' concatenated PCGs, tRNAs, and rRNAs consist of 77.32%, 78.24%, and 82.54% of A + T content, respectively. The longest non-coding area with the highest A + T content  among all genomic regions is the control region, with 91.4% of A and T combined. The positive (+ve) AT skew values obtained for the whole mitogenome, concatenated PCGs, tRNA, rRNAs, and CR are 0.021, 0.022, 0.025, 0.014, and 0.198, respectively, confirming the existence of more adenine than thymine in this organism^194^. The four PCGs from the N strand of the *Blepharipa* sp. mitogenome have a higher proportion of AT (79.1%) than the nine PCGs from the J strand (76.3%). Except for the smallest PCG, *atp8*,  all PCGs show −ve (negative) AT skewness regardless of strands. In the case of tRNA, three tRNAs from both strands show −ve AT skew. The AT content of eight N-strand tRNAs are found to be higher (80.11%) than that of fourteen J-strand tRNAs (77.18%) (Fig. 2, Supplementary Data 1A). While similar to other dipteran mitogenomes, the rRNAs are transcribed on the N-strand, with only rrnS (small rRNA) exhibiting −ve AT skew^195^. The intergenic spacer sequences (excluding the control region) are also AT biased, with 89.20% A+T content.

**Figure 4:**
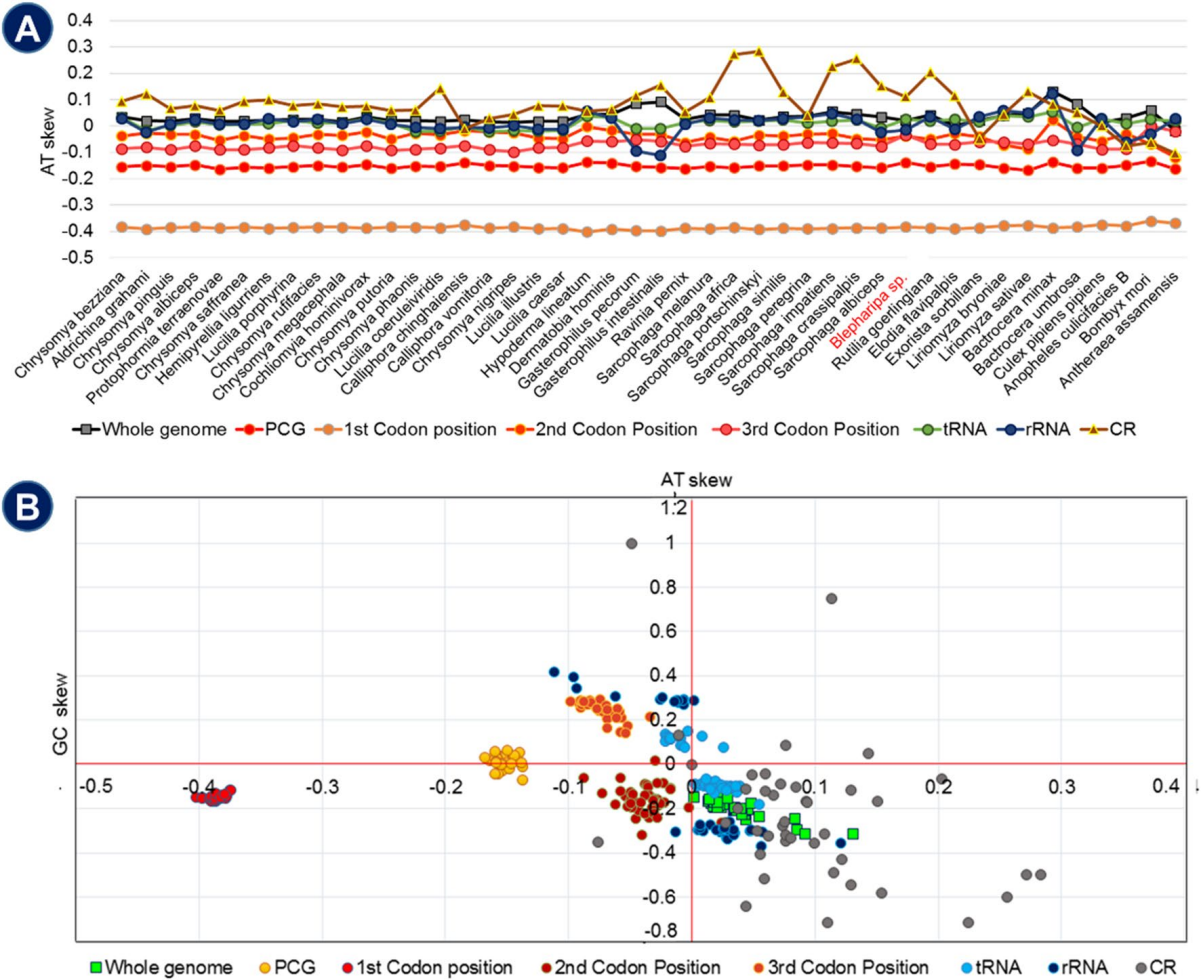
Nucleotide skew plot; (**A**) Trend of AT skew across the Oestroidea superfamily and outgroups. (**B**) AT skew vs GC skew of different genetic position of 44 organisms (CR shows maximum variation and 1st codon position shows least variation).

We observed that in this superfamily, Tachinidae flies (*E. flavipalpis:* 79.96%, *E. sorbillans*: 78.44%, and *Blepharipa* sp.: 78.41%) have a significantly AT-biased mitogenome, as found by Zhao et al. in the *E. flavipalpis* mitogenome^32^. We also found that the mtDNA of  *Blepharipa* sp.  has a +ve AT (0.021) skew and a –ve GC (− 0.194) skew, which is similarly observed in Oestroidea flies, indicating that the flies in this study have more As and Cs than Ts and Gs (Supplementary Data 2A). This study shows that he J strand (9 PCGs, 14 tRNAs) of all Oestroidea species is T/C skewed, whereas the N strand (4 PCGs, 8 tRNAs, omitting rRNAs) is T/G skewed and violated the Chargaff's second parity rule, implying asymmetric replication of the genes (Fig. 4B**,** Supplementary Data 2A)^194,196^. Overall, the AT content of the N strand is more than that of J strand genes (Supplementary Data 2A).

The Tachinid fly, *E. flavipalpis,* has the highest A + T content (79.09%) in its PCGs, followed by the uzi flies, *E. sorbillans* (77.64%) and *Blepharipa* sp. (77.28%) (Table 1). However, the nucleotide bias in individual PCGs has moved towards higher use of Thymine rather than Adenine, and this trend is observed in Diptera and Oestroidea fly PCGs (Supplementary Data 2A). The J strand PCGs and N strand PCGs show that both the gene sets are moderately T skewed (−ve AT skew); while the J strand gene set is moderately C skewed, N strand is firmly G skewed and, a similar kind of pattern is also observed in other insects (Supplementary Data 2A)^190^. The fourfold degenerate codons do not influence amino acid selection. Whereas, twofold degenerate codons are restricted to change their 3rd position for the presence of twofold redundant codon positions. Codon redundancy arises due to change in a nucleotide in 2nd codon position accounts for sixfold codon degeneracy^194^. We calculated A + T/G + C content and skew for all codon positions of Oestroidea flies (Fig. 4). We found that 3rd codon positions are rich in A + T content (Highest mean in *nad4l*: 92.85 ± 4.17%, lowest mean in *cytb*: 88.08 ± 4.59%; n = 36) than other positions (1st codon position AT%: highest mean in *atp8*: 79.18 ± 4.18%, lowest mean in *cox1*: 57.45 ± 1.55%; 2nd codon position AT%: highest mean in *nad6*: 75.63 ± 1.37%, lowest mean in *cox1*: 59.57 ± 0.41%). In the case of Tachinidae (n = 4), A + T content in different codon positions are (3rd codon position AT%: highest mean in *nad3*: 94.87 ± 2.1%, lowest mean in *atp8*: 91.67 ± 5.73%; 1st codon position AT%: highest mean in *atp8*: 86.12 ± 2.28%, lowest mean in *cox1*: 58.84 ± 1.09%; 2nd codon position AT%: highest mean in *nad4l*: 60.45 ± 0.41%, lowest mean in *cox1*: 59.57 ± 0.41%) enriched with higher A + T content than other species and so the 3rd codon positions (Supplementary Data 2B) and has also documented by other research^197^. It is also clear that the standard deviation of the 2nd codon position is quite low, whereas the standard deviation of the 3rd codon position is the highest among other codon positions. This may reflect the prevalent fourfold degeneracy of codons and the frequency of codon usage variation in different species. The PCGs have the most conserved AT and GC skewness in the sample set, eventually forming four distinct clusters for complete PCGs, 1st, 2nd, and 3rd codon positions. We found that the presence of lowest AT skew (−ve) at the 1st codon position, that is consistent across and beyond the Oestroidea superfamily (Fig. 4B). We also identified that the abundance of Ts and Cs (Pyrimidine) is higher in the 1st and 2nd codon positions than As and Gs (Purine) respectively, and the 3rd codon location shows the abundance of As over Ts and Cs over Gs. (Supplementary Data 2A). This observation appears to apply to all Oestroidea flies. According to the GC skew analysis, the –ve GC skew value is reasonably consistent with other dipteran insects, except for a few lower Diptera^198^.

In the case of tRNAs and rRNAs, Tachinid flies have a high proportion of A + T content. Also, being part of the N-strand, the average proportion of rRNA A + T content of the Oestroidea superfamily is 79.98%, which is greater than the average A + T content of tRNA (76.5%). The mean tRNA and rRNA A + T content of 4 Tachinid flies are 77.98% and 82.40%, respectively (Supplementary Data 2A). For RNAs, the maximum of the flies shows a very small +ve AT skew at their concatenated tRNA, rRNA genes except for a few species from Calliphoridae, *S. albiceps, G. intestinalis,* and *G. pecorum*, etc. (Supplementary Data 2A). The Gasterophilus genus of the Oestridae family shows the lowest A + T content, whereas the other two species of the Tachinidae family contain the highest A + T content among the superfamily. The Control region shows +ve AT skewness for 34/36 species, and it varies enormously in Oestridae, Sarcophagidae, and Tachinidae family, whereas *S. portschinskyi* represents the highest +ve AT skew and *E. sorbillans* shows the lowest −ve AT skew at CR region. These two species belong to Sarcophagidae and Tachinidae families, respectively (Fig. 4A, Supplementary Data 2A).

#### Synonymous codon usage pattern

The synonymous codons of protein-coding genes code for similar amino acids that do not appear at an equal frequency^199,200^. Differences in synonymous codon usage bias are present in a wide range of organisms, from prokaryotes to unicellular and multicellular eukaryotes^201–203^. Since diverse genomes possess typical patterns of synonymous codon usage, thus the comparative codon usage analysis facilitates the understanding of the evolution and adaptation of living organisms^204,205^. Mitochondrial genomes are considered as an evolutionary paradox with a relatively conserved gene content and small size. The genetic code of mitochondria differs from the standard genetic code^206^. We know that codon usage pattern deviates during evolution, but how is still entirely not known. Here, an extensive comparative study has been described to decipher the pattern of codon usage in the Oestroidea flies mitogenome including six other Diptera species and two Lepidoptera moths.

**Figure 5:**
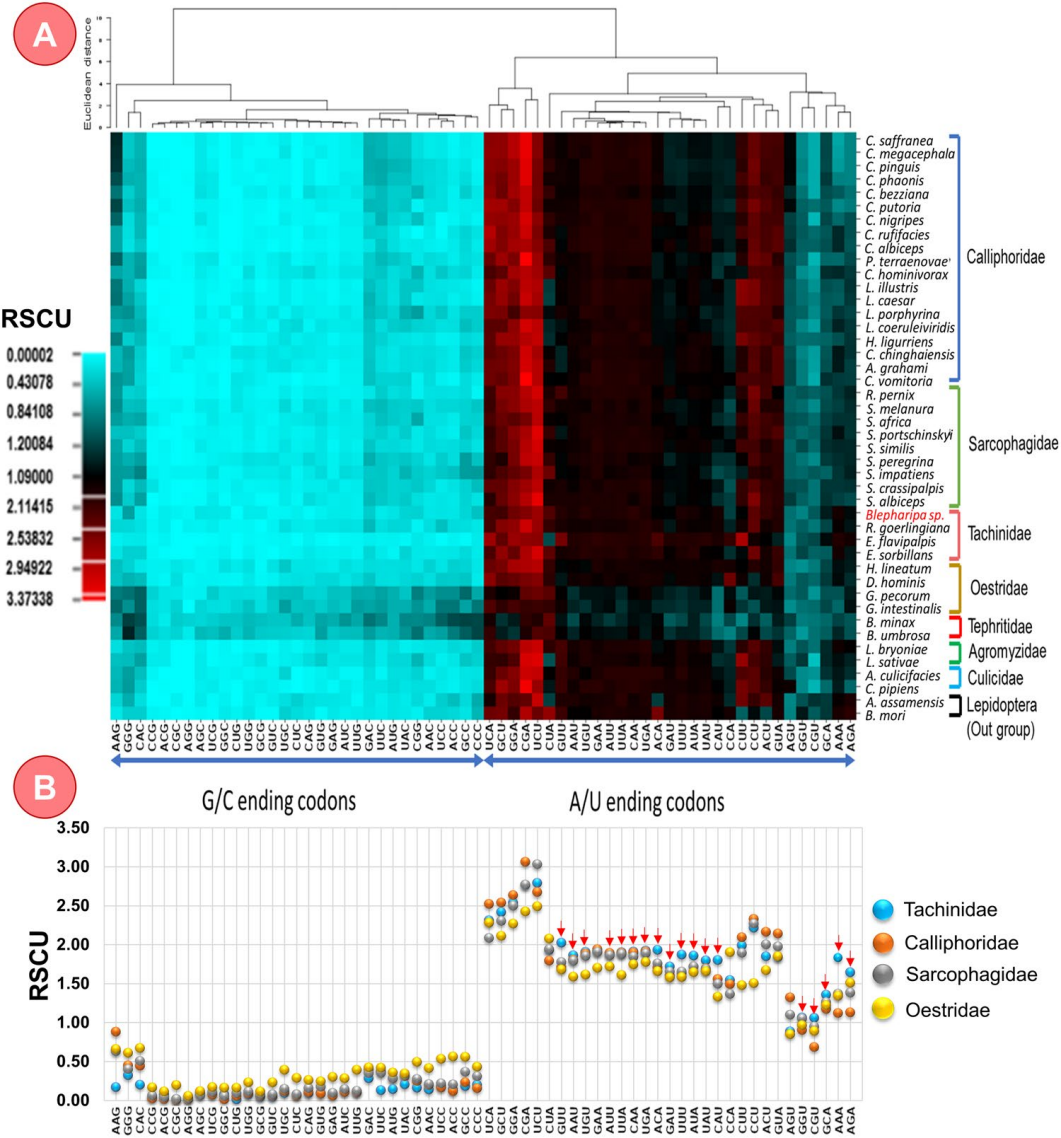
Variation of Relative synonymous codon usage (RSCU) in different species and families; (**A**) RSCU Cluster analysis of 36 species from Oestroidea Superfamily, 6 organisms from other Diptera and 2 organisms from out group (Lepidoptera). Termination codons are excluded. The heat-map was drawn with CIMminer. Bigger RSCU values, suggesting more frequent codon usage, are represented with darker shades of red. (**B**) Mean RSCU of different families of Oestroidea superfamily, higher RSCU of tachinids denoted by red arrow.

The *Blepharipa* sp. mitogenome contains all of the typical mitochondrial 13 PCGs and is constituted with a total of 3722 codons. The total number of available codons in the mitogenome of Oestroidea varies from 3699 codons in *L. caesar* to 3730 codons in *C. chinghaiensis* (Supplementary Data 5D). Similar to *Blepharipa* sp., *nad5* is the largest PCG present in Oestroidea mitogenome containing ~ 573 codons, and *atp8* is the smallest PCG with ~ 55 codons (Supplementary Data 5D). All PCGs are spread over both the strands of the double-stranded mitogenome, where 9 PCGs in the J strand and 4 PCGs in the N strand contain different quantities of codons and it is similar to Oestroidea flies as well (see Fig. S9 in Supplementary Note). The *Blepharipa* sp. PCGs are heavily biased towards A/T ending codons, accounting for around 92.07% of available sense codons. In comparison to Oestroidea species, codons carrying A or T at the third position (AT3) have a strong preference, ranging from 75.41% in Gasterophilinae subfamily member *G. intestinalis* (another member *G. pecorum*: 76.9%) to 97.31% in *E. flavipalpis* (Fig. 5, Supplementary Data 5B). The G ending CUG (Leu) is absent from our sequenced organism's mitochondrial PCGs, with the T or U ending UCU (Ser) being the most frequent codon (4.40%, RSCU = 2.73). PCGs of the Sarcophagidae family exclusively use UCU (Ser) as most frequent codon. The Oestridae family uses UCA in addition to UCU for coding of same amino acid. Except for *H. ligurriens* (UCU), the Calliphoridae family employs CGA as the most common codon for coding Arginine. The use of most recurring codons in Tachinid mitochondrial PCGs varies; both Uziflies employ UCU, but *R. goerlingiana* and *E. flavipalpis* use CGA and CUU for Arginine and Leucine coding, respectively (Supplementary Data 5A). Noticeably, the most frequent codons always ended by A or T nucleotide, and a clear distinction between the A/U and G/C ending codons have been found from the cluster analysis of all sense codons, although there are some variations in the RSCU of different organisms always persist (Fig. 5A). This analysis reveals that related species preserve the stability of codon usage behavior; as the use of one particular codon increases, the use of other synonymous codons decreases, implying a larger bias in occurrence. For instance, Lysine (K) is encoded by AAA and AAG in insect mitochondrion. The Tachinidae flies choose the AAA codon, but other Oestroidea flies prefer the AAG codon for coding the same amino acid (Supplementary Data 5A). Moreover, the Tachinidae family has eighteen A/U ending codons (GUU, ACA, UGA, CAA, UGU, UUA, AUU, UUU, AAU, AUA, AAA, CAU, UAU, GAU, AGA, GCA, GGU, CGU) that have a more significant codon usage than other families, with seven of them consisting entirely of A/U nucleotides (Fig. 5B, Supplementary Data 5E). Thus, similar to other invertebrate species, the individual RSCU evaluation of all thirteen PCGs reveals a general tendency toward codons with A or U at 3rd place^104^.

**Figure 6:**
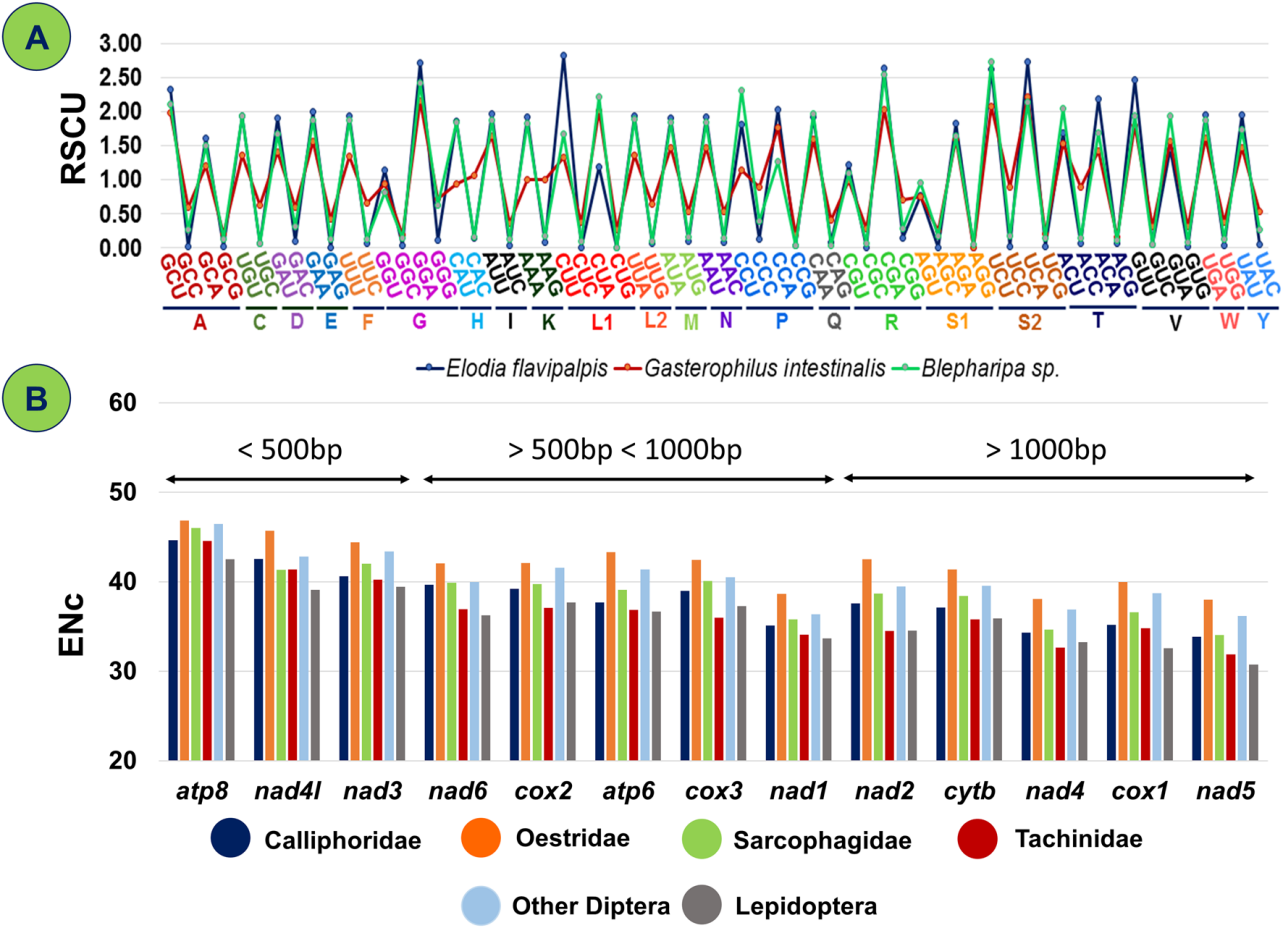
(**A**) RSCU value comparison between *E. flavipalpis* (Maximum A/U at 3rd codon position), *G. intestinalis* (Minimum A/U at 3rd codon position) and *Blepharipa* sp. (**B**) Average Effective codon number (ENc) of 13 PCGs of different families of Oestroidea flies and out groups.

To explore more trends of codon usage in each gene, we measured effective number of codons (ENc) in all PCGs of our test species. The ENc values range from 20 (just one codon allocated to each codon family), which indicates extreme codon bias, to 61(equal usage of all synonymous codons), which indicates no codon bias^114^. In mitochondrial context, every PCG is essential, and in the absence of adequate evidence on gene expression, ENc plays a valuable role in determining codon bias^207^. Analysis shows ENc values of each PCGs (n = 13 * 36) of Oestroidea flies varied from 30.11 (*nad5* gene of *E. sorbilans,* strong bias) to 49.19 (*atp8* gene of *G. intestinalis,* weak bias). If the ENc value of any gene is closer to 20, it implies that the gene has an extreme codon bias, and many studies have shown that ENc < 35 indicates a mostly high codon bias^114,115^. On the other hand, if the ENc value of any gene nearer to 61 denotes extremely weak bias, so we believe that ENc > 45 should denote relatively weak codon bias. The family-wise mean ENc value of mitochondrial PCGs is depicted in Fig. 6B. The most biased gene observed in this superfamily is *nad5* (Mean ENc: 34.15 ± 2.36), followed by *nad4* (Mean ENc: 34.62 ± 2.12), *nad1* (Mean ENc: 35.56 ± 1.79), and *cox1* (Mean ENc: 36.02 ± 2.31) with relatively strong codon bias for every family except families like Oestridae of Oestroidea superfamily and Tephritidae (Fig. 6B). The least biased gene is *atp8* (Mean ENc: 45.20 ± 1.96), followed by *nad3* (Mean ENc: 41.34 ± 2.02) and *nad4l* (Mean ENc: 42.45 ± 1.85) genes of every family of Oestroidea including Tachinidae family exhibit relatively weak codon bias (ENc > 40). Including that, the mean ENc values of each mitochondrial PCGs of Tachinidae flies exhibit relatively lower ENc values than other Oestroidea flies, that implies that the mitogenome of this family possesses stronger codon bias (Fig. 6B).

#### Relation between nucleotide composition and codon usage

The nucleotide composition has a strong correlation with codon usage in the Oestroidea superfamily as well as other dipteran mitochondria (see Fig. S10 in Supplementary Note). The Tachinidae family exhibits lower mean ENc values across the PCGs, indicating a greater codon bias at the gene level (Fig. 6B). As evidenced by RSCU analyses, all 13 PCGs are skewed toward A/T, resulting in codon usage biases (Fig. 5). Correlation among 3rd codon position and relative synonymous codon usage value pointed out that total the RSCU value of the codons with A/U at 3rd codon position is inversely proportional to the GC3 content and directly proportional to the total codon usage value when G/C at 3rd codon position (p < 0.001) (see Fig. S11 in Supplementary Note). For example, *G. intestinalis*, a horse botfly shown in orange color, has the highest GC3 content and has less biased codon usage among the PCGs. *E. flavipalpis* have the lowest GC3 content among the Oestroidea flies and display relatively stronger codon bias and low ENc value (Fig. 6A). Similarly, *Blepharipa* sp. mitogenome also shows very less GC3 content and has a comparatively stronger codon bias (Fig. 6A). The Pearson correlation results reveal that ENc has a significant positive correlation with the GC content at 3rd codon positions of PCGs (GC3, R = 0.374, p < 0.01 and GC3s, R = 0.374, p < 0.01), and on the other hand other codon positions, particularly GC1 and GC2, have a weak but significant negative correlation with ENc (GC1, R = − 0.121, p < 0.01 and GC2, R = − 0.112, p < 0.01) (Supplementary Data 6D). This indicates that by increasing GC content at 3rd codon position the ENc values of the genes also increase and as a consequence codon usage bias will decrease in Oestrodea mitogenome since insect mitochondrial genomes are rich in AT content (Fig. 5)^32,42^.

#### ENc-plot for determining the factors of codon usage bias

**Figure 7:**
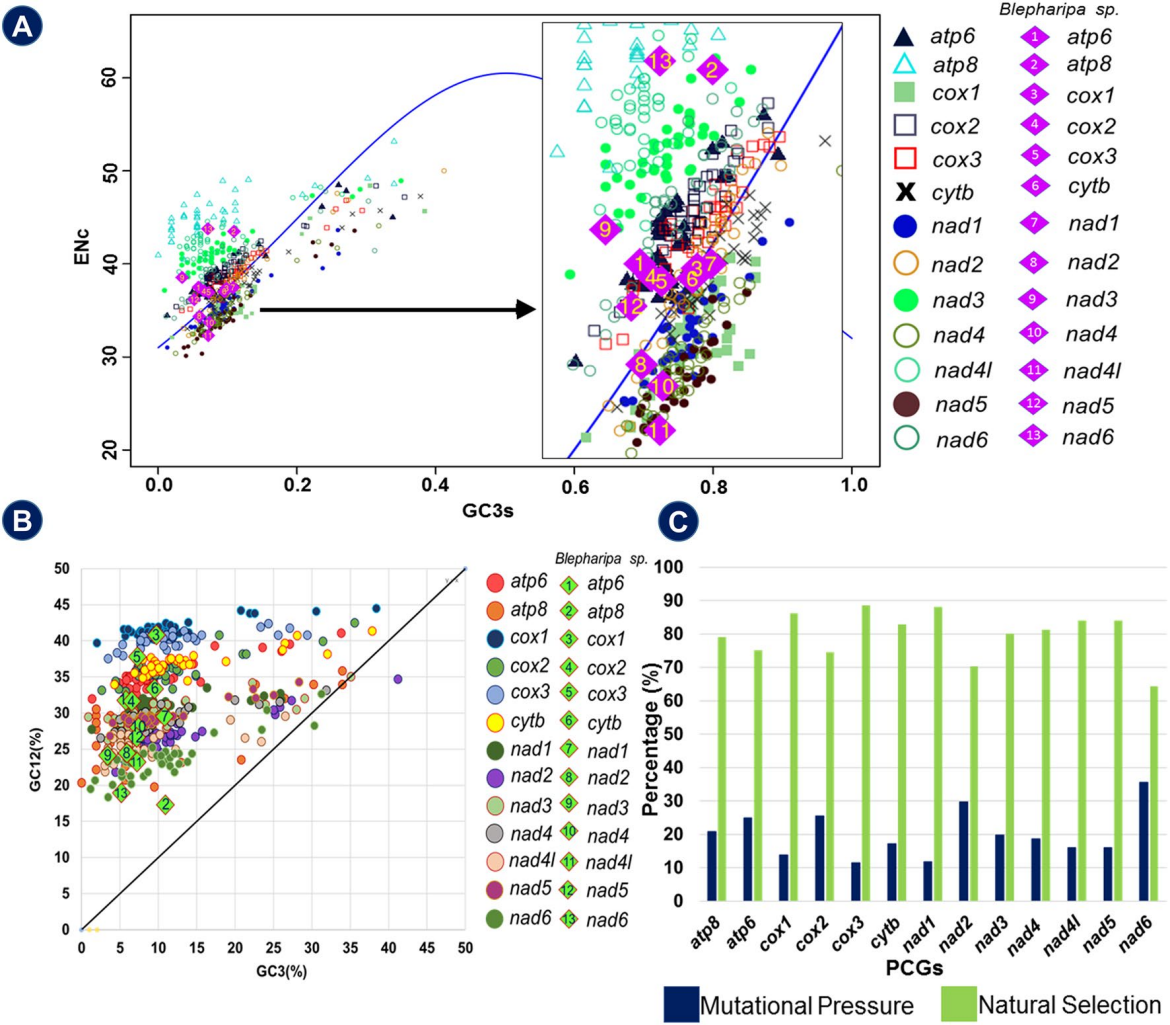
(**A**) The ENc vs. GC3s plots of Oestroidean mitochondrial protein-coding genes. The standard curve ENc = 2 + GC3s + 29/[GC3s2 + (1 − GC3s)2] represents the expected ENc to GC3s. (**B**) Neutrality plots (GC12 vs. GC3) of 13 PCGs of 42 species. GC12 stands for the average value of GC content in the first and second position of the codons (GC1 and GC2). While GC3 refers to the GC content in the third codon position (each dot signifying a gene). (**C**) Probability of selection pressure on each PCGs of Oestroidea. The regression line of all PCGs denoted by y = mx + c (Where, Mutational Pressure (M) = m * 100, Natural Selection (N) = 100 − M).

 To better understand nucleotide composition and codon usage bias, ENc values are plotted against the GC3s values in ENc-plot, where the standard curve demonstrates the functional relationship between ENc and GC3s is under mutation pressure rather than selection^116^. The plot suggests that if the codon usage bias depends entirely on GC3s, all of the points would be precisely on the standard curve (corresponding to the ENc values)^116,120^. As a result of this plot, most of its points do not lie close to the standard curve, indicating that the role of GC3s in mutation bias is not the key factor in codon bias (Fig. 7A). The ENc-plot depicts that some points lie on or near the curve (on or above: *atp6, cox2, cox3*, *nad6*; both sides of the curve: *nad2;* and on or below the curve: *cox1, cytb, nad1, nad4, nad5*), while others are far away (above the curve: *atp8, nad3*, *nad4l*) indicating variation in codon usage bias and their causes. Whereas the positions of *Blepharipa* sp. PCGs in the plot are like: *cox1, cytb, nad1*, and *nad2* are closer to the curve; *atp6, cox2, cox3, and nad6* are slightly above the curve; *nad4, and nad4l* are below the curve, and *atp8, nad3*, and *nad6* located much above the curve. Therefore, this outcome suggests that along with mutation pressure for shaping codon usage bias in different species, some independent factors, like natural selection strongly influence the bias pattern and these factors are more dominant than mutation pressure^208^.

#### Neutrality test for determining the factors of codon usage bias

The neutrality test has been carried out to measure the degree of directional mutation pressure against selection in the codon usage bias of mitogenome. As the ENc-GC3s plot could not estimate precisely which of mutation pressure or natural selection is more essential^117,120^. According to the theory, nucleotide heterogeneity is the effect of bidirectional mutation pressures between G/C and A/T pairs, and this pressure induces directional changes more in neutral parts than in functionally significant parts^117,209^. Here in this analysis (GC12 vs. GC3), regression slopes of 13 PCGs substantially deviated from the diagonal line (regression coefficient < 1; lowest 0.1149 (*cox3*) to highest 0.3563 (*nad6*)) by contributing a significant but weakly positive correlation (R^2^ < 0.9; P-values < 0.01) between observed GC12 and GC3 (Fig. 7B, Supplementary Data 6B). The plot suggests that relative neutrality of GC12 varies from 11.5% in *cox3* to 35.6% in *nad6* as compared to GC3 (100% neutrality or 0% constraint) in the mitogenome of Oestroidea superfamily^119^. It also indicates that the intensity of mutation pressure is weakest in *cox3*, accounting for only 11.5%, and the highest in *nad6,* accounting for 35.6% towards neutrality. We observed in this study that the low and narrow distribution GC content of Oestroidea varies from 20.03% to 29.83% in WMG and 20.9% to 32.1% in PCGs, and it has never exceeded 50% of the total nucleotide content of any species. The variation and scarcity of GC content in the 3rd position of codon (e.g., GC3 of *cox3*: 3.43–29.38% and of *nad6:* 1.72–30.28%) and narrow distribution of GC12 content (e.g., GC12 of *cox3*: 37.59–41.41% and GC12 of *nad6:* 18.4–28.28%) also observed (Fig. 7C, Supplementary Data 6A). It has been reported in earlier studies that selection against mutational bias can cause a narrow distribution of GC content and a poor correlation between GC12 and GC3^210,211^. The predominance of natural selection and other factors accounted for almost 88.5% in *cox3* (highest) and 64.3% (lowest) in *nad6* relative constraint*.* Thus, the mitogenome of the Oestroidea superfamily retains a low and restricted distribution of GC contents owing to the selection against mutation bias^116,211^.

As the Oestroidea mitogenomes are highly AT-rich (highest for *E. flavipalpis*, WMG: 79.96%, PCG: 79.06%; lowest for *G. intestinalis*, WMG: 70.16%, PCG: 67.88%), the prevalence of A/T ending codons (highest for *E. flavipalpis*, 3rd position: 72.72%, lowest for *G. intestinalis*, 3rd position: 63.41%) has been observed (Supplementary Data 2A). Therefore, this is in line with the theory that the strong bias of the Oestroidea mitogenome's codon usage towards a large representation of NNA and NNT codons is due to mutational bias towards A/T, which was also documented for other mitochondrial genomes^116,210,212^.

#### Relation between gene length and codon usage

Longer genes need more energy to improve accuracy by selecting favourable codons that can minimize the proofreading costs and maximize the rate and accuracy of translation^33,213^. This study shows that the smallest gene (*atp8*, mean length: 161.75 bp) has the highest mean ENc (45.20) and the longest gene (*nad5*, mean length: 1719.16 bp) has the lowest mean ENc (34.15) (Fig. 6B) among the thirteen mitochondrial PCGs of Oestroidea. The Pearson correlation statistics show a satisfactory and significant negative correlation of ENc with gene length (R =  − 0.742, p < 0.001) (Supplementary Data 6D). It indicates that the length of mitochondrial genes in Oestroidea flies is inversely related to the effective number of codons (ENc), which ensures that as gene length increases, ENc reduces and, as a result, codon usage bias increases. Thus, longer mitochondrial genes show stronger codon bias than smaller genes. This trend has also been found while studying *B. mori* mitogenome, and it was further mentioned that mitochondrial gene length and codon usage bias related to their expression level^116^. It has been widely documented that highly biased codons are mainly observed in highly expressed genes, and mitochondrial longer genes are also highly expressed^33,116,213^. Our findings are in accord with previous studies in which prokaryotes like *E. coli* and *Yersinia pestis* exhibit a common trend of elevated codon usage bias for longer genes, unlike nuclear genes of multicellular eukaryotes namely Yeast and *Drosophila*, where smaller genes appear to be more biased than longer genes^33,116,213^.

### Phylogenetic inference

#### Phylogenetic relation of Oestroidea superfamily

**Figure 8:**
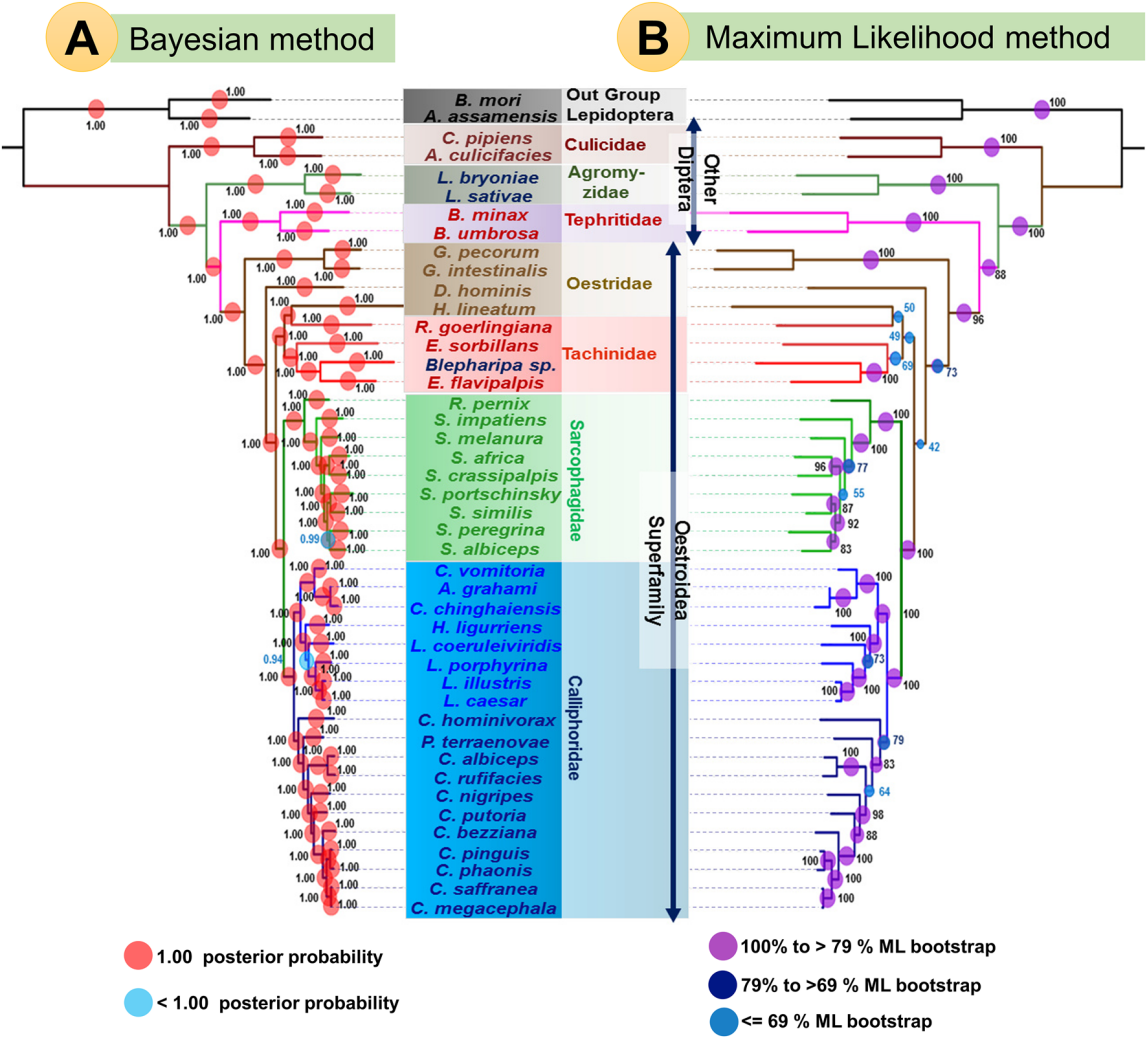
(**A**) Phylogenetic tree inferred from nucleotide sequences of 13 PCGs of 44 organisms (36: Oestridea superfamily, 6: other Diptera and 2: Out group Lepidoptera) using maximum likelihood (ML) method in RaxML 8.2.x (5000 bootstrap replicates). (**B**) Phylogenetic tree inferred from nucleotide sequences of 13 PCGs of 44 organisms (36: Oestridea superfamily, 6: other Diptera and 2: Out group Lepidoptera using Bayesian inference (BI) method in MrBayes v3.2.6.

The phylogenetic relationship found through 13 mitochondrial protein-coding genes represents a similar topology in both Bayesian Inference (BI) and Maximum Likelihood (ML) methods. It established a link among major clades with very good support from Bayesian posterior probability and moderate bootstrap support from ML analysis. Adjacent grouping of *Blepharipa* sp. and *E. flavipalpis* with 100 percent bootstrap support and congruent support from Bayesian posterior probability (1.00) is evident within the monophyletic clade of *E. sorbilans* (BI/ML: 1.00/69) (Fig. 8A,B). This study revealed that the two families namely, Sarcophagidae (1.00/100), and Calliphoridae (1.00/100) belong to the monophyletic group of the Oestroidea superfamily. The Calliphoridae family is distributed in two different clades, wherein a single clade *Chrysomya* sp. along with *P. terraenovae* (1.00/79), separated from other Calliphoridae flies (1.00/100) as found by other research as well^214^. While the Oestridae and Tachinidae families could not recover as monophyletic, they have formed a paraphyletic relationship with the rest of the Oestroidea flies. Though taxonomically *H. lineatum* belongs to the Oestridae family, our inference using both methods exhibits polyphyletic relation with Oestridae flies and clusters with *R. goerlingiana* of Tachinidae with 50% bootstrap support^215^. Therefore, with the exception of Calliphoridae our analysis establishes the monophyletic status of the Sarcophagidae, and Oestridae is shown as the sister group of remaining Oestroidea flies via both ML and BI methods^214^. Both Lepidoptera sequences group together and are represented as outgroup for this analysis.

#### Location of Tachinidae at Oestroidea phylogeny

The relationship between the different oestroid lineages  remains a controversy in Dipteran phylogeny.  In earlier studies, the speciation of Oestroidea and closely related lineages have been linked with higher diversification rates ^216^. This has made it hard to resolve relationships among these taxa, particularly concerning the origin of the Tachinidae family. According to the morphological and molecular evidence, nearly every other family of Oestroidea has been assigned as a potential sister clade of Tachinidae^32,191–195^. As per the common nature of the internal parasitism of the arthropods and subscutellum development, some poorly defined families (e.g. Rhinophoridae (not in this study)) have also been proposed as a sister group of tachinids^217,218^. In any case this is less convincing  as in reality certain representatives of Calliphoridae, Sarcophagidae, and Oestridae have sclerotized subscutellum^219^. Some sarcophagids are parasitoids of insects and other arthropods, while certain calliphorids are parasitoids of snails and earthworms^218^. However, greater diversity of feeding habits and breeding environments, including hematophagous parasitism of birds and mammals has been evident from these groups of species^216,218^.

Tachinidae is the morphologically most heterogeneous subgroup of this superfamily, lacking clear morphological synapomorphy and usually serving as a dumping place for taxa with confusing characteristics^220^. In one morphological study Tachinidae has been presented as polyphyletic,  while the bulk of their subfamily exist as paraphyletic^221^. Many taxa in the Oestroidea superfamily share morphological or molecular characteristics, and their placement in the tree indicates that the Sarcophagidae and Calliphoridae are currently monophyletic. However, we have not been able to demonstrate that the Tachinidae and Oestridae are monophyletic. This discordance from conventional knowledge may be attributed to long branching of two genera and insufficient Oestridae and Tachinidae taxa sampling. In other ways, this issue may indicate that these families are likely to have seen significant variation in molecular and morphological traits, contributing to exceptionally developed parasitic behaviour and making  it challenging to compare with the conventional characters^214^.

All the flies included in this study exhibit parasitism in diverse forms.  The Oestridae family parasitizes mammals and the Tachinidae family parasitizes insects (Table 1). The phylogenetic inference reveals very little about the monophyly of Oestridae and Tachinidae using the combined 13 mitochondrial genes, yet having substantial support from bootstrap and posterior probability may be due to phylogenetic inertia playing a major role in resolving true relationship. According to the physical law of inertia, a moving body subjected to various forces will move in the direction of ‘least resistance.' The biological world obeys the same rule of inertia as the inorganic world, with evolutionary lineages following the path of least resistance, implying that evolution will continue in the direction of previously acquired adaptations despite environmental perturbations^222–224^. This is well illutrated by the failure of birds to evolve viviparity^225^, high altitude behavior in a valley population of a South American rodent despite half a million years of isolation^226^. In this scenario, we can say that parasitism might have evolved before the formation of families like Oestridae or Tachinidae, and persistence of common characters or traits among species hinders distinguishing the phylogenetic relationship.

#### Nonsynonymous substitution

The PAML package has been used in this study to investigate whether the PCGs had undergone any beneficial adaptations. Two different trees (gene tree and species tree) have been used to estimate nonsynonymous to synonymous rate ratios (ω = Ka/Ks or dN/dS) in all PCGs through the maximum-likelihood method.  The positive selection is defined as dN/dS > 1 ,  neutrality is defined as dN/dS = 1 , and  negative selection is defined as dN/dS < 1 . First, a very simple model known as the one-ratio model (M0) has been used, it allows a single ω ratio for all branches. The ω ratios that we estimated from 13 individual PCGs are all less than 1 for both the trees, facilitating enough support for the occurrence of negative selection acting on the mitochondrial genes. In this study, the gene *atp8* shows the highest ω value (gene tree: 0.11541, species tree: 0.12904), and *cox1* shows the lowest ω value (gene tree: 0.02328, species tree: 0.02035) among the 13 mitochondrial PCGs (see Supplementary Tables S1–S13 online). To retain the important mitochondrial functions in energy metabolism, strong purifying selection plays an important role in the evolution of the mitogenome of Oestroidea flies.

Since insect endo parasitism was acquired only in tachinid flies thus, we assumed that there may have been some evolutionary pressure on this lineage. Therefore, the lineage belongs to *Blepharipa* sp. of the Tachinidae family with other members (if available in the same clade) considered as foreground lineage for branch specific two ratio model in two different trees. The two-ratio model using gene tree showed except *cytb* (ω0 = 0.03005, ω1 = 0.00010), ω for the other 12 genes on the foreground branch (ω1) is greater than the background lineages (ω0) but not more than 1. The gene *nad5* (ω0 = 0.04559, ω1 = 0.92099) and *atp8* (ω0 = 0.11541, ω1 = 0.93957) have maximum ω value for the lineage of interest in the foreground branch. Through the reference species tree, the *nad2* gene (ω0 = 0.08155, ω1 = 0.04346) exhibits low ω value at the foreground branch than background branches and *nad4* (ω0 = 0.04972, ω1 = 0.20965) and *atp8* (ω0 = 0.05145, ω1 = 0.20860) show maximum ω1 value. The log-likelihood difference, 2ΔlnL = 2(l_1_-l_0_), between the one-ratio and two-ratio models presents that the two-ratio model fits better than the one-ratio model. Using the gene tree, we obtained a maximum 2ΔlnL from the *nad5* gene (2ΔlnL = 27.01) with a significant level 0.001 < p and df = 1 and a minimum from the *atp8* gene (2ΔlnL = 0.00007199), which is comparatively very less significant (p < 0.995) than other genes. In the case of the species tree, we got a maximum of 2Δl from the *nad6* gene (2ΔlnL = 1560) with a significance level of 0.001 < p and df = 1 (see Supplementary Tables S1–S13 online). Overall, in each tree's foreground or background branches, the ω ratio never exceeded 1, considering the fact that the branch leading to the uzi flies' common ancestor has gained more nonsynonymous mutations than synonymous mutations, and therefore putting more selective pressure on it than other branches. However, the possibility of relaxed selection cannot be excluded, and thus, the assessment does not support positive selection on the foreground branch. Positive selection normally operates on a few sites for a brief amount of evolutionary time, but the signal for positive selection is usually drowned out by the continuous negative selection that occurs on the majority of sites in a gene sequence^227^. Thus, the branch leading to the common ancestor of uziflies (Tachinidae) have seldom nonsynonymous mutations, indicating that most have been occupied by purifying selection.

Purifying selection cannot generate better genes; it is only responsible for preserving the function of a gene^227^. The mitochondrial protein products are crucial for survival; thus, their activities are more restricted. Hence, it can be inferred that the numerous selection constraints present in codons effect their evolution through influencing transcription and translation efficiency^212^.

#### Regression model fitting between substitution rates and codon usage indices

**Figure 9:**
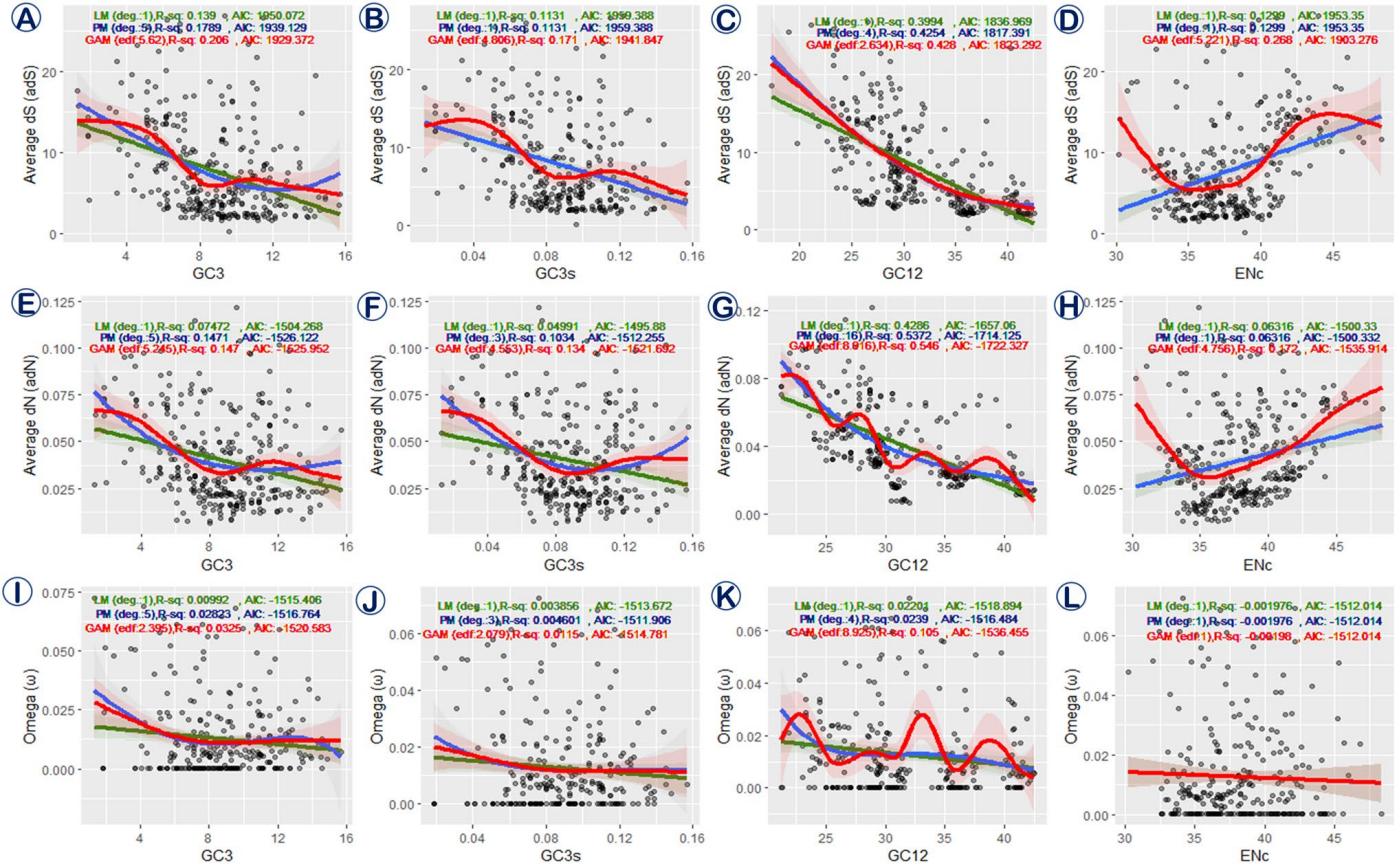
Univariate regression model fitting between response variables, divergence rate (dS, dN and ω) and predictor variables, codon usage indices (GC3, GC3s, GC12, ENc) of training datasets; (**A**–**D**): average synonymous divergence (adS) rate vs codon usage indices; (**E**–**H**): average nonsynonymous divergence rate (adN) vs codon usage indices; (**I**–**L**): omega ratio (ω) vs codon usage indices; Green: Linear Model (LM), Blue: Polynomial Model (PM), Red: Generalized Additive Model (GAM), light colour represent the 95% confidence interval; R-sq (R^2^): Coefficient of determination; AIC: Akaike information criterion; deg: degree, edf: effective degrees of freedom.

In this analysis, data from three datasets with three response variables (dS, dN, and ω) and four predictors (GC3, GC3s, GC12, and ENc) have been used to fit three regression models, namely linear model (LM), polynomial model (PM), and generalized additive model (GAM) with training dataset (75%) after removal of few extreme outliers. The test dataset (25% of raw data) has been used for the prediction of RMSE (Root Mean Square Error) and R^2^ (Coefficient of determination). All univariate regression models from the different compositions of each variables group are shown in Fig. 9 and documented in Supplementary Data 8. The degree of predictor variables in all linear models is  1, whereas the estimated optimal degree of predictor variables in the polynomial model has different in all response variables except ENc, where AIC and BIC criteria selected linear function. The linear function (one degree) has been selected for predictor GC3s against dS, while the polynomial function with the maximum degree (16 degrees) has been chosen for GC12 versus dN (Fig. 9B,G). We note that both polynomial and gam() selected a linear function of ENc for the response variable ω (Fig. 9L). In the GAM, higher values of edf (effective degrees of freedom)  have been fitted than the degree of polynomial models for GC3, GC3s, and ENc against dS and dN, while against ω, only GC12 has been selected for higher edf in GAM than the chosen degree in PM. The accuracy of different models (Adjusted R^2^: Coefficient of determination) shows GAMs fit better than any other model in the training dataset, whereas in the test dataset, R^2^ has been better predicted by PM in dS vs GC3, ω vs GC3s and GC12, and in LM of dN vs GC3s. In the training dataset, GAM shows lower residual standard error for the response variables like dS (vs GC3, GC3s, ENc) and dN (vs GC3s, GC12 ENc) except for the predictors GC12 and GC3 respectively. Whereas, against response variable ω, PM shows smaller residual standard error for all predictor variables. In the test dataset, the predicted RMSE (Root Mean Square Error) of GAM in all response variables against ENc is low, and also GC3s, GC3, and GC12 against dS, ω, and dN respectively show low RMSE. All models show the same RMSE for ENc against the response variable ω. In PM, predictors like GC3s, GC12, and ENc have low RMSE versus ω, while in LM, GC3 versus dS and dN as well as GC3s versus dN have low RMSE. The estimated AIC values of models display that GAMs have the lowest value, excluding the GC12 against dS and GC3 against dN where PM shows the lowest AIC (Supplementary Data 8).

Model fitting is preliminary for performing regression analysis since this is based on specific assumptions such as linearity, homoscedasticity, independence, and normality^228^. Therefore, some diagnostics for regression analysis are required to evaluate the model assumptions and identify whether any data has a significant, unexpected impact on the analysis. In this case, we performed two diagnostic analyses: residuals vs fitted (R-F) values to test the assumption of linearity and homoscedasticity and quantile–quantile (Q-Q) plots to assess residual normality^228,229^. We also checked for homoscedasticity using the Breusch Pagan test and normalcy using the Shapiro–Wilk test^230,231^. The R-F plots of three regression models indicated that the spread of residuals is randomly distributed, except for the predictor GC12 versus dS and dN, where the spread is not constant but varies along with the fitted values. Furthermore, the distribution of residuals does not appear to be persistent for the response variable ω. The residuals of GAM roughly form a "horizontal band" (red line) around the 0 lines. It implies that the variances of the error terms are almost equal in GAM (Fig. S12). The Breusch Pagan test with LM and PM supports that the fitted response with GC12 is not homoscedastic (homo = similar, scedasticity = spread) against dS and dN (p <  < 0.01).   In contrast, against ω,  the distribution of residuals with GC3, GC3s is heteroscedastic and with GC12 and ENc residuals are poorly homoscedastic (0.1 < p > 0.01) (Supplementary Data 8). The residuals Q-Q plot indicates that none of the regression models are normally distributed since the data do not lie entirely on the straight line and deviate at the left and right edges (Fig. S13). The Shapiro–Wilk test for normality also exhibits that all the fitted models significantly deviate from a normal distribution (p <  < 0.05) (Supplementary Data 8). When the distribution of the error terms is skewed and the variance of the error terms is not constant, a transformation of the response variable may be quite helpful^232^.

**Figure 10:**
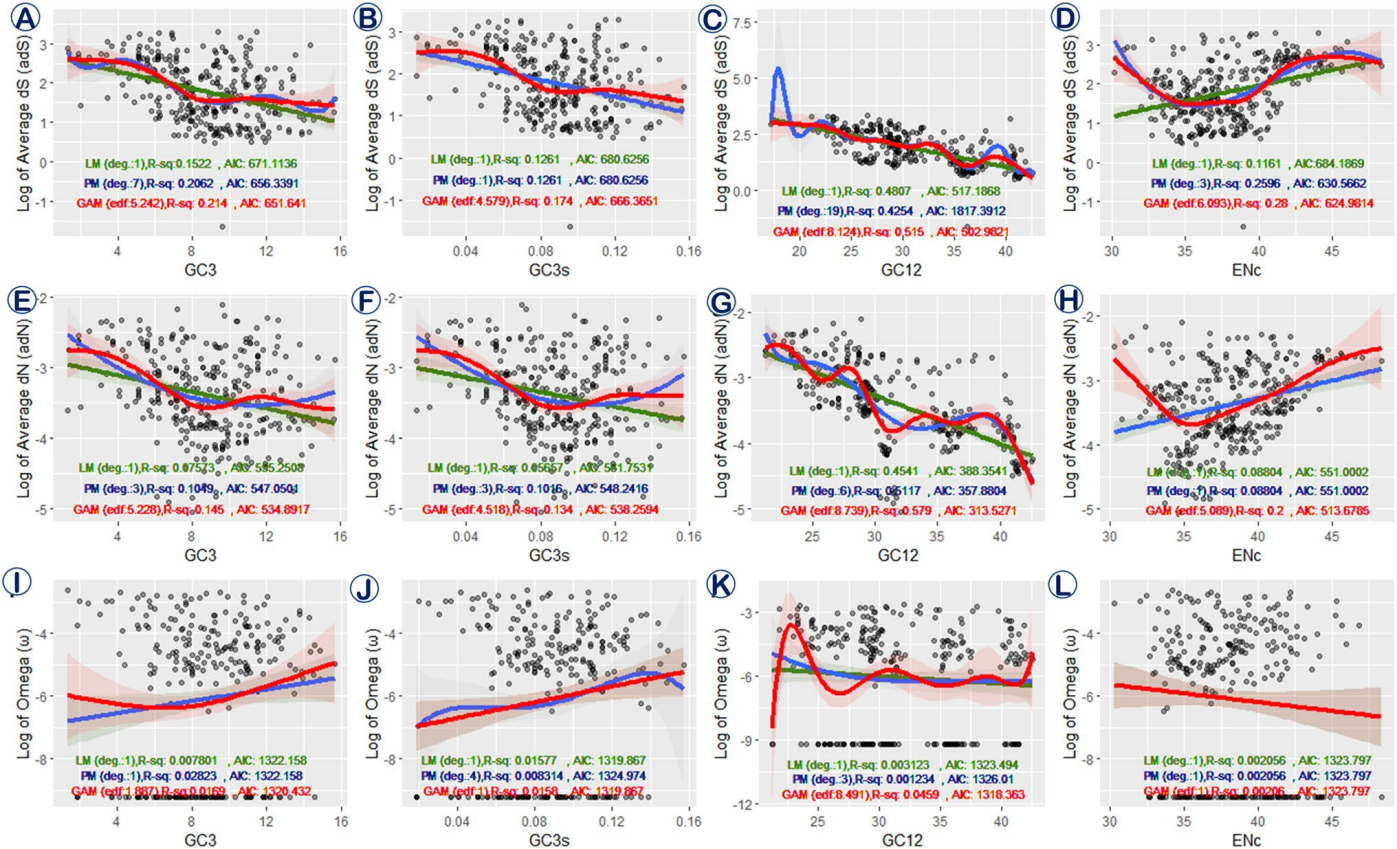
Univariate regression model fitting between logarithmic response variables, divergence rate (dS, dN and ω) and predictor variables, codon usage indices (GC3, GC3s, GC12, ENc) of training datasets; (**A**–**D**): log of average synonymous divergence (adS) rate vs codon usage indices; (**E**–**H**): log of average nonsynonymous divergence rate (adN) vs codon usage indices; (**I**–**L**): log of omega ratio (ω) vs codon usage indices; Green: Linear Model (LM), Blue: Polynomial Model (PM), Red: Generalized Additive Model (GAM), light colour represent the 95% confidence interval; R-sq (R^2^): Coefficient of determination; AIC: Akaike information criterion; deg: degree, edf: effective degrees of freedom.

We transformed the response variables using logarithms (log(adS), log(adN), and log(omega)) and fitted aforementioned three regression models (LM, PM, GAM). All univariate regression models of log transformed response variables are shown in Fig. 10. The outcome shows that the difference in the accuracy of model fitting (R^2^) is not significant between normal and logarithmic response variables, but log(adS) fits better than other response variables for all four predictors (Figs. 9, 10, Supplementary Data 8). GAM is similarly suited better than LM and PM in this case, as evidenced by higher R^2^ and lower AIC values. However, for predictor ENc, all three models chose a linear relationship for the ω log response, resulting in the same R^2^ and AIC values, as well as for the predictor GC3s, GAM selected a linear relationship against log ω (Fig. 10J,L). The diagnostic R-F plot demonstrates that, after the logarithmic transformation of dS, there is a uniform spread of residuals with fitted values, except in PM for GC12 (Fig. S14). The logarithmic dN indicates a decreasing trend of residuals along with the fitted values, except for predictor variable GC12. Whereas, logarithmic ω with all predictor variables exhibits a unique pattern of residual distribution, with negative residuals forming a straight line with a declining trend in the R-F plot (Fig. S14). The Breusch Pagan test shows that for LM, homoscedasticity with GC12 increases and decreases for other predictor variables, and PM homoscedasticity with GC12 increases for logarithmic dN and ω (Supplementary Data 8). The predictor ENc shows declining homoscedasticity for dN after the logarithmic transformation. The Q-Q plot indicates that the residual distribution is approaching normality after the logarithmic transformation of dS and dN as data points fall on the straight line (Fig. S15). Whereas, after the logarithmic transformation of ω, it shows a bimodal distribution of residuals for all predictor variables. The Shapiro–Wilk test shows that after the transformation of dN, the null hypothesis cannot be rejected except for GC12 and GAM shows the almost normal residual distribution for predictor variables GC3 and GC3s (Supplementary Data 8). Overall, non-linear models, especially the generalised additive model, fit variables better than linear models since the number of outliers and degree of scattering were maximum around the fitted line of the linear models^233^.

#### Correlation between nucleotide substitution rates and codon usage indices

If all synonymous sites remained subjected to the same selective pressure, deviation from absolute neutrality would have no effect on the efficacy of dS (rate of synonymous divergence) in the analysis of genes for relative divergence times, mutation rates, or non-synonymous evolutionary rates^234^ (Supplementary Data 7). However, the intensity of selection differs widely around synonymous sites, which is frequently owing to the fitness penalty for differences in inaccurate translations across sites and variation in the optimal rate of gene expression across genomes^202,235–237^. Whereas the ω ratio tests the direction and degree of selection on the adaptation of amino acids, with criteria of ω < 1, = 1, and > 1 representing negative purifying selection, neutral evolution, and positive selection, respectively. However, the simplistic use of the ω ratio to identify positive selection, by computing dN and dS between two sequences, is hardly effective, so lineage-specific branch-wise is much more useful for detecting positive selection^238^ (Supplementary Data 7).

According to this study, the average synonymous divergence rate (dS) in LM is negatively correlated to codon usage indices like GC3, GC3s, and GC12 while positively correlated to ENc, with slopes of − 0.78, − 72.27, − 0.64, and 0.63 respectively. Log transformed dS shows a similar pattern of association with the codon usage indices, with slopes of -0.10, -10.02, -0.09, and 0.07, respectively (Supplementary Data 8). It appears that increasing GC content reduces the pace of synonymous evolution, whereas increasing ENc increases it. It means the synonymous divergence rate decreases with the rising of GC content and codon usage bias.

The PM, like the LM, has a negative and linear connection with GC3s, as well as a positive and linear correlation with ENc, whereas GC3 and GC12 have a 5th and 4th degree polynomial relationship with dS (Fig. 9A–D). As per PM, the synonymous evolution rate is reduced linearly by increasing GC content at synonymous 3rd codon positions and by the reduction of ENc. On the other hand, dS decreases polynomially as GC content increases (GC3 and GC12). The log transformed dS (log(adS)) has a linear relationship with GC3s, whereas GC3, GC12, and ENc have 7th, 19th, and 3rd degree polynomial relationship with it, respectively (Fig. 10A–D). It implies that in PM, the reduction of log(adS) occurs linearly by increasing GC content at synonymous 3rd codon positions (GC3s). As GC3 increases,the log(adS) is decreases by a 7th-degree polynomial function; the Fig. 10 A shows an almost horizontal log(adS) up to 4% GC3, then decreases up to 8% GC3, and finally it is horizontal up to 12% GC3. Whereas, a 19^th^ degree polynomial relationship with GC12 may underestimate the model due to data overfitting. The 3rd-degree polynomial relationship with ENc displays a valley-shaped graph in Fig. 10 D in which log(adS) decreases up to ENc value 34, remains almost parallel to the horizontal axis until ENc value 39, and increases up to ENc value 45 (Fig. 10).

GAM exhibits edf of 5.62, 4.806, 2.634, and 5.221, respectively with GC3, GC3s, GC12, and ENc (Fig. 9A–D, Supplementary Data 8). The synonymous divergence rate, dS, declines with increasing GC3 content, in which initially horizontal up to 4% GC3, a little decline until 6% GC3, then a severe drop up to 8% GC3, then small rise up to 11% GC3, and finally decreases again. The relationship pattern of dS with GC3s is similar to that of GC3 (Fig. 9). The correlation of dS  with increasing ENc in GAM shows a valley-shape graph where initially dS decreases up to ENc value 34 and stays almost parallel with the horizontal axis until ENc value 38–39, then dS increases again up to ENc value 43–44. While log transformed dS (log(adS)) show edf of 5.242, 4.579, 8.124, and 6.093 respectively with GC3, GC3s, GC12, and ENc (Fig. 10A–D). This implies that, as the value of GC3 and GC3s increases, the log(adS) declines in a similar way to earlier (without log transformation). When compared to GC12, the log(adS) features a spiral-shaped decreasing graph around the LM. The relationship with ENc exhibits a valley-shaped graph similar to the one found before without log transformed GAM and log transformed PM, in which log(adS) falls and remains almost parallel with the horizontal axis until ENc value 34, then rises up to ENc value 45.

Similar to the synonymous divergence rate, the nonsynonymous divergence rate (dN) is inversely correlated to codon usage indices such as GC3, GC3s, and GC12 in LM and positively related to ENc with slopes of − 0.0022, − 0.18, − 0.0028, and 0.0018, respectively (Supplementary Data 8). With slopes of − 0.058, − 5.18, − 0.073, and 0.054, log transformed dN (log(adN)) exhibits a similar pattern of a relationship with the codon usage indices (Supplementary Data 8). Increasing GC content tends to slow nonsynonymous evolution, but increasing ENc accelerates it. It indicates that when GC content and codon usage bias increase, the rate of nonsynonymous divergence reduces.

Similar to LM, the PM also has a positive and linear relation with ENc, but GC3, GC3s, and GC12 have polynomial relationships with dN at the 5th, 3rd, and 16th degrees, respectively (Fig. 9E–H). As per PM, the nonsynonymous evolution rate, dN, increases linearly with the rise of ENc. On the other hand, dN declines polynomially as GC content grows (GC3, GC3s, and GC12), in which dN decreases until around 8% of GC3 and GC3s, then slightly rises around 12% of GC3 and GC3s, while dN decreases steadily along GC12. The log transformed dN  (log(adN)) exhibits a positive linear relationship with ENc and a 3rd degree polynomial correlation with GC3, GC3s, and a 6th degree polynomial correlation with GC12 (Fig. 10E–H). The regression plot between log(adN) and  GC3, GC3s and ENc reveals a substantially identical trend to that previously mentioned without the transformation of dN, in which log(adN) reduces until roughly 8% of GC3 and GC3s, then it increases slightly  around 12% of GC3 and GC3s and the log(adN) increase linearly with the rise of ENc. In contrast, a 6th-degree polynomial relationship has been observed with GC12, in which log(adN) initially decreases up to 22% GC12, until  27% GC12 it decreases nearly parallel to the horizontal axis, then it sharply drops until 33% GC12, then it increases slightly up to 39%, and it again drops further.

GAM exhibits edf of 5.245, 4.553, 8.916, and 4.756 respectively with GC3, GC3s, GC12, and ENc (Fig. 9E–H). The nonsynonymous divergence rate, dN, follows a nearly identical pattern to dS with GC3 and GC3s, where dN reduces with increasing GC3 almost horizontally up to 4% GC3, then sharply drops up to 8% GC3, then increases slightly up to GC3 content 11%, then decreases again. The relationship pattern of dS with GC3s is similar to that of GC3. In GAM, the dN declines with rising ENc, where the valley-shaped graph of dN reduces up to ENc value 35 and continues virtually parallel with the LM until it crosses the LM at about ENc value 42, at which point the dN increases again. While log transformed dN (log(adN)) shows edf of 5.228, 4.518, 8.739, and 5.089 respectively with GC3, GC3s, GC12, and ENc (Fig. 10E–H). As the value of GC3 and GC3s increases, the log(adN) declines in a similar way to earlier (without log transformation). When compared to GC12, the dN has several ups and downs in the plot, with two particularly sharp falls at 28% and 39% GC12 content. The correlation with ENc displays a valley-shaped graph similar to the one found before without log transformed GAM, in which dN decreases up to ENc value 34 and continues almost parallel with the LM until it crosses the LM at about ENc value 40, at which point the dN increases again.

This study shows, unlike dS and dN, ω display inverse correlation to all codon usage indices (e.g. GC3, GC3s, GC12, and ENc) in LM with slopes -0.0007, -0.053, -0.0005, -0.0002 respectively (Supplementary Data 8). It suggests that ω reduces very slowly as  GC content increases and codon usage bias decreases. Whereas log transformed ω positively linked with GC3 and GC3s and inversely linked with GC12 and ENc with slopes 0.09471, 12.6259, -0.0358, -0.05606 respectively (Supplementary Data 8). It implies that log transformed ω increases with the increase of GC3 and GC3s while it declines with the increase of GC12 and ENc.

The PM, like the LM, has a positive and linear correlation with ENc, whereas GC3, GC3s, and GC12 have 5th, 3rd, and 4th degree polynomial relationship with ω, respectively (Fig. 9I–L). According to PM, when ENc increases, the ratio of nonsynonymous evolution rate to synonymous evolution rate increases linearly. On the other hand, when GC content increases (GC3, GC3s, and GC12), ω declines polynomially, where it decreases until around 6% GC3, then remains steady up to 13% GC3, before marginally decreasing again. In the case of GC3s, ω decreases up to 6% GC3s then it stays almost parallel to the horizontal axis. When compared with GC12, ω drops up to 25% of the GC12 level, then it very slowly, almost parallelly declines with the increase of GC12. In PM, the log transformed ω exhibits a positive linear relationship with GC3 and a negative linear relationship with ENc and 4th and 3rd degree polynomial correlation with GC3s and GC12, respectively (Fig. 10 I–L). The log transformed ω increases with the increase of GC3 and decreases with the increase of ENc. In comparison with GC3s, the log transformed ω increases with a spiral around the LM and a little horizontal around  8% GC3s. When compared to GC12, the log of ω decreases up to 25–26% GC12, then it becomes nearly parallel with the horizontal axis.

GAM provides edf of 2.395, 2.079, 8.925, and 1 for GC3, GC3s, GC12, and ENc, respectively (Supplementary Data 8). When correlated with GC3, ω follows a nearly similar pattern to PM, where it reduces until around 6% GC3 level, then maintains a stable parallel to the horizontal axis, and the relationship with GC3s is almost identical to that of GC3. The correlation with GC12, ω shows a highly fluctuating relationship. In contrast, with ENc, like LM, ω has a decreasing linear relationship but is almost parallel to the horizontal axis. After log transformation, the edf values are 1.887, 1, 8.491, and 1 with GC3, GC3s, GC12, and ENc, respectively (Supplementary Data 8). It suggests that GC3s and ENc are linearly associated with log transformed ω, in which GC3s is positively related and ENc is negatively related, and both are equivalent to LM. The correlation with GC3 exhibits a curve-like plot, initially declining until GC3 content is around 8% and then increasing. The GC12 again shows a highly fluctuating relation with log transformed ω.

The independent and uneven distribution of codon usage indices of mitochondrial genes across species complicates formal analysis using standard statistical linear models. Simpler linear correlations are generally employed with minimal consideration for assumption violations and do not offer estimates of the magnitude of change, instead of focusing on whether or not there is a linear or monotonic trend^239^. Alternative techniques have been expanded to allow for more complicated nonlinear trends by having response variables rely on predictor polynomials. The fully parametric model has some flaws, most notably poor fitting or overfitting of the data and the behaviour of the fitted trend at the beginning and end of the observed series^240,241^. Whereas GAMs employ automated smoothness selection methods to establish the complexity of the fitted trend objectively, and they allow for potentially intricate, non-linear trends and adequate accounting of model uncertainty^239^.

#### Accuracy assessment and pattern of best-fit models

The residuals vs observed response variables (dS, dN, and ω) plot depict the regression models' tendency for overestimation and underestimation, with high positive residual values (on the y-axis) indicating very low predictions and high negative values indicating overly high predictions^233^. The plot of residuals vs observed (R-O) values from the first case without response variable transformation reveals a significant, linear, and growing correlation between the residuals (on the y-axis) and the observed values of the dependent variable (on the x-axis). But the R-O plots of dS-GC12, dS-ENc, and dN-GC12 form a hockey-stick-like line with a curve at the lower end below the 0 line of the residual (Fig. S16). Here, the R-O plots suggest that with the increment of observed response variables, the residuals also increase. However, regardless of all models, a large proportion of the data points are closer to (above, on, or below) the 0 line of residual, which is in the ranges of roughly > 5 to < 15 of dS, > 0.025 to < 0.075 of dN, and > 0.005 to < 0.02 of ω for predictors like GC3 and GC3s, although the range somewhat differs for GC12 and ENc (Fig. S16). It implies that values of observed response variables lower than this range would be influenced by overestimation, and higher observed values are susceptible to underestimation^233^. On the other hand, after log transformation of the response variable, the R-O plots exhibit curves that are much more slanted, non-linear, and almost parallel to the 0 lines of the residual. It suggests that, as the observed response variables (dS, dN, and ω) increase, the residuals do not increase as much as they did previously when the response variables had not transformed. However, the residuals of PM for log(dS)-GC12 show a hockey-stick-like curve, whereas the residuals of LM and GAM lie over the 0 lines of the residual (Fig. S17). Therefore, the R-O plot suggests that after the log transformation of the response variables, the model's underestimation has been greatly reduced.

Univariate models based on GAMs fit better compared to linear regression and polynomial regression models where the coefficient of determination R^2^ always performed better, except for a few cases where R^2^ of LM and PM is equivalent to GAM. Other model evaluation factors, such as RMSE, Residual standard error, and AIC, are mostly attributed to improved GAM fit. The synonymous divergence rate, dS, fits better for predictor variables such as GC3, GC3s, and ENc, but the nonsynonymous divergence rate, dN fits better for GC12. This trend is observed in all regression models, and it is similar even after the logarithmic transformation of the response variable.

In general, the 3rd position of fourfold degenerate codons acts as a silent site or synonymous site where a change in nucleotides does not change the resultant amino acids. The codon usage indices like GC3, GC3s, denote GC content at the 3rd codon positions of all codons and fourfold degenerate codons respectively. The GC3, and GC3s, according to GAM,  are negatively and non-linearly correlated with divergence rates at silent sites (dS). It means that the reducing synonymous divergence rate, dS, and increasing amount of GC content at 3rd codon positions are not uniform across mitochondrial genes of various species. GAM also depicts that nonsynonymous divergence rate (dN) declines with increasing GC content at 3rd codon positions like dS. However, the model fitting of dN with GC3 and GC3s is inferior to dS. After log transformation of dS and dN, the relationship curve with ENc seems nearly similar. The same is true for GC3 and GC3s, indicating that the relationship pattern with those codon usage indices does not change after  the nucleotide substitution rate transformation. The GC12 represents the average GC content of 1st and 2nd codon positions, typically regarded as nonsynonymous sites where nucleotide alterations influence the amino acid composition. According to GAM, both the synonymous and nonsynonymous divergence rates, dS and dN, reduce as GC12 grows. The dN, however, has a wiggle in its pattern, despite fitting GC12 better than dS. The log transformed dN displays a decreasing but wiggly relationship with GC12, whereas the log transformed dS shows a decreasing trend with  a little wiggle at higher GC12. It suggests that when GC12 increases, both synonymous and nonsynonymous divergence rates drop, but synonymous divergence rate declines more smoothly than the nonsynonymous divergence rate.

The ω does not fit well as compared to dS and dN but like dN, it fits better for GC12 than other predictor variables. In addition, model fitting improved after log transformation of dS and dN, but model fitting degraded after log transformation of ω (Supplementary Data 8). The nucleotide substitution ratio at nonsynonymous and synonymous sites is defined as dN/dS or ω, and certain genes display a very low nonsynonymous substitution rate than the corresponding synonymous substitution rate, resulting in a very small ω (0.0001) for those genes. That makes a separation of data by extremely small and substantially larger values of ω, and following log transformation, such data creates a bimodal distribution, as evidenced  from the Q-Q plot (Fig. S15i–l). As a result, the data distribution deviated from normality and eventually worsened the model fitting.

Overall, synonymous and nonsynonymous divergence rates drop with increasing GC3, and GC3s with a significant decline at 4–8% GC content at the 3rd codon position, and the synonymous divergence rate is considerably higher than the nonsynonymous divergence rate (Fig. 9, Supplementary Data 7). Both dS and dN create curves that are almost opposite of the S-curve with GC3 and GC3s. Since mitochondrial genes and their 3rd codon positions are strongly AT biased, divergence rates might increase with rising AT concentration at 3rd codon positions. Although the relationship would not be linear, it will follow an S-shaped curve, with a spike in divergence rate occurring at genes with 92–96% AT content at 3rd codon positions. The ENc designates the effective number of codons of any gene; when ENc increases, codon usage bias decreases. The GAM of both dS and dN depicts a valley shape curve, implying that the rate of nucleotide change at synonymous and nonsynonymous sites initially declines, remains steady, and then slowly increases as ENc increases. It indicates that when codon usage bias reduces, synonymous and nonsynonymous substitution rates drop drastically at first, then stabilize for a time before gradually increasing.

### Codon usage bias and parasitism

**Figure 11:**
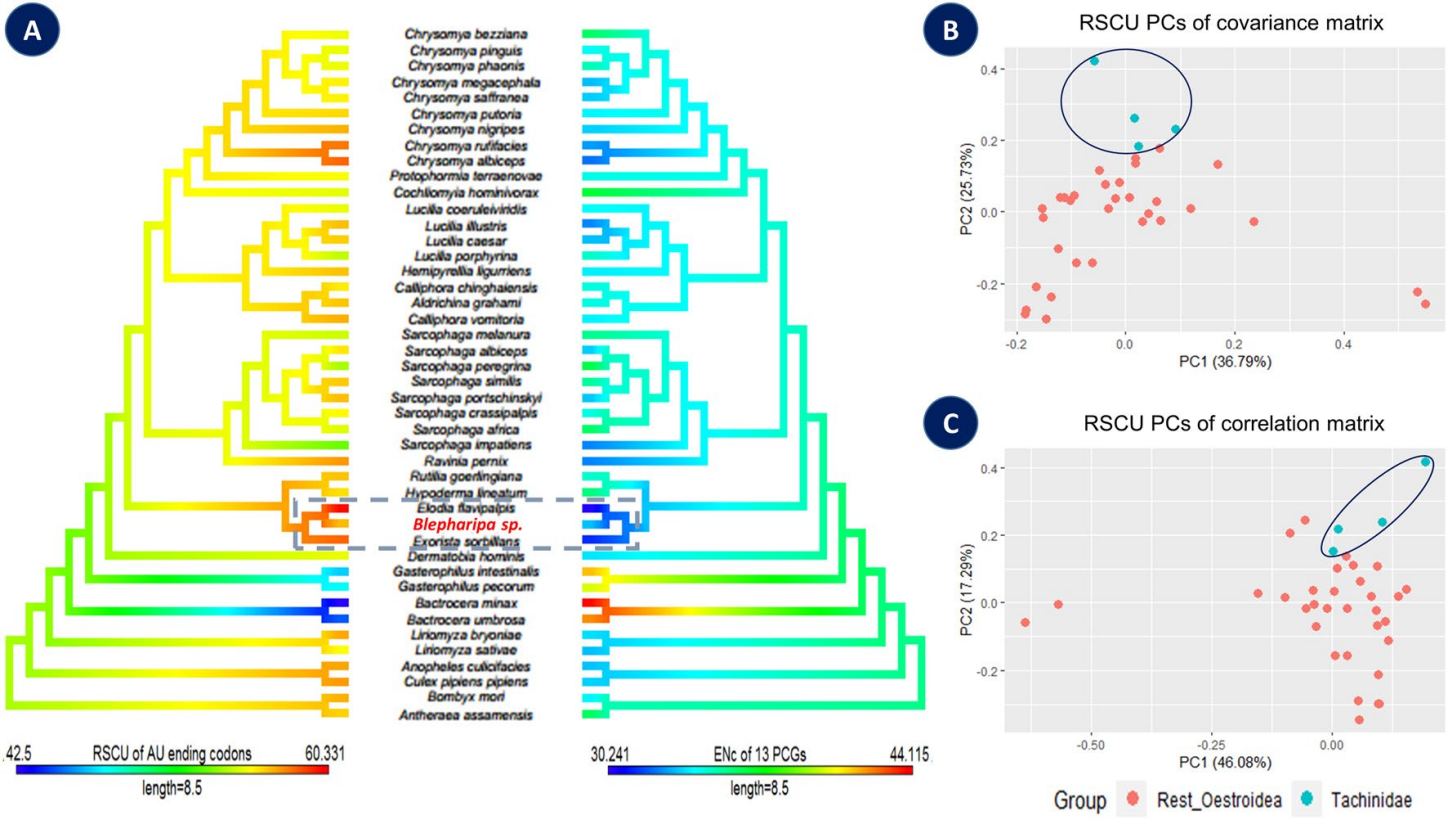
(**A**) RSCU value of AU ending codons and ENc of 13 concatenated PCGs. Contour map phylogeny shows the estimated evolutionary history of codon usage, and corresponding variation of ENc produced via contMap function in the R package Phytools. Note that the Tachinidae clade has evolved a AT content that is higher than the rest of the ingroup that is reflected in ENc. (**B**) and (**C**) Principal components analysis of RSCU across the Oestroidea. The Tachinidae groups are distinguishable from rest of the Oestroidea insects.

According to our findings, Tachinidae is the only obligate insect endoparasite family in the Oestroidea superfamily with significantly AT biased PCGs and a high proportion of AT in codons of their mitogenome. Interestingly, the Gasterophilinae tribe of the Oestridae family, which is an internal parasite of mammals, has the lowest A + T content, and its clade is phylogenetically split before other Oestroidea flies diverged (Figs. 8, 11)^214^. Tachinidae flies, a sister clade of the Oestridae family, as well as two other Oestridae flies, *H. lineatum* and *D. hominis*, have AT-rich genes. Despite being in the same lineage as *R. goerlingiana*, *H. lineatum* is an external parasite, whereas *D. hominis* is an endoparasite. Apart from that, other Oestroidea flies included in this study all show ecto-parasitism (Table 1). Consistent with the tendencies seen in the base composition, we found that the Tachinidae's codon usage was biased toward high AT content (Fig. 5B). As a result, a link between AT ending codons and ENc is found in the Tachinidae family, where the codon usage of AT ending codon is higher, but the effective number of codons (ENc) is lower, as shown in the contour map color scheme (Fig. 11A, see Fig. S10 in Supplementary Note). We also conducted a principal component analysis of concatenated 13 PCGs RSCU values using covariance matrix and correlation matrix where Tachinids are distinguishable from the rest of Oestroidea flies through the first two principal components (PC1 and PC2) of both the matrices (Fig. 11B,C).

It has been frequently reported that synonymous codon changes do not alter the protein sequence. Still, it can substantially impact on protein levels, folding, translation efficiency, and gene expression of other organisms^28,30,242–244^. The ENc and neutrality plots revealed that directional mutations and selection forces had a role in shaping Oestroidea's mitogenome during evolution. The AT-rich mitochondrial PCGs of endo-parasite tachinid flies are the consequence of mutational bias towards A/T ending codons, whereas natural selection has shaped biased codon usage in PCGs by constraining GC content (Figs. 5, 7). Although the narrow distribution GC3, the deviation of points from the ENc standard curve in the ENc-plot, and the ω value of less than 1 in non-synonymous substitution analysis all imply that mutation bias is not the primary factor driving codon bias^116,212,227^. This study also infers that PCGs have high purifying selection due to a higher synonymous divergence rate than nonsynonymous divergence rate. Tachinidae flies have a much higher AT concentration in the 3rd codon position than other species, with the lowest AT level being 87.9% in *nad6* of *R. goerlingiana* and the highest being 100% in *atp8* of *E. flavipalpis*, resulting in a less effective number of codons (Supplementary Data 2B). The purifying selection appears to be the major evolutionary force, as it efficiently eliminates harmful adaptive changes in amino acids and reduces the effects of adaptive selection pressures at the codon level, despite the fact that directional mutations caused considerable AT-usage bias. According to a previous study on mitogenome evolution, strongly locomotive species rapidly eliminate detrimental non-synonymous substitutions, indicating that they were subjected to intense purifying selection to maintain effective respiratory-chain activity^245^. During this process, Synonymous substitutions were maintained, and directional mutations resulted in specific types of codons being used more frequently^212,245^. Altogether it leads to high usage of synonymous codons in Tachinids than in other Oestroidea flies. Hence, efficient energy production by mitochondria of Tachinids needs fewer codons, that might assist in maintaining their gene expression, translation, or further protein folding and function^28–31,203,204^.

Various hosts react differently to parasitic infections, and while arthropods or insects do have innate immunity, they show very little adaptive immune response compared to mammals^246^. Larva of other Oestroidea flies included in this study are generally necrophagous, saprophagous, or sarcophagus ectoparasite and feed on carrion and carcass of a broad range of vertebrates (Table 1)^247^. On the other hand, tachinid flies are typical internal parasitoids with specialized in their host choice (insect)^248,249^. During larval stages, the feeding maggots constantly tackle the host defense mechanism that builds up a highly stressful environment for the larvae^151,248^. Additionally, Tachinids have to thrive as endo-parasites in highly dioxic environments by adopting a unique respiration strategy^2,42^. We postulate that Tachinidae flies have naturally selected for limited GC content and purifying selection to preserve mitochondrial functions, as well as mutational pressure towards biased AT content to reduce the number of effective codons, resulting in a higher rate of energy synthesis at a lower cost^212,245,250–252^. This, in turn, provides a selective energetic advantage to the Tachinids in surviving the hostile environment of the host^253^.

## Conclusion

The complete mitogenome of *Blepharipa* sp. has been sequenced and annotated to describe its characteristics at the molecular level. This study deliberates on gene orders, gene length, noncoding regions (control region, intergenic spacers), nucleotide composition, and codon usage of *Blepharipa* sp. mitogenome. In general, the features found in the mitogenome of *Blepharipa* sp. are similar to other previously studied tachinid flies^32,42^. The mitogenome arrangement among Sarcophagidae and Tachinidae is consistent with ancestral type, but some of the members of Calliphoridae and Oestridae have undergone tRNA rearrangements which have further led to the formation of a unique intergenic spacer and the overlapping region at their adjoining areas. Tachinid flies have a shorter mitogenome than other Oestroidea flies since the control region might not have been adequately covered with current sequencing and assembly methods due to the presence of extreme AT richness and repetitive sequences at CR. One important finding of the current comparative study is that *Blepharipa* sp. and its family Tachinidae, contain a relatively higher proportion of A + T nucleotides in their mitogenome and consequently, possess AT biased codons in their protein-coding genes. The role of natural selection is found to be a major factor in determining organisms' synonymous codon usage bias rather than mutation pressure, as proven by other studies^116^. Within mitochondria, the longer genes (*nad5, nad4, nad1, cox1*) possess the most biased codons than the shorter genes, and this phenomenon is equally observed in the intron less genes of prokaryotes^33,34^. This study shows the significant usage of AT-rich codons by Tachinids, which limits the use of other codons. Tachinidae are also distinguished from the rest of the Oestroidea insects by principal component analysis of RSCU values. Further, the phylogenetic analysis based on protein-coding genes (PCGs) shows well-supported monophyly of the Sarcophagidae and Calliphoridae family, whereas Tachinidae and Oestridae encountered some irregularities and non-monophyly of taxa. Additional mitogenome sequencing data and a wider taxon sample are necessary to get an absolutely resolved Oestroidea phylogeny, particularly for the Tachinidae family, as it is one of the largest families of species existing on Earth.

The lineage wise nucleotide substitution analysis shows strong purifying selection on mitochondrial genes, although the branch leading to the uzi flies' common ancestor has gained more nonsynonymous mutations than synonymous mutations, and therefore putting more selective pressure on it than other branches. We believe that the signal for positive selection is usually drowned out by relaxed selection because positive selection often occurs on a few sites for a short period of evolutionary time, and therefore the value of ω is always less than 1.0 in either foreground or background branches. This study also shows that the nonlinear model fitted better to deduce the relationship between divergence rate and codon usage indices. Where, synonymous and nonsynonymous divergence rates exhibit opposite S-curve-like relationships with GC3 and GC3s, respectively, and we argue that both divergence rates will eventually form an S-curve with AT3. The divergence rate forms a valley-shape relation with ENc where the rate of divergence first decreases rapidly then again gradually increases, although the intensity of the synonymous divergence rate is higher than the nonsynonymous divergence rate.

Overall, the mitogenome reported here will serve as a useful dataset for studying the genetics, systematics, and phylogenetic relationships of many species, the Tachinidae family, in particular, and uzi flies flies, in general. Therefore, along with the completion of *Blepharipa* sp. mitogenome sequencing and documentation; a series of these extensive comparative analyses with related Oestroidea flies can open new aspects of insect mitogenome research.

## Supplementary Information

Supplementary Information 1.

Supplementary Information 2.

Supplementary Information 3.

Supplementary Information 4.

Supplementary Information 5.

Supplementary Information 6.

Supplementary Information 7.

Supplementary Information 8.

Supplementary Information 9.
