# Supplementary material for: Mitogenome-wise codon usage pattern from comparative analysis of the first mitogenome of Blepharipa sp. (Muga uzifly) with other Oestroid flies
Source: Sci Rep. 2022 Apr 29;12:7028. doi: 10.1038/s41598-022-10547-8 (PMC9054809; doi:10.1038/s41598-022-10547-8)
Supplement: Supplementary file 1 — Supplementary Information 1. [file 41598_2022_10547_MOESM1_ESM.pdf]

Mitogenome-wise codon usage pattern from comparative analysis of the first mitogenome of *Blepharipa sp.* (Muga uzifly) with other Oestroid flies

Debajyoti Kabiraj[a], Hasnahana Chetia[a], Adhiraj Nath[a], Pragya Sharma[c], Ponnala Vimal Mosahari[b], Deepika Singh[a], Palash Dutta[d], Kartik Neog[d], Utpal Bora[a, b*]

[a] Bioengineering Research Laboratory, Department of Biosciences and bioengineering, Indian Institute of Technology Guwahati, Assam, India

[b] Centre for the Environment, Indian Institute of Technology Guwahati, Assam, India

[c] Department of Bioengineering and Technology, Gauhati University Institute of Science and Technology (GUIST), Gauhati University, Guwahati, Assam, India

[d] Biotechnology Section, Central Muga Eri Research & Training Institute (CMER&TI), Lahdoigarh, Jorhat, Assam, India

*Corresponding author: ubora@iitg.ac.in

No.	Supplementary Note	Page No.
1.	Supplementary Method	2
2.	Supplementary Table	8
3.	Supplementary Figure	13

1. **Supplementary Method:**

- **Sequence Validation through PCR:**

PCR reactions were performed using synthesized primer sets using isolated DNA as template in ABI Veriti 96 well thermal cycler. The 12.5 µl of EmeraldAmp GT PCR Master Mix (cat # RR310A), 1.0 µl each 10 pmole/µl forward and reverse primers with 20 ng of DNA was used to carry out PCR amplification in final reaction volume of 25 µl. The reaction conditions were 3 min at 95°C followed by 35 cycles of 20 sec at 98°C, 30 sec at 55°/ 60°/65° and 45 sec at 72°C, ending with a final extension step of 72°C for 10 min.

All PCR products were sequenced by the ABI sequencer 3730xl at Xcelris lab (Ahmedabad, India). The amplicons were purified with ExoSAP (USB) and subjected to automated DNA sequencing on ABI 3730xl Genetic Analyzer (Applied Biosystems, USA). Sequencing was carried out using Big Dye Terminator v3.1 Cycle sequencing kit following the manufacturer's protocol, where sequencing cycle was set with the thermal ramp rate of 1°C per second for 30 cycle.

Submitted *Blepharipa sp. nad6* gene at NCBI

```
>KY644698.1:10055-10579 Blepharipa sp. CMERI-Uzi-001 mitochondrion, complete
genome
ATTATACAATGAATTTTATTTTCTTTAATATTTTTATTTAATTTTATATTTATTTTTATAAAACATCCCT
TAGCTATAGGATTAACCTTATTAATTCAAACTACATTAATTTCTCTTATATCAGGATTAATTCATAAAAC
TTTTTGATTTTCTTATATTTTATTTTTAATTTTTTTAGGAGGAATATTAGTATTATTTATTTATGTAACT
TCTTTAGCATCAAATGAAATATTTAATCTTTCAATTAAACTATTTATAATTTCTTTCATAATATTAATTA
CATTTTTATTAATTATAATTTTTATAGACAAAAATATAATACTTCAATATAAAAATAATGAAATTTTATC
CATTATCAATTTAAATTCTTATATTATAGAAAATTCCTTATCAATTAATAAATTATATAATTTTCCTACA
AATTTAATAACAATTTTATTAATAAATTATTTATTAATTACATTAATTGCTATCGTAAAAATTACTAAAT
TATTTAAAGGTCCTTTACGACCTATATTTAACTAA
```

Primer sequences used for Amplification of *nad6* gene

No.	Primer name	Primer sequence
1	NAD6-For	**CATTGGTCTTGTAAATCAAAAATAAGT**
2	NAD6-Rev	**AAATATTAATAGGAGTTGGTAAATCA**

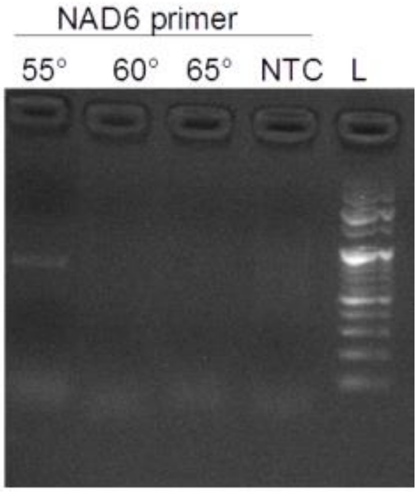

Figure S1: Agarose gel (1.5%) electrophoresis of PCR products and detection of *nad6* gene. Ladder (L): 100bp marker, NTC: no template control and other three lanes are showing PCR products at three different temperatures (65°, 60°,55°). Single amplification band observed on 55° C. (NAD6 gel run was done separately from D-loop and CR).

	**Outcome after sequencing**	**Percentage identity in Clustal omega**
1	**>SAMPLE_NAD6_CUS-764_H12_082**<br><br>ACAATATAATGATCTATTAATAATTTTTAAATTGGGTGTTTAACTCGTAGGGGTTTATTCATTA<br>TTTAAATATAGGTCGTAAAGGACCTTTAAATAATTTAGAAATTTTTACAATAGCAATTAAGGTA<br>ATTAATAAATAATTTATTAATAAAATTGTTATTAAATTTGTAGGAAAATTATATAATTTATTAA<br>TTGATAAGGAATTTTCTATAAAATAAAAATTTAATTTGATAAGGGATAAAATTTCATTATTTTT<br>ATATTGAAGTATTATATTTTTGTCTATAAAAATTATAATTAATAAAAATGTAATTAATATTATG<br>AAAAAAATTATAAATAGTTAAATTGAAAAATTAAATATTTCATTTGAGGCTAAAAAAGTTACAT<br>AAATAAATAATACTAAAATTCCCCCTAAAAAAATTAAAAATAAAATATAAAAAAATCAAAAAGT<br>TTTATGAATTAATCCTGATATAAAAGAAATTAAGGTATTTTGAATTAATAAGGTTAACCCAATA<br>GCTAAGGGATGTTTTATAAAAATAAATATAAAATTAAATAAAAATATTAAAGAAAATAAAATTC<br>ATTGTATAATATCAAGAAATAGTTAAAATAAAATATTAATTTGGGGGATTAAGGATAAGAAATT<br>CTCTTTTTCTTGAAGTTTTAAAAGGTAAACTTATTTTTGATTTTACAAGACAAAGGGCCGGCGG<br>CGGGTTTTACAACAAAGGGAGAAG	**57.46**
2	**>SAMPLE_NAD6_CUS-764_G12_084**<br><br>AGCGTCCAATAATTTAAACAAAGACATTCAAGAAATTACCTTTTAAACTTCGGAAAAGATTTTC<br>TTATCATTAACCCCCAAAATTAAAATTTTATTTAAACTATTTCTGGATATTATACAAGGAATTT<br>TATTTTCTTTAAAATTTTTATTTAATTTTATATTTATTTTTATAAAACATCCCTAACCTATGGG<br>ATTAACCTTATAAATTCAAACTACTTTAATTTCTCTAATATCAGGATTAATTCAAAAAACTTTT<br>GGATTTTCTAATATTTAATTTTTAATTTTTTAAGGAGGAAAATTAGTATTATTTATTTATGTAA<br>CTTCTTTAGCATCAAAGGAAATATTTAACCTTTCATTTAAACTATTTATAATTTCTTTCAAAAA<br>ATTAATTACTTTTTTATAAATTATAATTTTTATAAACAAAAATATAAAACTTCAAAATAAAAAT<br>AAGGAATTTTTATCCATTATCAATTTAAATTCTAATATAATAGAAAATTCCTTATCAATTAAAA<br>AATTATATAATTTTCCAACAAATTTAAAAACAATTTAATTAATAAATAATTAATAAATAACTTA<br>AATTGCTATCGAAAAAATTACAAATTAATTTAAAGGTCCTTTACAACCTATATTTAACTAAGGA<br>ATAAACCCTTACGAGTTAAACACCCAATTTTAAAAATTATAAATAGATCATTAATTGATTTACC<br>ACTCTCCTATAAAAATTGGTCAAGCCTTGTTTCCGGGGA	**91.81**

Submitted *Blepharipa sp.* Control Region at NCBI

```
>KY644698.1:15025-15080,1-112 Blepharipa sp. CMERI-Uzi-001 mitochondrion,
complete genome
TAAAAACTTTTTAATTTTTTTTTAAAAAAAAAAAAAAAAAAAAAAATTGTAAATTATTTTCTTGTATAAA
AATAGATTTTTTTATTAATAATATAAAATTCAACTAATAAAAAACATGGAAAATTTATTAATAACAAAAA
AAAAAAAAAATGGATTTTTCATTCAAAA
```

Primer sequences used for Amplification of Control Region:

No.	Primer name	Primer sequence
1	D-loop-For	**GTATAACCGCGAATGCTGGCACAA**
2	D-loop-Rev	**GGGTATGAACCCAGTAGCTTGATTAGC**
3	CR15fwd	**TACACATCGCCCGTCACTCT**
4	CR08rev	**TTAACGGCCAAGCGCCTTT**
5	CR_int_fwd	**CTTTGGGAGTTGCAAATGTAAGTG**
6	CR_int_rev	**TTTAACCCTCTCTCCTGGTTTACA**

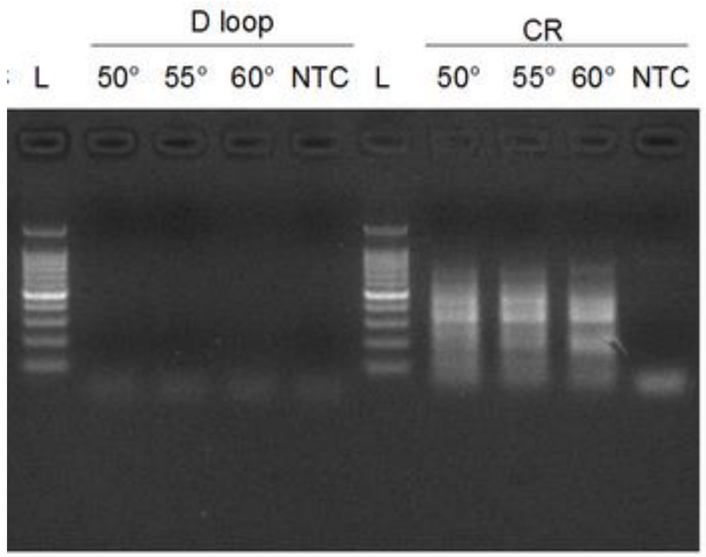

Figure S2: Agarose gel (1.5%) electrophoresis of PCR products and detection of Control Region. Ladder (L): 100bp marker, NTC: no template control and other three lanes are showing PCR products at three different temperatures (65°, 60°,55°). Using two forward and reverse primers (D-

loop primers) do not show amplification, whereas using two additional internal primers (CR primers) show multiple amplifications. (D-loop and CR gel run were done separately from NAD6).

	Outcome after sequencing	Percentage identity in Clustal omega
1	**>Sample_CRintF_CUS-764_C12_C12**<br><br>AGGGAATAGACAGGGGCAGGAAAAGCAACGCCATTGTCCACTGAAAACGA<br>GGTGTACTCTTTTTCCCCCGAAAAAAAACCGAGCGTCGGCGGTTCTTGAA<br>TCACTTACTTCCTGTCATTTTGATTCCTTTTTTCCACTTAACGGAAAAAA<br>AATTACCAAGGGACACAAAGCCGTTATCGCCGTTCAACTAAGGGAGTTAA<br>TTGACCGATTTTCATTGGCGCCCCAACTATCAGTGTGTTGTTGAGGGACC<br>GCGTTTTGGATTTCATCAACACATGGGGCAAACCA	**46.34**
2	**>Sample_CRintR_CUS-764_D12_D12**<br><br>AGTAAAAATTTTATGAGAATTAAAACAGGAAAATCGTTGACGATGGGACC<br>GGAAAATTTCAAATCACCCTTTGAGATTTTAGCCCATGTGTTGATGAAAT<br>ACCAAAACGCGGTACCATCAACAACAGAATAATTGAGGGCGCACCAATGA<br>AATTCCGTCAATTAACTCCGTTACTTGAACGGCGAAAACGGCTTTGTTGT<br>CCCTTGGAATTTTTTAATCCGTTAAGTGAAAAAAAGAATAAAAATAGGGG<br>GAAAATAGTTGATTAAAAAATCTCGCCGACGCTAATATCTTTACTTCGGC<br>GGAAATAAAGAGTGCCCTTCGATATTCATTGAGGAATAAAAACGGTGTTA<br>TCTTCTGATTTGTACATTGAGACCCCCTTTAAGGGAGAAATATTCAAGAG<br>AAAAAAAAGGATTGATAGGGTTGAAT	**53.42**

- **Mapping of Illumina reads with Control Region, *nad6* and *cox2*:**

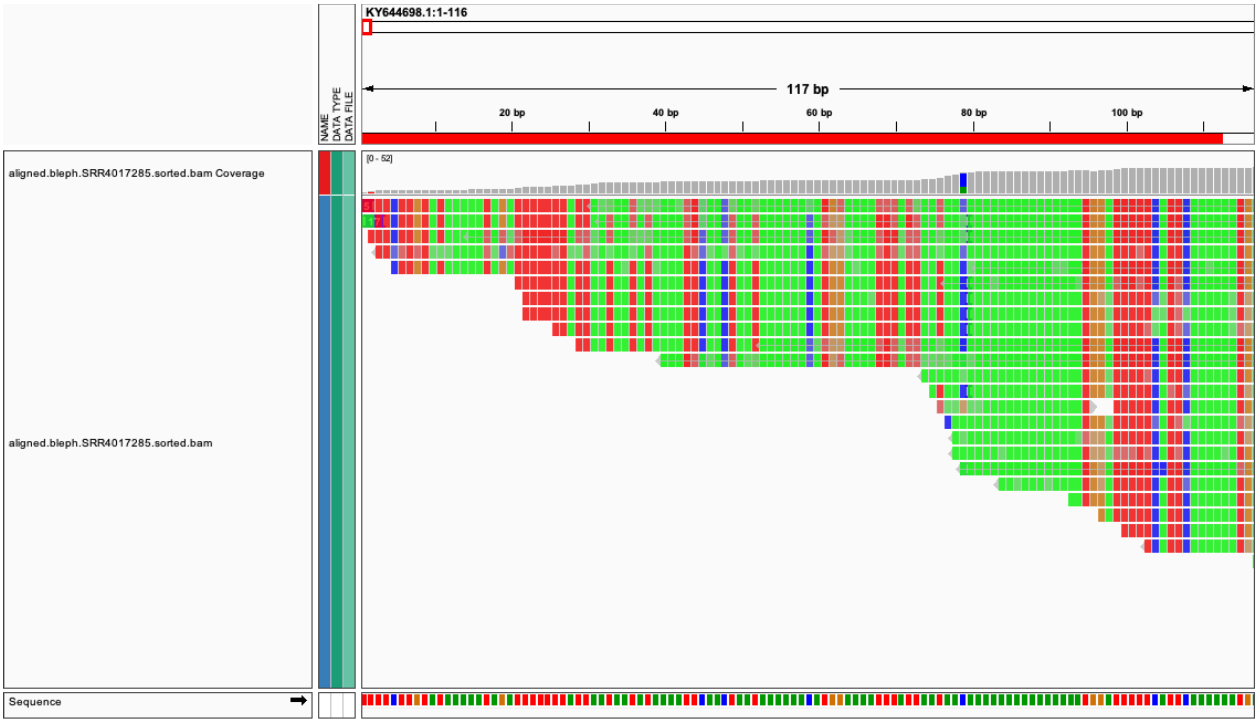

Figure S3: Alignment of Control Region (1-112) with raw reads using Bowtie2 (v 2.4.4). Depth over 1-112 ranged from 5-20X.

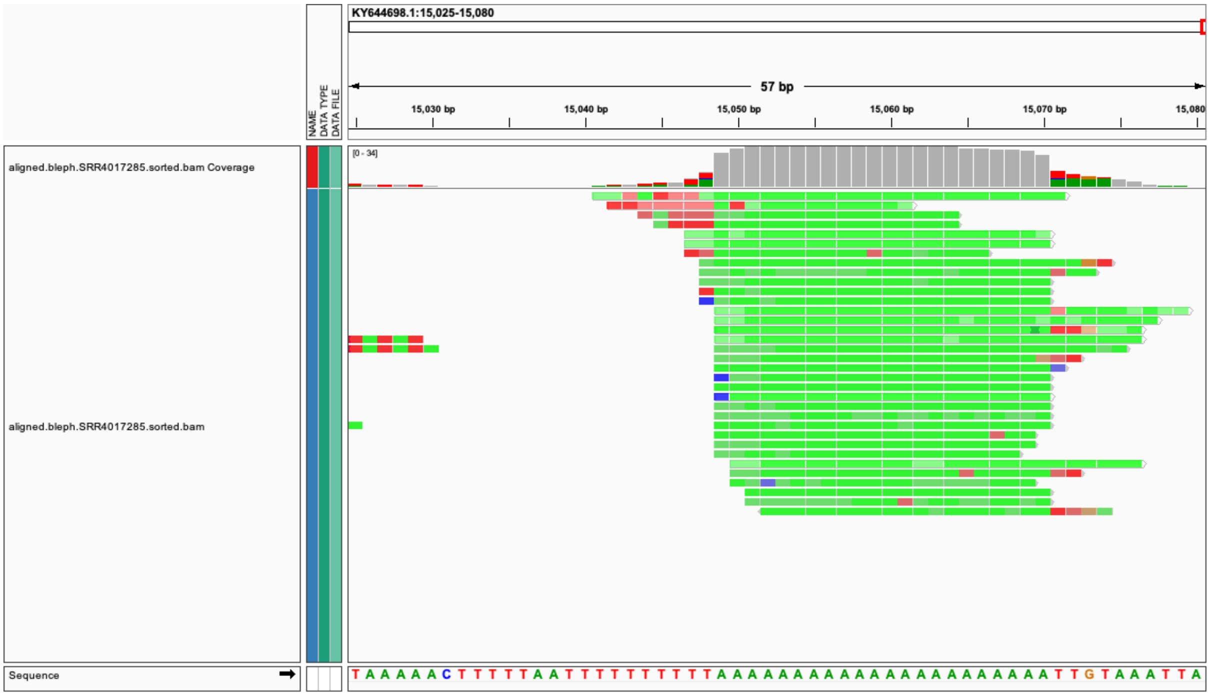

Figure S4: Alignment of Control Region (15025-15080) with raw reads using Bowtie2 (v 2.4.4). Depth over (15025-15080) bp was around 30X, although the raw reads didn't cover a 10 bp stretch of CR.

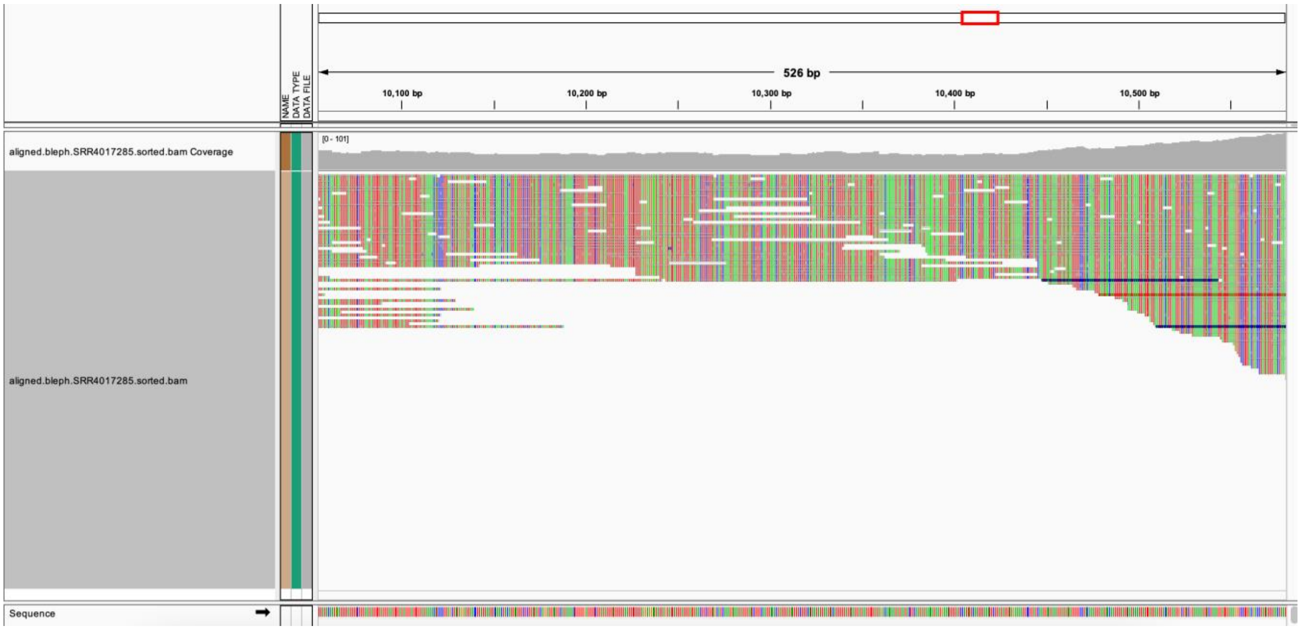

Figure S5: Alignment of *nad6* (10055-10579) with raw reads using Bowtie2 (v 2.4.4).

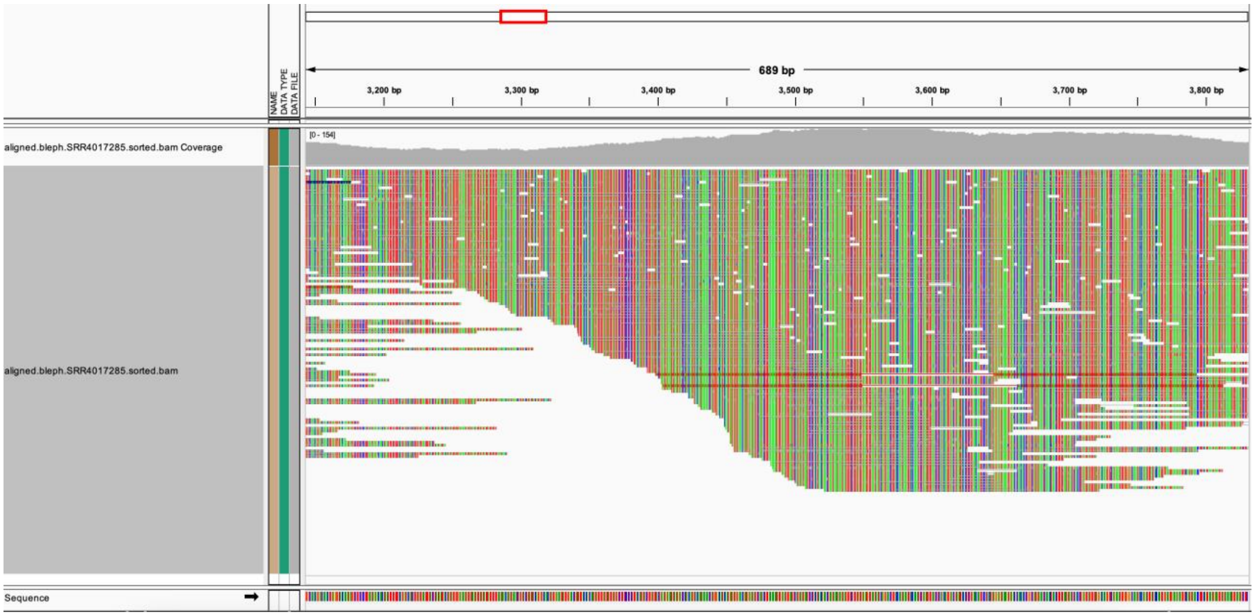

Figure S6: Alignment of *cox2* (3143-3830) with raw reads using Bowtie2 (v 2.4.4).

2. Supplementary Table:

Detection of non-synonymous mutation through branch-specific (Foreground and background branch) model in 13 mitochondrial PCGs using PAML

kappa (ts/tv, Transition/ Transversion) = k

omega (dN/dS, nonsynonymous/synonymous) = ω

One ratio model = One nonsynonymous/synonymous rate ratio (ω) for all lineages of reference phylogenetic tree;

Two ratio model = Two ω value, ω1 for foreground lineage of interest and ω0 for background linages of reference phylogenetic tree;

lnL = Log likelihood of the model estimation; p = Number of parameters;

Table S1: Branch-specific assessments of selective pressure on the common ancestor of *Blepharipa sp.* for the *atp6* gene

**Gene**	**Model**	**p**	**lnL**	**2delL**	**k**	**ω0**	**ω1**
atp6 Gene tree	One ratio: ω0 = ω1	2	-10190.897083	2(l1-l0) = 2 (-10190.178760 + 10190.897083) = **1.436646** 0.1000 < p < 0.5000	1.24910	0.03763	= ω0
	Two ratio:	3	-10190.178760		1.24941	0.03745	0.09387 **2.5**
atp6 Species tree	One ratio: ω0 = ω1	2	-10728.417333	2(l1-l0) = 2 (- 10728.278583 + 10728.417333) = 0.2775 0.5 < p < 0.9	1.24277	0.04173	= ω0
	Two ratio:	3	-10728.278583		1.24278	0.04164	0.06324

Table S2: Branch-specific assessments of selective pressure on the common ancestor of *Blepharipa sp.* for the *atp8* gene.

**Gene**	**Model**	**p**	**lnL**	**2delL**	**k**	**ω0**	**ω1**
atp8 Gene tree	One ratio: ω0 = ω1	2	-2616.647018	2(l1-l0) = 2 (- 2616.646982 + 2616.647018) = **0.00007199** **p < 0.9750**	1.59798	0.11541	= ω0
	Two ratio:	3	-2616.646982		1.59797	0.11541	**0.93957 8.1**
atp8 Species tree	One ratio: ω0 = ω1	2	-2846.231030	2(l1-l0) = 2 (- 2846.100715+ 2846.231030) = 0.26063 0.5 < p < 0.9	1.44523	0.12904	= ω0
	Two ratio:	3	-2846.100715		1.44411	0.12828	0.19493

Table S3: Branch-specific assessments of selective pressure on the common ancestor of *Blepharipa sp.* for the *cox1* gene.

Gene	Model	p	lnL	2delL	k	ω0	ω1
cox1 Gene tree	One ratio: ω0 = ω1	2	-18758.100024	2(l1-l0) = 2 (-18755.804334+ 18758.100024) = 4.59138	1.96021	0.02328	=w0
	Two ratio:	3	-18755.804334	0.01< p < 0.05	1.95964	0.02304	0.07795 **3.38**
cox1 Species tree	One ratio: ω0 = ω1	2	-18325.596341	2(l1-l0) = 2 (- 18324.839205 + 18325.596341) = 1.514272	2.06464	0.02035	= ω0
	Two ratio:	3	-18324.839205	0.1 < p < 0.5	2.06461	0.02025	0.04451 **2.19**

Table S4: Branch-specific assessments of selective pressure on the common ancestor of *Blepharipa sp.* for the *cox2* gene.

Gene	Model	p	lnL	2delL	k	ω0	ω1
cox2 Gene tree	One ratio: ω0 = ω1	2	-8441.416676	2(l1-l0) =2 (-8441.263736 + 8441.416676) = 0.30588	1.89288	0.03392	= ω1
	Two ratio:	3	-8441.263736	0.5 < p <0.9	1.89259	0.03380	0.04635
cox2 Species tree	One ratio: ω0 = ω1	2	-8540.178788	2(l1-l0) = 2 (- 8539.990894 + 8540.178788) =0.375788	1.81732	0.03325	= ω1
	Two ratio:	3	-8539.990894	0.5 < p <0.9	1.81706	0.03315	0.05611

Table S5: Branch-specific assessments of selective pressure on the common ancestor of *Blepharipa sp.* for the *cox3* gene.

Gene	Model	p	lnL	2delL	k	ω0	ω1
cox3 Gene tree	One ratio: ω0 = ω1	2	-10452.838424	2(l1-l0) = 2 (-10452.148006 + 10452.838424) = 1.380836	1.76131	0.04019	= ω1
	Two ratio:	3	-10452.148006	0.1 < p < 0.5	1.76120	0.03995	0.09830 **2.46**
cox3 Species tree	One ratio: ω0 = ω1	2	-10261.120432	2(l1-l0) = 2 (-10260.188753 + 10261.120432) = 1.863358	1.77841	0.03659	= ω1
	Two ratio:	3	-10260.188753	0.1 < p < 0.5	1.77837	0.03632	0.09616 **2.64**

Table S6: Branch-specific assessments of selective pressure on the common ancestor of *Blepharipa sp.* for the *cytb* gene.

Gene	Model	p	lnL	2delL	k	ω0	ω1
cytb Gene tree	One ratio: ω0 = ω1	2	-15331.557893	2(l1-l0) = 2 (-15330.100236 + 15331.557893) = 2.915314	1.67426	0.02992	= ω0
	Two ratio:	3	-15330.100236	0.05 < p < 0.1	1.67401	0.03005	**0.00010**
cytb Species tree	One ratio: ω0 = ω1	2	-16291.467134	2(l1-l0) = 2 (-16290.173814 + 16291.467134) = 2.58664	1.64815	0.03490	= ω0
	Two ratio:	3	-16290.173814	0.1 < p < 0.5	1.64774	0.03470	0.09962 **2.87**

Table S7: Branch-specific assessments of selective pressure on the common ancestor of *Blepharipa sp.* for the *nad1* gene.

Gene	Model	p	lnL	2delL	k	ω0	ω1
nad1 Gene tree	One ratio: ω0 = ω1	2	-11526.891567	2(l1-l0) = 2(-11526.697535 + 11526.891567) = 0.388064	1.26930	0.03016	= ω0
	Two ratio:	3	-11526.697535	0.5 < p < 0.9	1.26924	0.03007	0.04641
nad1 Species tree	One ratio: ω0 = ω1	2	-11861.261140	2(l1-l0) = 2(-11859.950552 + 11861.261140) = 2.621176	1.26618	0.03239	= ω0
	Two ratio:	3	-11859.950552	0.1 < p < 0.5	1.26540	0.03217	0.12056 **3.74**

Table S8: Branch-specific assessments of selective pressure on the common ancestor of *Blepharipa sp.* for the *nad2* gene.

Gene	Model	p	lnL	2delL	k	ω0	ω1
nad2 Gene tree	One ratio: ω0 = ω1	2	-16834.789874	2(l1-l0) = 2(-16827.770725 + 16834.789874) = 14.038298	1.20439	0.06649	= ω0
	Two ratio:	3	-16827.770725	0.0010 < p	1.20397	0.06506	0.15463 **2.37**
nad2 Species tree	One ratio: ω0 = ω1	2	-17686.901083	2(l1-l0) = 2(-17686.567056 + 17686.901083) = 0.668054	1.18905	0.08138	= ω0
	Two ratio:	3	-17686.567056	0.1 < p < 0.5	1.18926	0.08155	0.04346

Table S9: Branch-specific assessments of selective pressure on the common ancestor of *Blepharipa sp.* for the *nad3* gene.

Gene	Model	p	lnL	2delL	k	ω0	ω1
nad3 Gene tree	One ratio: ω0 = ω1	2	-5148.735350	2(l1-l0) = 2 (-5144.735647 + 5148.735350) = 7.999406	1.20069	0.04170	= ω0
	Two ratio:	3	-5144.735647	$0.001 < p < 0.01$	1.19938	0.04028	0.10896<br>**2.7**
nad3 Species tree	One ratio: ω0 = ω1	2	-5547.059518	2(l1-l0) = 2 (-5546.880896 + 5547.059518) = 0.357244	1.09146	0.04920	= ω0
	Two ratio:	3	-5546.880896	$0.5 < p < 0.9$	1.09148	0.04908	0.09414

Table S10: Branch-specific assessments of selective pressure on the common ancestor of *Blepharipa sp.* for the *nad4* gene.

Gene	Model	p	lnL	2delL	k	ω0	ω1
nad4 Gene tree	One ratio: ω0 = ω1	2	-19292.431204	2(l1-l0) = 2 (-19288.107478 + 19292.431204) = 8.647452	1.10768	0.04805	= ω0
	Two ratio:	3	-19288.107478	$0.001 < p < 0.01$	1.10724	0.04755	0.20902<br>**4.39**
nad4 Species tree	One ratio: ω0 = ω1	2	-19670.256978	2(l1-l0) = 2 (-19666.668842 + 19670.256978) = 7.176272	1.09963	0.05014	= ω0
	Two ratio:	3	-19666.668842	$0.001 < p < 0.01$	1.09944	0.04972	0.20965<br>**4.21**

Table S11: Branch-specific assessments of selective pressure on the common ancestor of *Blepharipa sp.* for the *nad4l* gene.

Gene	Model	p	lnL	2delL	k	ω0	ω1
nad4l Gene tree	One ratio: ω0 = ω1	2	-3619.280646	2(l1-l0) = 2 (-3618.772347 + 3619.280646) = 0.50	0.99404	0.05108	= ω0
	Two ratio:	3	-3618.772347	$0.1 < p < 0.5$	0.99415	0.05033	0.07853
nad4l Species tree	One ratio: ω0 = ω1	2	-3689.063362	2(l1-l0) = 2 (-3689.057872 + 3689.063362) = 0.01098	0.95950	0.04794	= ω0
	Two ratio:	3	-3689.057872	$0.9 < p < 0.975$	0.95939	0.04790	0.05301

Table S12: Branch-specific assessments of selective pressure on the common ancestor of *Blepharipa sp.* for the *nad5* gene.

Gene	Model	p	lnL	2delL	k	ω0	ω1
nad5 Gene tree	One ratio: ω0 = ω1	2	-25675.981476	2(l1-l0) = 2 (-25662.473530 + 25675.981476) = 27.015892<br>0.001 < p	1.35147	0.04635	= ω0
	Two ratio:	3	-25662.473530		1.35187	0.04559	**0.92099**<br>**20.2**
nad5 Species tree	One ratio: ω0 = ω1	2	-26541.427350	2(l1-l0) = 2 (- 26536.903264 + 26541.427350) = 9.048172<br>0.001 < p < 0.01	1.32722	0.05192	= ω0
	Two ratio:	3	*-26536.903264*		1.32619	0.05145	0.20860<br>**4.05**

Table S13: Branch-specific assessments of selective pressure on the common ancestor of *Blepharipa sp.* for the *nad6* gene.

Gene	Model	p	lnL	2delL	k	ω0	ω1
nad6 Gene tree	One ratio: ω0 = ω1	2	-9162.084564	2(l1-l0) = 2 (-9160.924827 + 9162.084564) = 2.319474<br>0.1 < p < 0.5	0.97068	0.06215	= ω0
	Two ratio:	3	-9160.924827		0.97045	0.06163	0.15582<br>**2.5**
nad6 Species tree	One ratio: ω0 = ω1	2	-9940.927203	2(l1-l0) = 2 (-9160.924827 + 9940.927203) = 1560<br>p < 0.001	0.90819	0.08094	= ω0
	Two ratio:	3	-9160.924827		0.97045	0.06163	0.15582<br>**2.5**

3. Supplementary Figure:

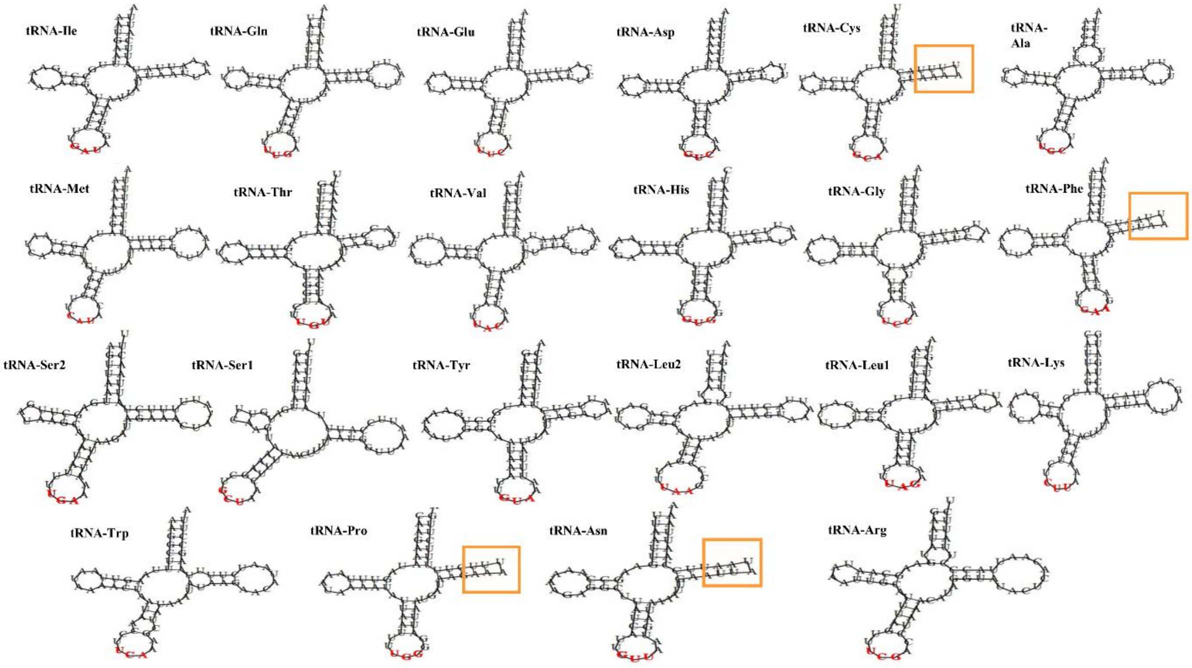

Figure S7: 22 tRNA structures encoded by *Blepharipa sp.* mitogenome. Red color three letter signifies anticodon site and *trnC*, *trnF*, *trnP*, *trnN* lack stable TΨC loop denoted by Yellow box.

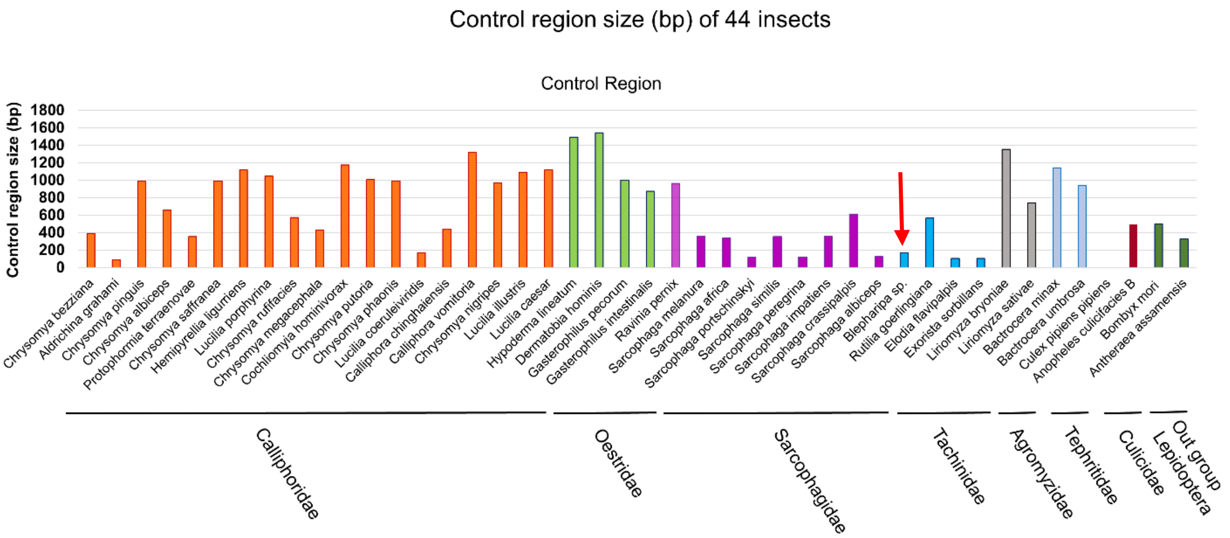

Figure S8: Control Region (CR) Size of 44 Species. Red arrow shows newly sequenced *Blepharipa sp.*s CR.

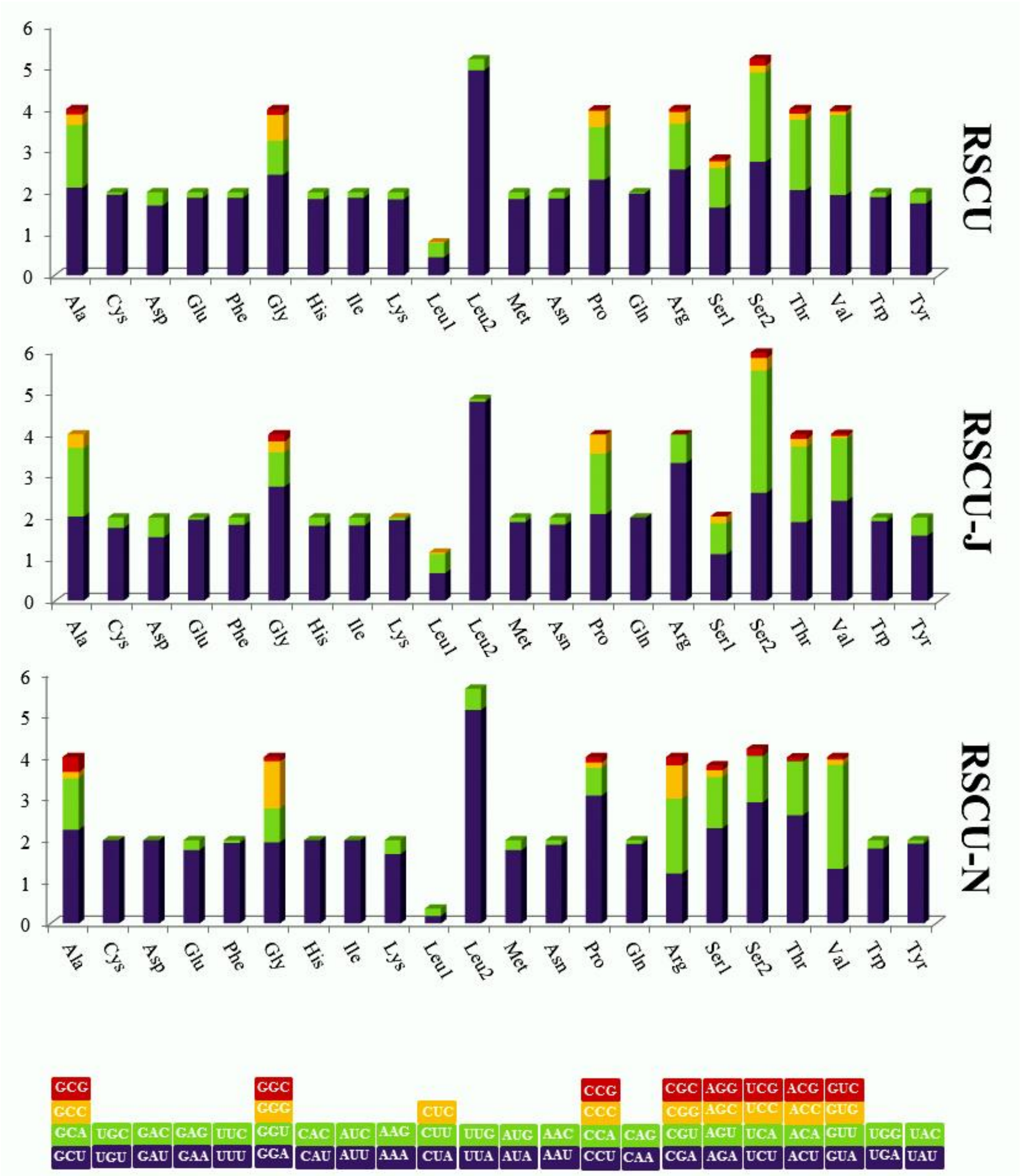

Figure S9: Graph of RSCU value of *Blepharipa sp.* of N and J strand and corresponding amino acid and codons; Y axis: RSCU value; X axis: Amino acid and Codon of *Blepharipa sp.*

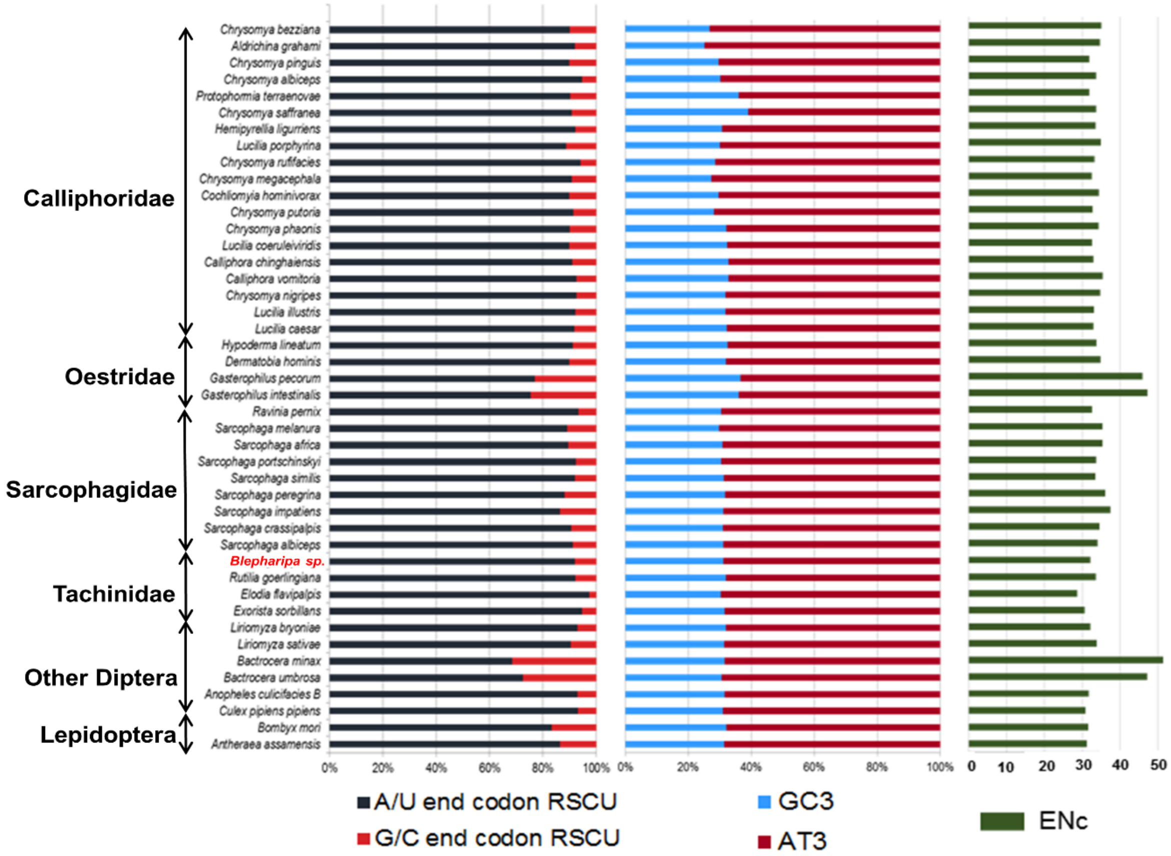

Figure S10: Overall relation between relative synonymous codon usage, 3rd codon position and effective number of codons.

RSCU_A3/T3 vs GC3 and RSCU_G3/C3 vs GC3

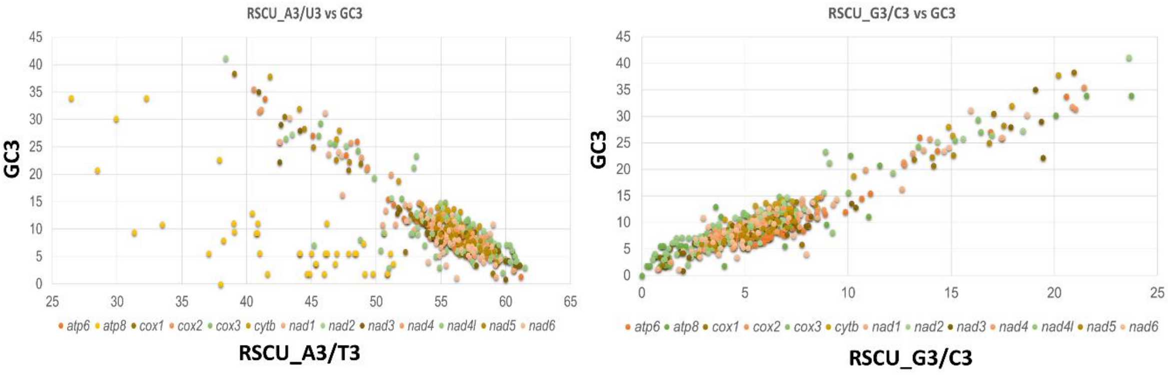

Figure S11: Scatter plot of GC3 vs RSCU value of AT/U ending codons (Left) and RSCU value of GC ending codons (Right).

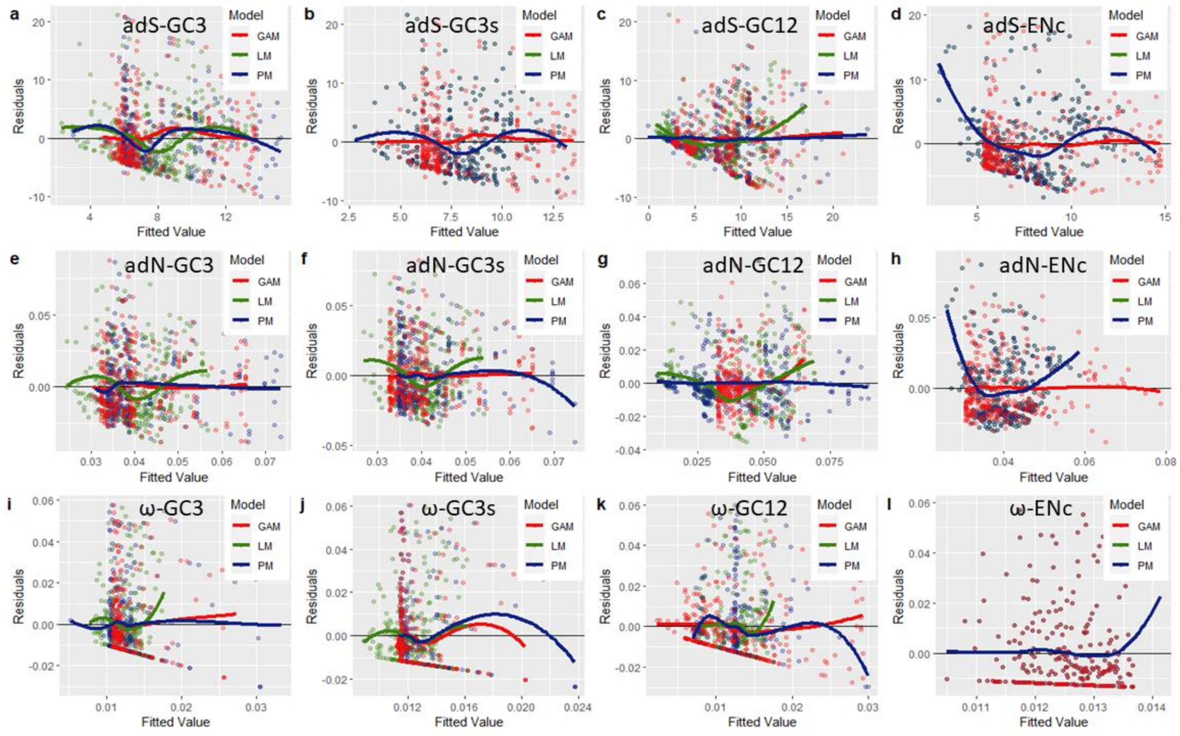

Figure S12: Residuals vs fitted (R-F) plot; (a-d): R-F plots of average synonymous divergence (adS) rate vs predictors (GC3, GC3s, GC12, ENc); (e-h): R-F plots of average nonsynonymous divergence rate(adN) vs predictors (GC3, GC3s, GC12, ENc); (i-l)): R-F plots of omega ratio (ω) vs predictors (GC3, GC3s, GC12, ENc); Green: LM, Blue: PM, Red: GAM.

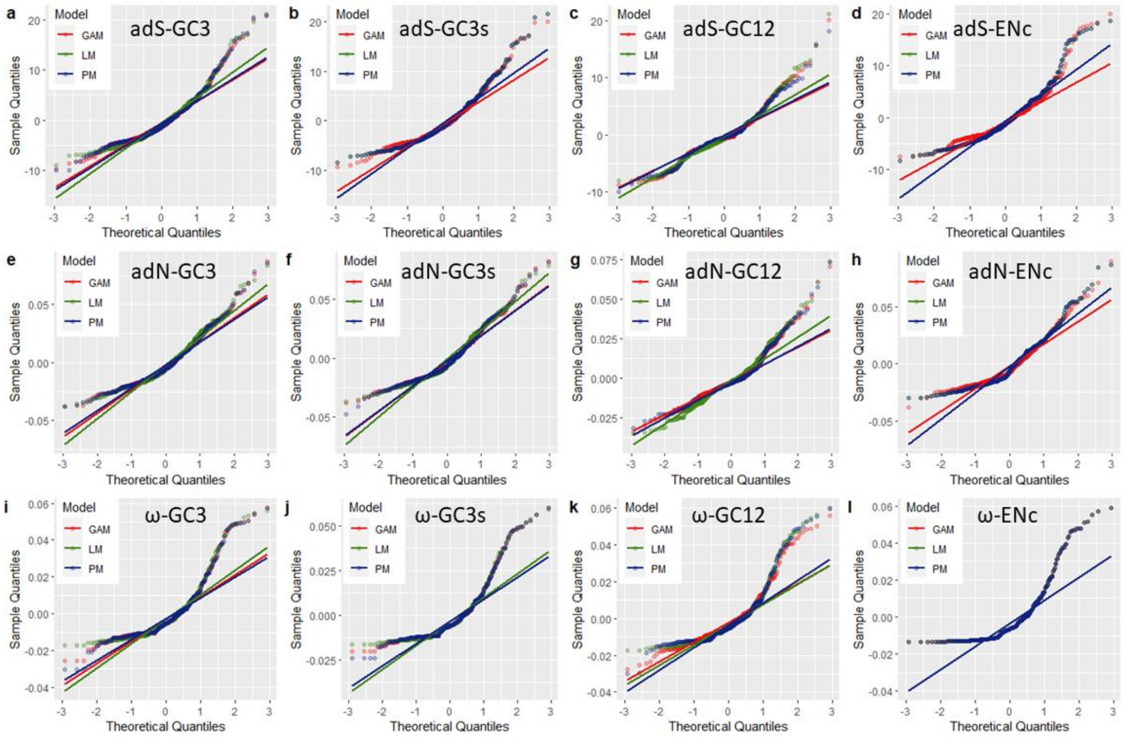

Figure S13: Quantile-Quantile (Q-Q) plot; (a-d): Q-Q plots of average synonymous divergence (adS) rate vs predictors (GC3, GC3s, GC12, ENc); (e-h): Q-Q plots of average nonsynonymous divergence rate (adN) vs predictors (GC3, GC3s, GC12, ENc); (i-l)): Q-Q plots of omega ratio (ω) vs predictors (GC3, GC3s, GC12, ENc); Green: LM, Blue: PM, Red: GAM.

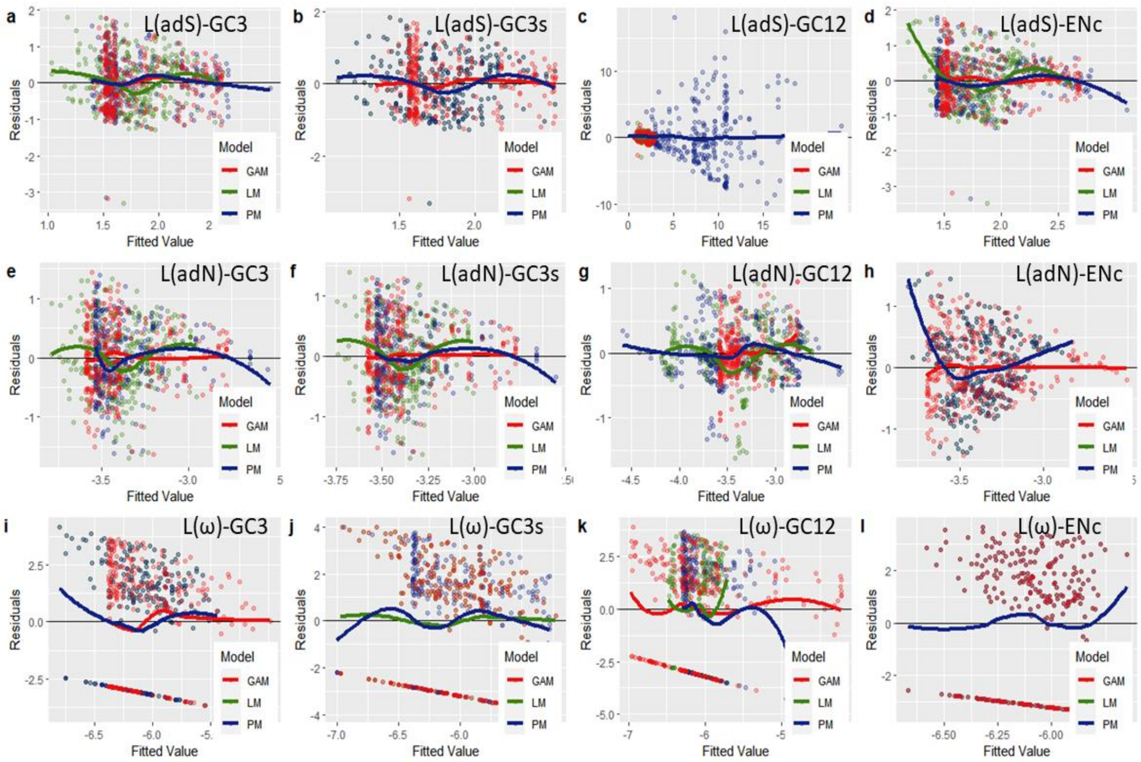

Figure S14: Residuals vs fitted (R-F) plot of log response variables; (a-d): R-F plots of log of average synonymous divergence (L(adS)) rate vs predictors (GC3, GC3s, GC12, ENc); (e-h): R-F plots of log of average nonsynonymous divergence rate (L(adN)) vs predictors (GC3, GC3s, GC12, ENc); (i-l): R-F plots of log of omega ratio (L(ω)) vs predictors (GC3, GC3s, GC12, ENc); Green: LM, Blue: PM, Red: GAM.

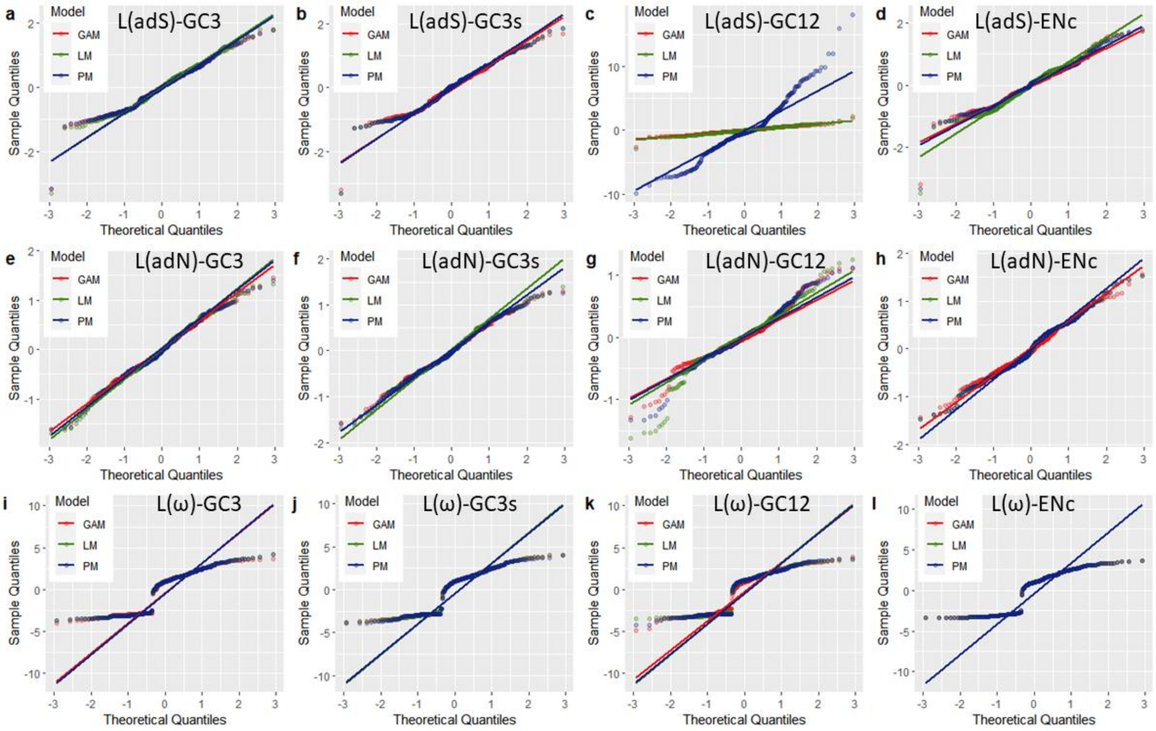

Figure S15: Quantile-Quantile (Q-Q) plot of log response variables; (a-d): Q-Q plots of log of average synonymous divergence (L(adS)) rate vs predictors (GC3, GC3s, GC12, ENc); (e-h): Q-Q plots of log of average nonsynonymous divergence rate (L(adN)) vs predictors (GC3, GC3s, GC12, ENc); (i-l): Q-Q plots of log of omega ratio (L(ω)) vs predictors (GC3, GC3s, GC12, ENc); Green: LM, Blue: PM, Red: GAM.

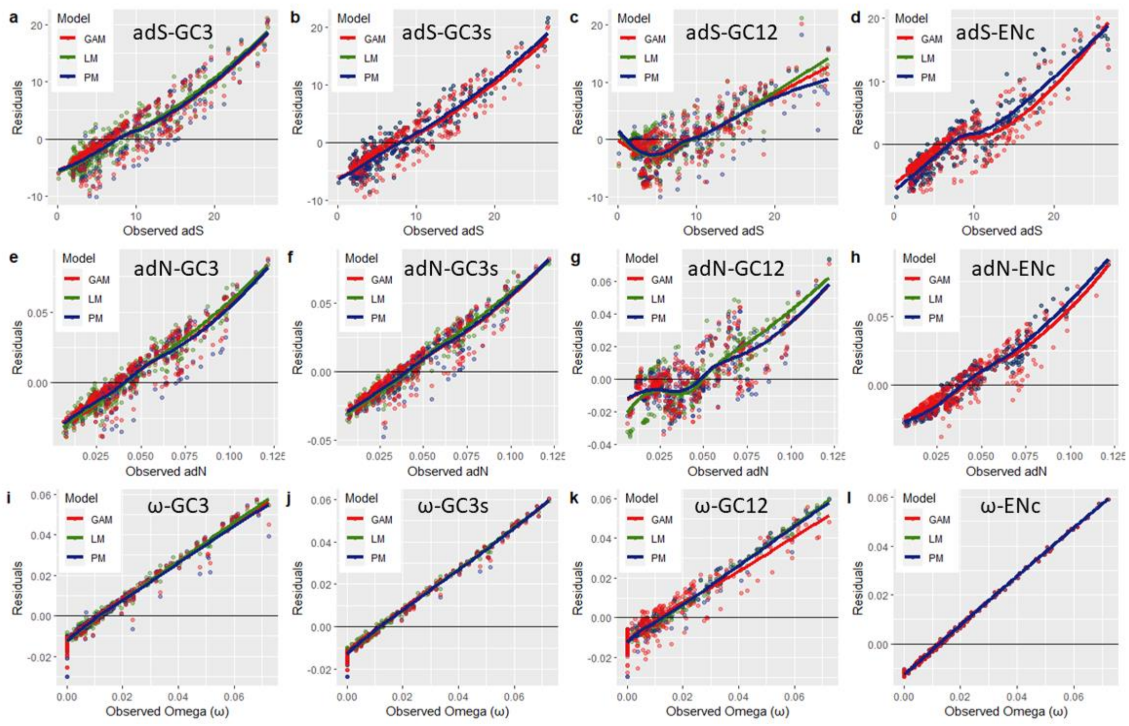

Figure S16: Residuals vs observed (R-O) plot of log response variables; (a-d): R-O plots of log of average synonymous divergence (L(adS)) rate vs predictors (GC3, GC3s, GC12, ENc); (e-h): R-O plots of log of average nonsynonymous divergence rate (L(adN)) vs predictors (GC3, GC3s, GC12, ENc); (i-l): R-O plots of log of omega ratio (L(ω)) vs predictors (GC3, GC3s, GC12, ENc); Green: LM, Blue: PM, Red: GAM.

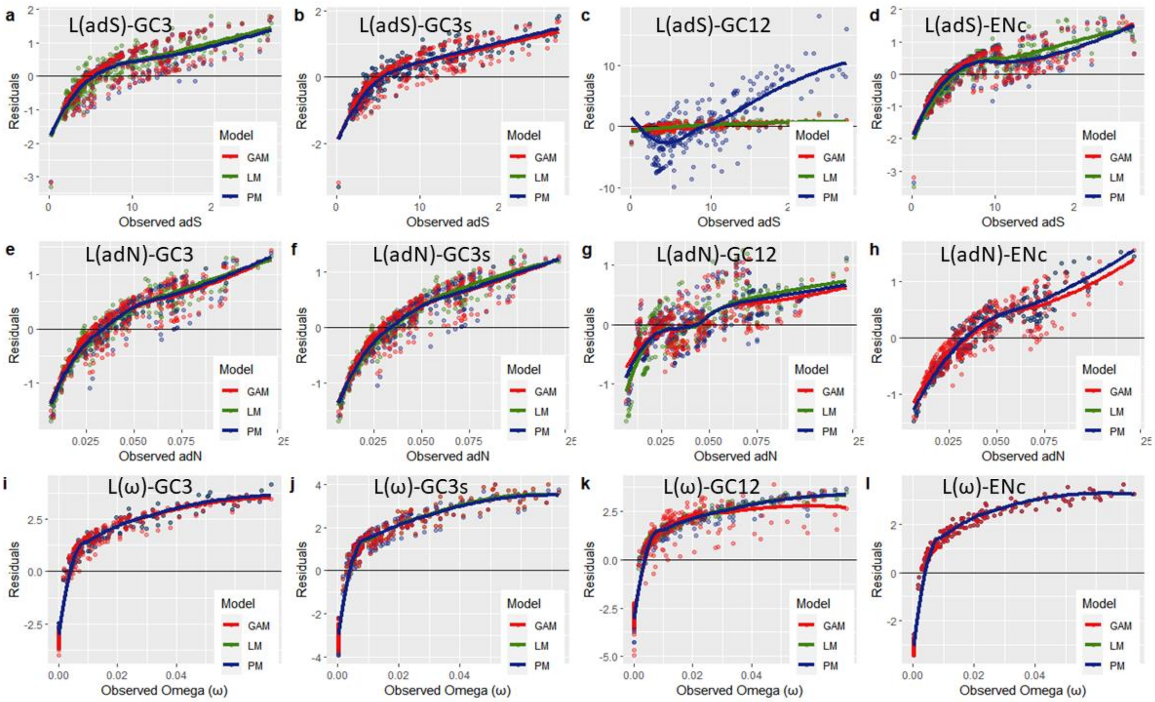

Figure S17: Residuals vs observed (R-O) plot of log response variables; (a-d): R-O plots of log of average synonymous divergence (L(adS)) rate vs predictors (GC3, GC3s, GC12, ENc); (e-h): R-O plots of log of average nonsynonymous divergence rate (L(adN)) vs predictors (GC3, GC3s, GC12, ENc); (i-l): R-O plots of log of omega ratio (L(ω)) vs predictors (GC3, GC3s, GC12, ENc); Green: LM, Blue: PM, Red: GAM.
